# Human perception of avian biodiversity in naturalistic auditory scenes

**DOI:** 10.1371/journal.pcbi.1014775

**Published:** 2026-09-21

**Authors:** Richard McWalter, Christian Lorenzi

**Affiliations:** Laboratoire des Systèmes Perceptifs, Département d’Études Cognitives, École Normale Supérieure, PSL University, CNRS, Paris, France; University of California at Berkeley, UNITED STATES OF AMERICA

## Abstract

Natural environments are filled with a diverse array of sound sources, from bird vocalizations and insect stridulations to waterfalls and wind noise. The sounds often co-occur, arriving at the ear of human listeners as a mixture of the different sources. The auditory system is tasked with segregating the individual sound sources of the mixture into meaningful representations. In contrast with human auditory perception in urban environments, which include speech and music, little is known about our capacity to segregate mixtures common in natural environments. Here, we report on the capacities of the human auditory system when presented with mixtures of bird vocalizations – that is, scenes common in natural environments – by systematically varying the number of vocalizations from the same species and from different species. We found that humans could discriminate mixtures but only up until around 8 individuals of the same species beyond which mixtures may rely on a time-averaged summary statistic. Furthermore, humans appear to segregate mixtures of different species based on the disparities in their summary statistics. The results suggest that humans analyse natural auditory scenes using the same segregation mechanisms as for sounds produced by humans and other geophysical sounds.

## Author summary

Walk through the forest and you are immersed in a rich acoustic environment – the singing of birds overhead, the sound of wind through leaves, the chirps of insects in the brush. Making sense of these complex sound environments requires the auditory system to organize multiple simultaneous sounds into meaningful representations. Although the details of this organization have been studied in depth for speech and music sounds, less is known about the auditory processing of nature sounds, such as mixtures of different bird songs. Using a combination of behavioral experiments and computational modeling, we examined the limits of the human auditory processing of naturalistic scenes corresponding to mixtures of bird vocalizations and choruses. Human listeners appear to balance the processing of biodiversity, both in terms of species abundance and richness, using a summary statistic representation for large choruses and segregating species mixtures by the disparities in their bird song sound statistics. These findings offer insights into the auditory capacities of humans in natural environments.

## Introduction

De-mixing sound sources is a problem that the human auditory system must continuously solve, in which the sounds in the local environment arrive at the ear as a mixture of different sources. For instance, in natural parks, birds can be heard singing simultaneously with gusts of wind whistling through the trees, or the rustling of small mammals in the leaves simultaneously with the chirps of cicadas high above in the tree canopy (Fig 1A). The auditory system processes the incoming sound mixture by segmenting, integrating and segregating the sounds into meaningful elements, often referred to as auditory scene analysis [1,2].

**Fig 1:**
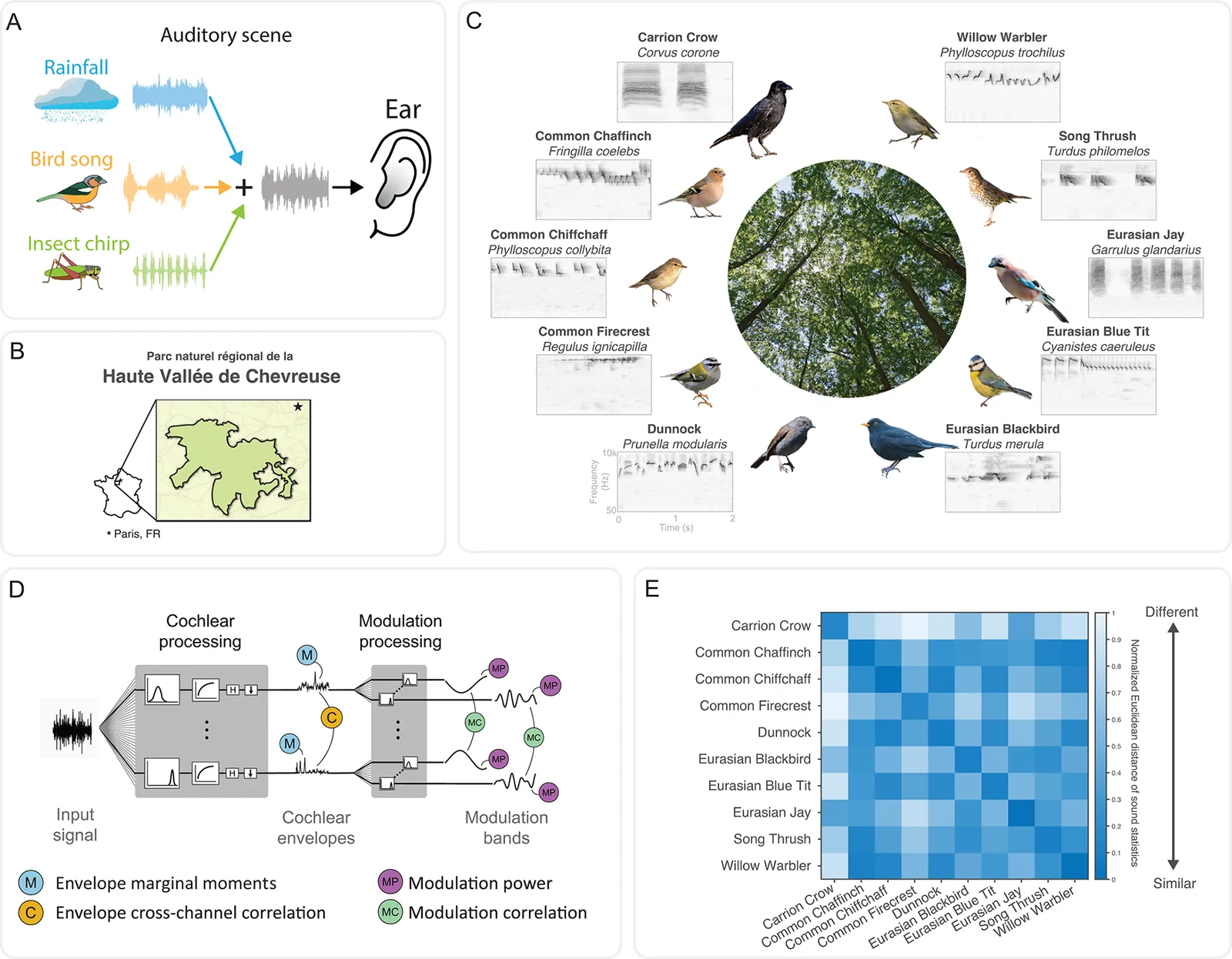
Auditory scenes for bird sounds common in natural parks and the similarity of their auditory texture summary statistics. **(A)** Sound environment can be composed of multiple, concurrent sources that all arrive at the ear as a single waveform. In this example, the sound sources are rainfall, a bird song and insect chirps that sum together in the air, arriving at the ear as a mixture of the three sound sources. **(B)** Sounds collected from Parc naturel régional de la Haute Vallée de Chevreuse, a mixed deciduous forest located approximately 30km southwest of Paris, France, in a temperate region. The park is inhabited by bird species including the Common Chaffinch, Eurasian Jay and Song Thrush. **(C)** 10 species of birds used in the experiments are shown along with example cochleagrams (time-frequency plots). See S1 Fig for additional cochleagram plot examples. The center image shows a photograph of Parc Chevreuse. See Photograph Citations section for source acknowledgements. **(D)** Auditory texture model. Statistics are measured from an auditory model capturing the tuning properties of the peripheral and subcortical auditory system in three stages. The statistics measured from this model include marginal moments and pairwise correlations at different stages. **(E)** Difference in sound statistics of bird songs from the 10 species measured by the auditory texture model: diagonal shows the within-species differences and off-diagonal shows the across-species differences. Note that differences represented off-diagonal (between species) are generally larger (lighter blue) than the differences represented by diagonal (darker blue). Overall, the figure illustrates the across-species differences tend to be larger than the within-species differences. However, the variation in blue shade suggests that the vocalizations for some species are more similar than others. See S2 Fig for individual bird recording difference matrix.

Auditory scene analysis has primarily been studied using artificial stimuli, such as tones and noises, as well as speech and music sounds [2–9]. The latter are sounds characteristic of domestic and urban scenes as in the case of listening to someone talking in a crowded bar. Recent work has explored the perception of sounds common in natural settings with subjective numerosity judgments and magnitude scales or free-sorting tasks [10,11]. In this study, we aimed to characterize the auditory perception of collections of sounds common in natural environments. These sounds span both within and across bird species mixtures as well as background sounds from non-bird species and environmental sound sources. The use of natural sounds enabled the design of novel experiments revealing aspects of human auditory scene analysis that, to the best of our knowledge, have never been comprehensively studied from a psychophysical perspective using objective discrimination paradigms.

Natural environments are acoustically rich and diverse. They often include biological sounds, such as bird vocalizations and insect stridulations, and geophysical sounds, such as wind, rain, thunder or water waves [12]. The number and type of biological sound sources in a given habitat is often described in terms of “biodiversity”, a construct encapsulating the species richness, or the number of different species, and the species abundance, or the number of individuals from the same species [13]. Perceiving levels of biodiversity is crucial to many living beings as it provides vital information about the status of the habitat, and plays an active role in ecological processes, such as habitat selection [14–17]. Geophysical sounds also provide crucial information about the surrounding environment, but they may simultaneously impact the level of perceived biodiversity through masking and interference effects [18]. Traditionally, the biological and geophysical sounds have been captured by placing microphones in natural settings to monitor the environment through acoustic recordings [19,20]. More recently, computational methods have been developed to assess the level of biodiversity from soundscape field recordings, a research area embedded in ecoacoustics [15,21]. However, when biological and geophysical sound sources arrive at the microphone simultaneously, accurately assessing biodiversity becomes more difficult [22].

There is diversity in the acoustic signals produced by birds. Vocalizations from birds of the same species may differ in their spectral and temporal content and individuals may alter their vocal behavior relative to the biological and geophysical sounds in the surrounding environment [23–26]. In addition, the geophysical sounds, such as rain, can have an impact on the vocalizations produced by individual birds [26–28]. The variations in the spectral and temporal characteristics of bird vocalizations are larger across-species than within-species [24], and are a consequence of differences in size and shape of the syrinx as well as learned traits [29]. The differences in spectral and temporal characteristics have also been identified across-species for insects and amphibians, and are related to environmental constraints put upon the species culminating in evolutionary theories of acoustic adaptation [30–34] and acoustic niche [35]. In some cases, acoustic signals produced by vocalizations from different species may limit the overlap in their spectral and temporal features, even when they occur concurrently. The result is a rich acoustic environment, with species vocalizing in unique ways that span a wide range of spectral and temporal modulations [36,37].

The perception and organization of auditory scenes has been linked to several sound features. For instance, sounds with a common onset tend to be grouped together (see [2,4,6,38,39] for a review). Beyond these traditional cues, amplitude and frequency modulations have been implicated in grouping or segregating different sound sources, relating natural sound regularities or statistics to auditory scene analysis [40–42]. One category of sound where statistics are thought to be crucial in their recognition and perception is sound textures [43–45]. Sound textures are a category of sound that are common in natural scenes, and often make up the background sounds of an auditory scene. Sound textures are produced by a superposition of many similar acoustic events, such as those from a flock of birds or a swarm of insects chirping, as well as sounds originating from rainfall, streams and fires [46]. However, unlike the sound of a single event, such as a branch breaking, or of the complex temporal sequences of a single bird singing, a texture is defined by properties that remain constant over time [47]. Sound textures are ubiquitous in natural parks and texture perception could be a cornerstone of auditory perception in natural environments. An important aspect of auditory discrimination of textures is its reliance on a temporal integration process that keeps a small set of time-averaged summary statistics, such as modulation power and correlation of temporal envelopes across frequency channels, while discarding fine acoustic details [43,45,47]. Consequently, temporal integration may occur for many natural sounds, including wind gusts, rainfall and potentially large bird choruses, a large number of simultaneous bird vocalizations, as has been shown with other temporally dense sound sources, such as multi-talker babble [47].

How the auditory system processes natural scenes remains relatively unstudied. We hypothesized that listeners would be able to discriminate differences in the size of a bird chorus, and that when a mixture of bird species is presented, listeners would perceptually group or segregate the species. But under what conditions are sound sources perceptually grouped or segregated and would there be limits on the discriminability of chorus size? To test the hypotheses, we ran a series of psychophysical listening experiments to evaluate the capacities of the human auditory system to discriminate acoustic mixtures that varied in both bird species abundance and species richness. We found evidence for listeners using different auditory mechanisms depending on the species abundance, where large bird choruses may be represented with time-averaged summary statistics. In experiments using mixtures of different species, we found that the species statistics can either promote or hinder sound segregation. We supported our behavioral findings with auditory models that provided insight into the nature of auditory grouping and streaming of naturalistic auditory scenes.

## Results

We referenced real-world sound recordings of bird vocalizations conducted in a natural park when designing our experiments. These recordings were conducted in a single habitat with the aim of capturing the balance between bird vocalizations and the surrounding environment [21,30,35]. Analysis of the recordings highlighted two significant, if not obvious, conditions relating to species abundance and richness. The first was that birds from a single species can vary in number. The second was that the diversity of species can vary in a recording. As indicated above, both species abundance and species richness are key elements of biodiversity assessment [13]. These observations motivated our two main research questions related to natural sound scene perception: (i) can listeners discriminate bird choruses of different sizes from the same species, and (ii) under what conditions do listeners perceptually group or segregate mixtures of different bird species?

To investigate the perception of natural scenes that varied in bird species abundance and richness, we opted to work with real-world sound recordings collected in the field. We began by identifying bird species that inhabit a region south-west of Paris, France called the Parc naturel régional de la Haute Vallée de Chevreuse (Fig 1B), a temperate deciduous forest that has previously been characterized in ecoacoustic studies [48,49]. Ten bird species were selected for their prevalence in the region as well as the diversity in the acoustic properties of their bird songs and calls (Fig 1C). Ten reference recordings of each individual bird species were identified and used to create an audio database of bird vocalizations drawn from the Cornell Lab of Ornithology | Macaulay Library, consisting of at least 80 individual birds per species. This audio database of bird vocalizations was used in the experiments.

Unlike artificial stimuli or material recorded in controlled sound laboratories, real-world field recordings may contain extraneous noise and vary in recording quality as well as the distance of the sound source to the microphone. In addition, the crowd-sourced nature of the recordings meant that the equipment setup likely varied across recordings. To mitigate these issues and to approach the sound quality of lab recorded material, we selected only short 3 second excerpts from longer recordings that preserved acoustic homogeneity across individuals from the same species and acoustic diversity across species (S1 Fig).

To quantify the acoustic diversity of bird species vocalizations in our database, we measured the summary statistics using an auditory texture model (Fig 1D). To account for the difference in scale of the texture statistics classes, we z-scored across sounds. The distance was then computed and normalized by the largest distance found within our database. Fig 1E highlights the within- and between-species acoustic disparities that are potentially audible for humans for the present bird sound dataset (for individual sounds, see S2 Fig). In addition, a boosted decision-tree classifier identified all 10 species from their texture statistics with at least 90% cross-validated accuracy (see S3 Fig). The auditory texture statistical analysis and high classification accuracy suggest there is similarity for the within-species recordings and differences when comparing across-species recordings.

### Experiment 1: Auditory discrimination of species abundance

Real-world sound recordings conducted in natural spaces can often contain more than one individual from the same species. This is referred to as species abundance and is identified as a key metric in biodiversity assessment. However, since the acoustic properties of individuals from the same species can be similar in time and frequency, it could be difficult for human listeners to accurately assess the number of individuals. What are the capacities for discriminating such differences in chorus size for human listeners? For example, can listeners discriminate a chorus of 1 bird versus 2 birds singing?

To understand the capacities of human listeners to discriminate differences in species abundance, we designed a discrimination task based on a forced-choice, oddity paradigm [50] with bird choruses differing in number of individuals from the same species. We generated mixtures of bird vocalizations by superimposing sound waveforms of real-world bird vocalizations from the same species varying in number from 1 to 32, which corresponds to the chorus size. We presented listeners with three observation intervals, where one of the intervals was different in chorus size (the target interval) from the other two (the standard intervals). Listeners were asked to judge which interval was different from the other two (Fig 2A). We conducted 2 versions of the experiment, the first version where the 2 standard intervals were exemplars sampled from the same recording (and thus, from the same individual; Experiment 1a, circles), and the second version where the 2 standard intervals were exemplars sampled from different recordings (and thus, from different individuals of the same species; Experiment 1b, squares).

**Fig 2:**
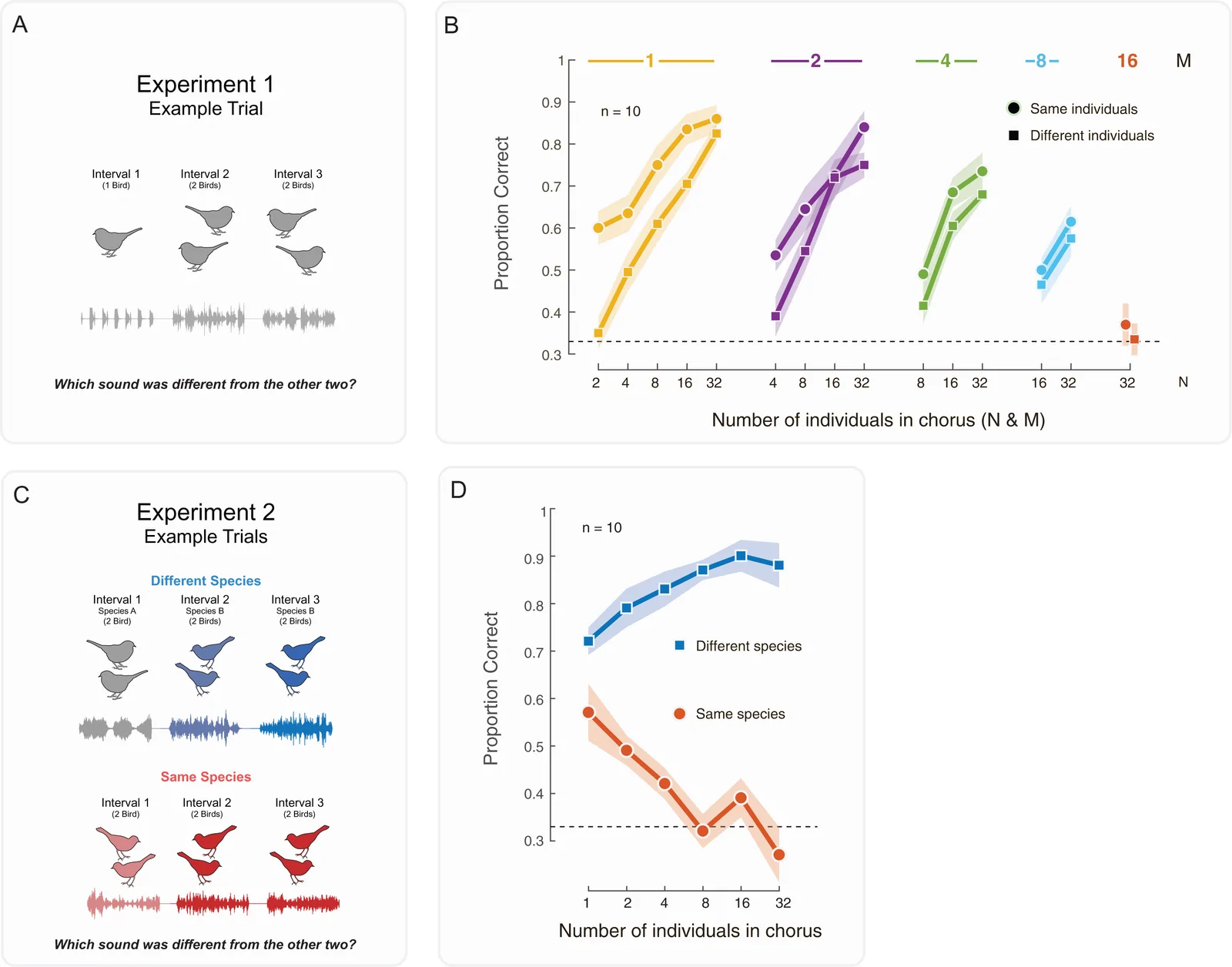
Experiment 1a/b: abundance discrimination improves with difference in chorus size, and Experiment 2: Divergent performance of different (across) and same (within) species discrimination. **(A)** Experiment 1: abundance discrimination task. On each trial, listeners heard three 2s intervals. The two standard intervals were generated from one chorus size (N) and the target interval was generated from a different chorus size (M). Experiment 1a had the two standard intervals sampled from the same individuals (circles) and Experiment 1b had the two standard intervals sampled from different individuals (squares). Listeners were asked to identify the sound that was different from the other two. **(B)** Experiment 1 results. Average proportion correct is plotted as a function of chorus size comparison (N compared to M). Shaded area shows standard error of the mean. **(C)** Experiment 2: species discrimination task. On each trial, listeners heard three 2s intervals. The chorus size was the same for all three intervals. For the same species condition (red circles), the standard and target chorus were composed of individuals from the same species. For the different species condition (blue squares), the standard and target chorus were composed of individuals from different species. **(D)** Experiment 2 results. Average proportion correct is plotted as a function of chorus size. Error bars show standard error of the mean. Number of participants (n) is shown in the top-left of panels B and D.

Chorus size discrimination performance improved when the difference in abundance increased (Fig 2B). Listeners performed well above chance level when discriminating chorus size for target or standard intervals less than 16. For Experiment 1a, performance improved significantly with chorus size for M = 1 (F_4,36_ = 13.9, p < 0.001, η^2^_p_ = 0.61), M = 2 (F_3,27_ = 13.8, p < 0.001, η^2^_p_ = 0.61), M = 4 (F_2,18_ = 18.3, p < 0.001, η^2^_p_ = 0.67), and for M = 8 (F_1,9_ = 8.19, p = 0.019, η^2^_p_ = 0.48). For Experiment 1b, performance improved significantly with chorus size for M = 1 (F_4,36_ = 43.8, p < 0.001, η^2^_p_ = 0.83), M = 2 (F_3,27_ = 22.7, p < 0.001, η^2^_p_ = 0.72), M = 4 (F_2,18_ = 9.05, p = 0.002, η^2^_p_ = 0.50), though not significantly for M = 8 (F_1,9_ = 2.86, p = 0.13, η^2^_p_ = 0.24). However, when the chorus size of the target and standard were equal to or greater than 16, listeners’ performance was at chance (Exp 1a: p = 0.48, and Exp 1b: p = 0.67). In addition, listeners’ discrimination performance was higher when the 2 standard intervals were excerpts from the same recording and lower when the 2 standard intervals were excerpts from different recordings, which was more pronounced when the chorus size was smaller.

The results suggest listeners can discriminate the chorus size when the standard or target are small. However, listeners appear to perform poorly when the chorus size of the standard and the target were large. Listeners’ performance was overall better when the 2 standard intervals were different excerpts of the same recording, suggesting listeners may retain details of the specific individual bird vocalizations and be able to discriminate one individual from another. However, other factors such as recording environment and technique may contribute to the difference in performance. Notably, listeners’ performance was at chance when the chorus size of the standard and the target was large, suggesting listeners may reach an upper limit on discriminability when comparing chorus sizes equal to or greater than 16 individuals.

### Experiment 2: Auditory discrimination across and within-species

The acoustic signals produced by birds can vary across individuals from different species, but more subtly between individuals from the same species [24]. These acoustic disparities may be perceived by our human participants and are illustrated for the present set of recordings in Fig 1E. How sensitive are human listeners to differences within and across bird species? To answer this question, we designed two experiments (Fig 2C). The first was a species discrimination experiment where the two standard intervals consisted of vocalizations produced by the same species and the target interval consisted of vocalizations of a different species. The second was a same species discrimination experiment where the 2 standard intervals were excerpts from the same individual and species and the target was an excerpt from a different individual from the same species. For both experiments, the target and the standard were the same chorus size, and we varied the chorus size from N = M = 1–32.

For the species discrimination experiment, performance increased with chorus size (F_5,45_ = 6.06, p < 0.001, η^2^_p_ = 0.40) but plateaued at a chorus size of 8 or 16 (Fig 2D, blue squares). For the same species discrimination experiment, performance decreased with chorus size (F_5,45_ = 3.66, p = 0.007, η^2^_p_ = 0.29), reaching chance performance for a chorus size of 8 and above (Fig 2D, red circles). Consistent with the species distance measures shown in Fig 1E, the results suggest the perceptual differences across species are greater than those within-species.

### Experiment 3: Auditory cues used for discrimination of species abundance

Do the auditory cues differ when listening to small versus large bird choruses? To address this question, we designed an experiment where we probed listeners' ability to discriminate bird chorus exemplars at two durations (Fig 3A). We generated bird choruses varying in number by superimposing recordings of different bird vocalizations from the same species. Listeners were presented with two excerpts taken from the same signal, that is two different clips of the same group of birds singing, one of which was presented twice. Listeners were asked to judge which of the three sounds was different from the other two. The bird chorus size was 1, 8 or 32. The duration of the intervals was either short (50ms) or long (2s).

**Fig 3:**
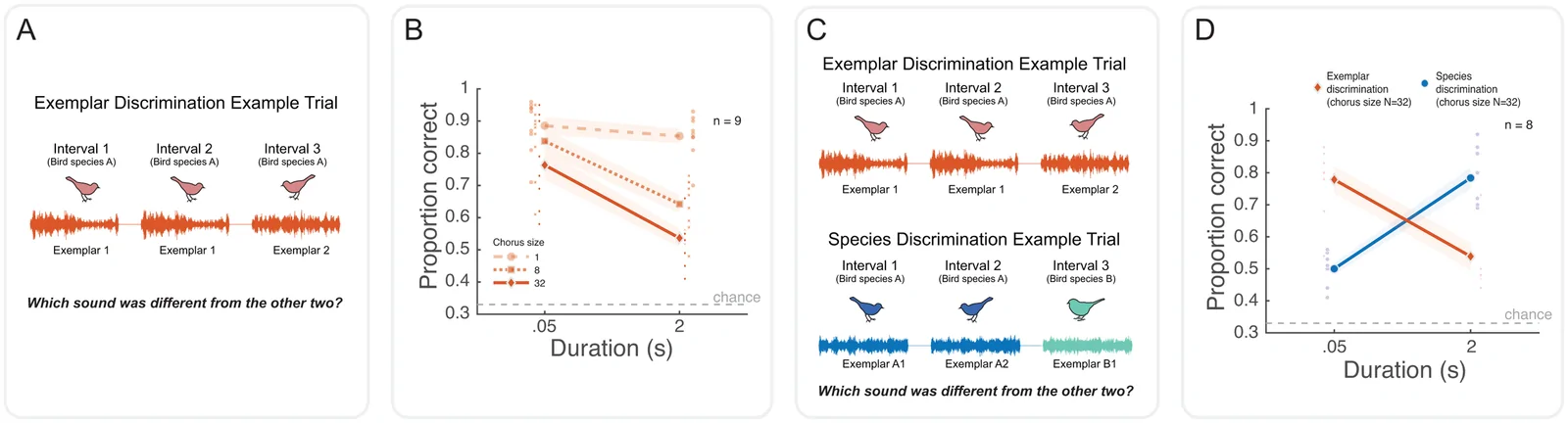
Experiment 3a and 3b: Exemplar discrimination performance decreased as a function of duration and chorus size. **(A)** Experiment 3: abundance exemplar discrimination oddity task. On each trial, listeners heard three intervals. The chorus size on each trial was either 1 (light red circles), 8 (medium red squares) or 32 (dark red diamonds) birds for two durations, 50ms and 2s. The two *standard* intervals were created from the same excerpts, and thus had the same waveform, and the *target* interval was created from a different excerpt from the same species and therefore had a different waveform. Listeners were asked to identify the sound that was different from the other two. **(B)** Experiment 3a results. Average proportion correct is plotted as a function of duration for each chorus size. Small markers show individual participant results. Error bars show standard error of the mean. **(C)** Experiment 3b species (blue square) and exemplar (red diamond) discrimination task. On each trial, listeners heard three intervals. The chorus size was 32 birds and the duration was either 50ms or 2s. In the species discrimination task, two of the intervals were created from the same species but different excerpts and the target interval was created from a different species. The exemplar discrimination task was the same as in Experiment 3a. **(D)** Experiment 3b results. Average proportion correct is plotted as a function of duration. Error bars show standard error of the mean. Number of participants (n) is shown in the top-right of panels B and D.

The results showed that the short duration exemplars were highly discriminable for all bird chorus sizes (Fig 3B). In contrast, performance for the long duration varied across chorus sizes, yielding an interaction between duration and the number of birds in the chorus (F_2,16_ = 94.3, p < 0.001, η^2^_p_ = 0.92). For a single bird singing, or chorus size of 1, performance was comparable across the short and long durations (t = 1.01, p = 0.34). However, for a chorus size of 8 and 32, performance decreased with duration, with a larger decrement for the largest chorus size (t = 5.37, p < 0.001 and t = 4.18, p = 0.003, respectively).

As a further test, we examined the discrimination of large choruses for two complementary tasks using the oddity paradigm: 1) exemplar discrimination, and 2) species discrimination (Fig 3C). For the exemplar discrimination task, we replicated Experiment 3a for a chorus size of 32 birds. For the species discrimination experiment, listeners were presented with two choruses created from the same species (different clips of the same group of birds singing), and one chorus created from a different species, again with a chorus size of 32 birds. Listeners were asked to judge which sound interval was different from the other two. The duration of the intervals was either short (50ms) or long (2s).

The results, presented in Fig 3D, showed performance decreased with duration for the exemplar discrimination task, comparable to the results of Experiment 3a for the chorus size of 32 vocalizations (t = 4.86, p = 0.002). In contrast, performance increased with duration for the species discrimination task (t = 8.12, p < 0.001). This dichotomy between within-species exemplar discrimination task and across-species discrimination task exposes limitations of the auditory system when listening to sounds over different time scales.

Taken together, experiments 1–3 characterized the ability of human listeners to discriminate sounds that varied in species abundance, species richness and chorus size. Listeners were able to discriminate species abundance for small chorus sizes but less so for larger chorus sizes (Experiment 1). Listeners were better at discriminating different species compared to the same species (Experiment 2). Lastly, in Experiment 3, listeners appeared to retain the temporal details of small choruses, but lost access to these temporal details for large choruses, consistent with previous findings where auditory perception of more dense sounds is limited to time-averaged summary statistics resulting from temporal integration of the acoustic signal [47].

### Experiment 4: Auditory discrimination of species richness

Species richness depends on the biome, moment of the day, and season, from an acoustically sparse chorus composed of just a few birds occurring in the afternoon in a semi-arid desert to the acoustically dense dawn chorus in a coniferous forest composed of many species and individuals. We were interested in characterizing human listeners' ability to discriminate sounds composed of acoustic mixtures of different species versus those composed of a single species, where the sounds spanned a range of chorus sizes. To investigate the perception of acoustic mixtures of different bird species, we designed a three-interval experiment based on the oddity paradigm with three conditions: (1) where listeners were discriminating different species, (2) where listeners were discriminating mixtures of two species, and (3) where listeners were discriminating the same species (Fig 4A). We reasoned that if listeners were to perceptually segregate the mixture conditions into two auditory streams, we would observe comparable performance for the different species and mixture of species conditions, as the listeners would have access to the auditory features of each species individually. Alternatively, if listeners perceptually group or “fuse” the mixture condition, performance would tend towards that of the same species condition, as listeners would estimate the features from a combination of the species.

**Fig 4:**
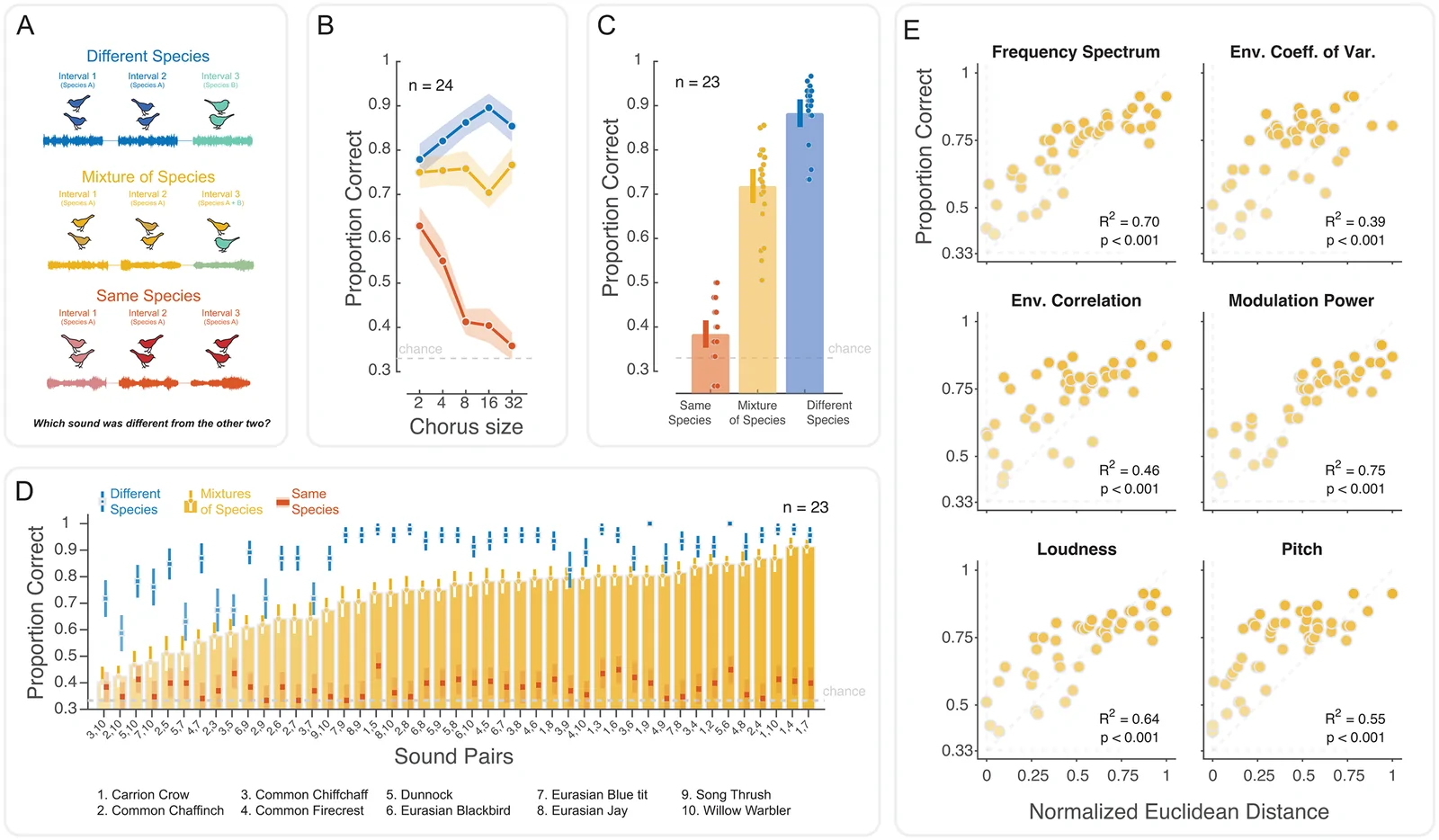
Experiment 4a and 4b: Discrimination of species mixtures correlates with several cues commonly associated with auditory stream segregation. **(A)** Experiment 4a: species mixture discrimination task. On each trial, listeners heard three 2s intervals. The chorus size on each trial varied from 2 to 32. The different species discrimination condition consisted of two standard intervals created from one species and a target interval created from a different species, shown in blue. The mixture discrimination condition consisted of two standard intervals created from the same species (species A) and a target interval created from a mixture of 2 different species, shown in yellow. The mixture interval was composed of half individuals from species A and the other half of individuals from species B. The same species discrimination condition consisted of two standard intervals created from the individuals from the same species, but different excerpts, and a target interval created from the same species but different individuals, shown in red. Listeners were asked to identify the sound that was different from the other two. **(B)** Experiment 4a results. The average proportion correct is plotted as a function of chorus size. Error bars show standard error of the mean. **(C & D)** Experiment 4b results. On each trial, listeners heard three 2s intervals and the chorus size was set to 16 birds. The experiment was different from Experiment 4a in that every species mixture combination was tested, 10 species and 45 combinations. For panel C, the plot shows the mean proportion correct averaged across all species mixture combinations along with individual participant means. For panel D, the plot shows the proportion correct for every set of species mixture pairs. The red marks are the average of the same species condition for the species pair. The error bars show standard error of the mean. **(E)** Six acoustic cues (stimulus properties) commonly associated with auditory grouping. The correlation value is the R-squared (R^2^) between the acoustic cue Euclidean distance of the sound pairs and proportion correct for the mixture condition. Number of participants (n) is shown at the top of panels B, C and D.

The results of Experiment 4a, presented in Fig 4B, show that listeners’ performance was generally high when discriminating sound from the different species condition, increasing slightly with the chorus size (F_4,92_ = 5.15, p < 0.001, η^2^_p_ = 0.18). Listeners’ performance for the mixture condition was comparable to that measured in the different species condition for a chorus size of 2 birds but lower in performance for chorus sizes greater than 2, with performance relatively constant across chorus sizes (F_4,92_ = 0.75, p = 0.56, η^2^_p_ = 0.03). Lastly, listeners’ performance tended to be low when discriminating sound from the same species condition, decreasing towards chance with increasing chorus size (F_4,92_ = 9.39, p < 0.001, η^2^_p_ = 0.29).

The decrease in performance of the mixture condition relative to the different species condition suggests listeners do not perfectly segregate the bird species mixtures except for mixtures of small choruses composed of two birds. But is the performance dependent on the type of bird species selected for the mixture? To address this question, we repeated Experiment 4a with all possible combinations of the 10 species for a chorus size of 16 birds. If the performance were to be similar for all species mixture pairs, the acoustic properties would have little impact in promoting perceptual grouping or segregating the mixture. But, if there were a difference in performance across species mixture pairs, perhaps the underlying features that promote segregation could be identified.

The results of Experiment 4b are shown in Fig 4C and 4D. The average result across the mixture pairs replicates that of Experiment 4a for the chorus size of 16. The same species condition performance was low, whereas the different species condition was high (Fig 4C). We found that performance varied across the species mixtures, with some species mixtures approaching chance performance and other species mixtures approaching ceiling performance (Fig 4D). Performance was comparatively high for the different species discrimination task and low for the same species discrimination task.

What might cause the mixture condition to vary across species pairs? As a first assay, we analyzed the similarity between sound pairs for a variety of features commonly associated with auditory grouping [6,8,51,52]. These features include the frequency spectrum, amplitude-modulation power, broadband temporal envelope variance, correlation of temporal envelope cues across frequency channels, pitch and loudness. To obtain an estimate of the difference between sound pairs for each feature class, we measured the Euclidean distance between auditory grouping features. The Euclidean distance was normalized between 0 and 1, where 0 was the minimum distance and 1 was the maximum distance for each auditory streaming feature. We compared the distance between sound pairs and human discrimination performance for the bird chorus mixture by measuring the Pearson correlation coefficient (Fig 4E). The correlation values varied across sound feature classes, with the highest correlation value for the modulation power and frequency spectrum classes and the lowest for the envelope coefficient of variation. The analysis suggested that listeners may be grouping or segregating bird vocalizations based primarily on disparities in frequency spectrum and modulation power when performing the discrimination task, which can account for some of the variance in the results across species pairs.

### Auditory Model: Do listeners discriminate sound mixtures using summary statistics?

The results of Experiment 4 suggest that listeners may be streaming the acoustic mixtures to perform the task. However, an alternative hypothesis is that listeners may simply estimate global properties of the sound stimulus to perform the task. To investigate this hypothesis, we developed seven observer models that operated on our experimental stimuli and were run on the same experimental tasks as human listeners. The observer models were based on the six auditory grouping features shown in Fig 4E, in addition to an observer model based on the auditory texture model (Fig 1D), which has been shown to capture perceptually relevant features of natural auditory scenes composed of animal choruses and geophysical sounds [43].

The same general framework for the observer model was employed for all streaming features and the auditory texture model (Fig 5A). The observer models operated on the stimuli used in the behavioral experiment. On each trial, the model accepted the three input sound intervals, measured the parameters for each of the sounds, computed the Euclidean distance between the three intervals, and finally selected the interval that was most different relative to the other two intervals. This approach could be seen as conceptually similar to the optimal strategy a listener might use on a given trial for the present forced-choice, oddity tasks [50].

**Fig 5:**
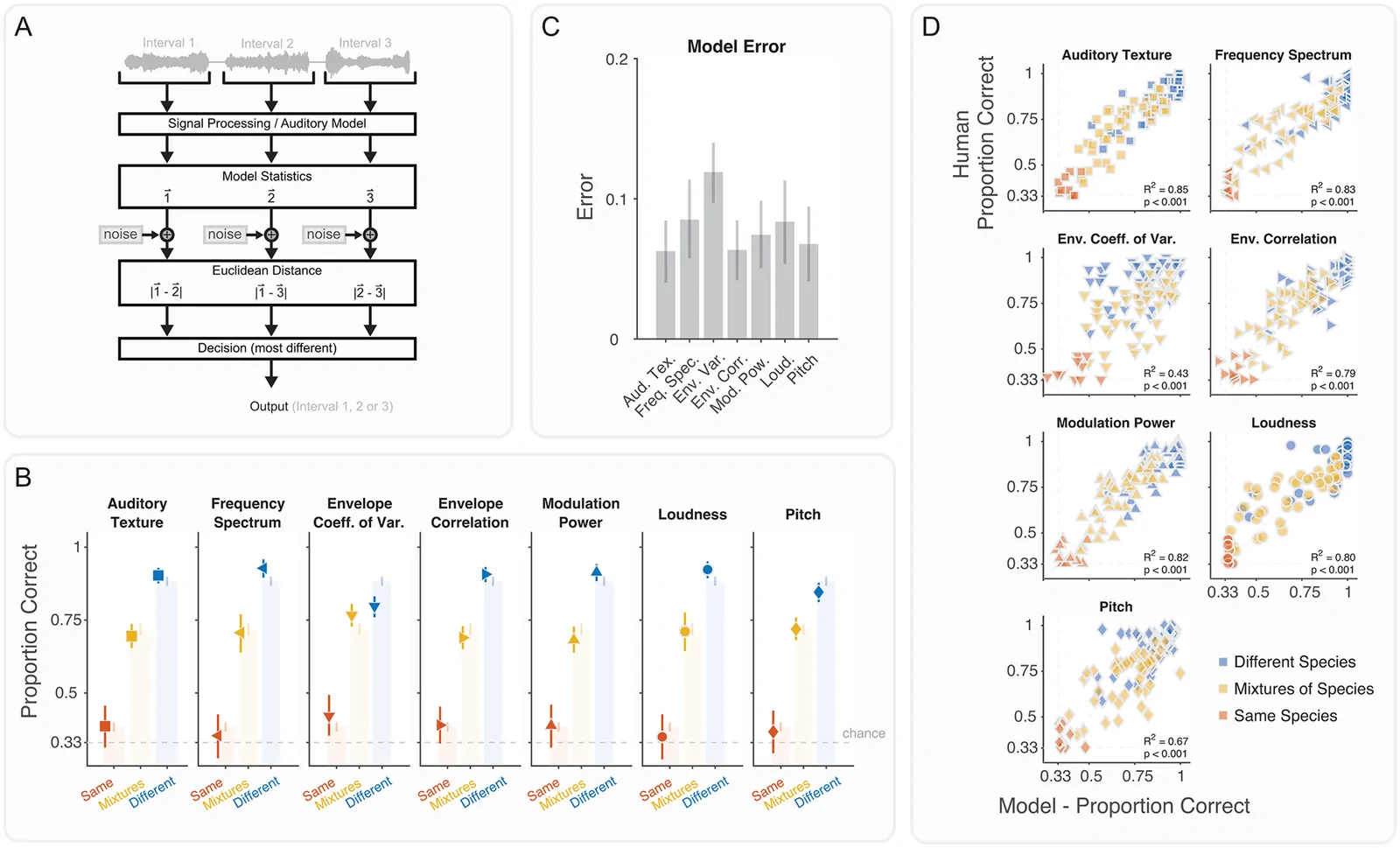
Observer models revealed many acoustic cues correlate well with human performance for different, mixture and same species conditions. Seven observer model variants were implemented based on six acoustic cues shown in Fig 4E in addition to an auditory texture observer model that incorporates several acoustic cues. The model structure was the same for all acoustic cue types. **(A)** Observer model. The model averaged statistics within a rectangular window extending from the endpoints of the three intervals, added a Gaussian internal noise with a fixed and unique variance to each statistic (internal noise aimed to introduce randomness in the model behavior). The model assumed a Euclidean metric for each acoustic cue. **(B)** Observer model output. Results of each observer model are plotted as a proportion correct and overlaid on the human results from Experiment 4b (Fig 4C). Markers show model results and bars show human results. Model error bars show 95% confidence intervals; human error bars show standard error of the mean across participants. **(C)** The plot shows the error between the human and model summed across the three experimental conditions. The error bars show ±1 SD. **(D)** Correlation between human and observer model results. The subplots show correlation for the different species (blue), mixture of species (yellow) and same species (red) discrimination conditions for the 7 observer model types. Each subplot shows the R-squared (R^2^) value computed via the Pearson correlation coefficient across all experiment conditions.

All versions of the observer model yielded results that differ across the three experiment conditions: (1) different species, (2) species mixtures, and (3) same species (Fig 5B-5C). Most of the observer models had high performance for the different species condition (blue), low performance for the same species condition (red), and intermediate performance for the species mixture condition (yellow). Generally, the results suggest that listeners could use a global estimate of the scene size, and do not necessarily need to stream or segregate the sound mixture. Although all models showed differences in performance across the three conditions, the auditory texture observer model appeared to most closely match human performance (Fig 5C, leftmost bar), though it was not statistically different from several other models. In addition, we assessed the correlation between the human and model performance (Fig 5D). Correlation values varied across models, with the highest correlation values found for the Auditory Texture Model. However, similarly high correlation was also found for individual auditory features, such as Frequency Spectrum, Envelope Correlation, and Modulation Power. An ablation analysis of the Auditory Texture Model suggested that the Modulation Power statistics class has an outsized contribution to the model discrimination performance (see S4 Fig). Overall, the Auditory Texture Model was not significantly better than the Frequency Spectrum, Loudness, Pitch, Modulation Power or Envelope Correlation observer models, but appeared to qualitatively replicate the discrimination performance of human listeners across the three experimental conditions.

### Experiment 5: Do listeners group or segregate mixtures of species?

Next, we explored the streaming capacities of human listeners with a more nuanced experiment design. Here, we turned to the exemplar discrimination task from Experiment 3, which revealed that listeners’ performance decreased with increasing bird chorus size (Fig 6A). Specifically, for the 2 second condition, listeners’ performance was highest for a single bird vocalization, lower for a chorus size of 8 birds, and lower still for a chorus size of 32 birds. In the present task, this decrease in performance with chorus size was used as an indirect measure of auditory grouping or segregation: performance for mixtures should decrease if the mixture species are grouped together and remain stable if the mixture species are segregated for a given chorus size. To make the hypothesis explicit, we could take the case of two species, each represented by a chorus size of 8 birds. If the species are grouped perceptually, performance should be at or near that of a chorus with a size of 8 + 8 = 16 birds, whereas if the two species are perceptually segregated, performance should be at or near that observed for a chorus size of 8 birds. In the segregated case, listeners should be able to perform the discrimination on either chorus of 8 birds, even though they are presented simultaneously.

**Fig 6:**
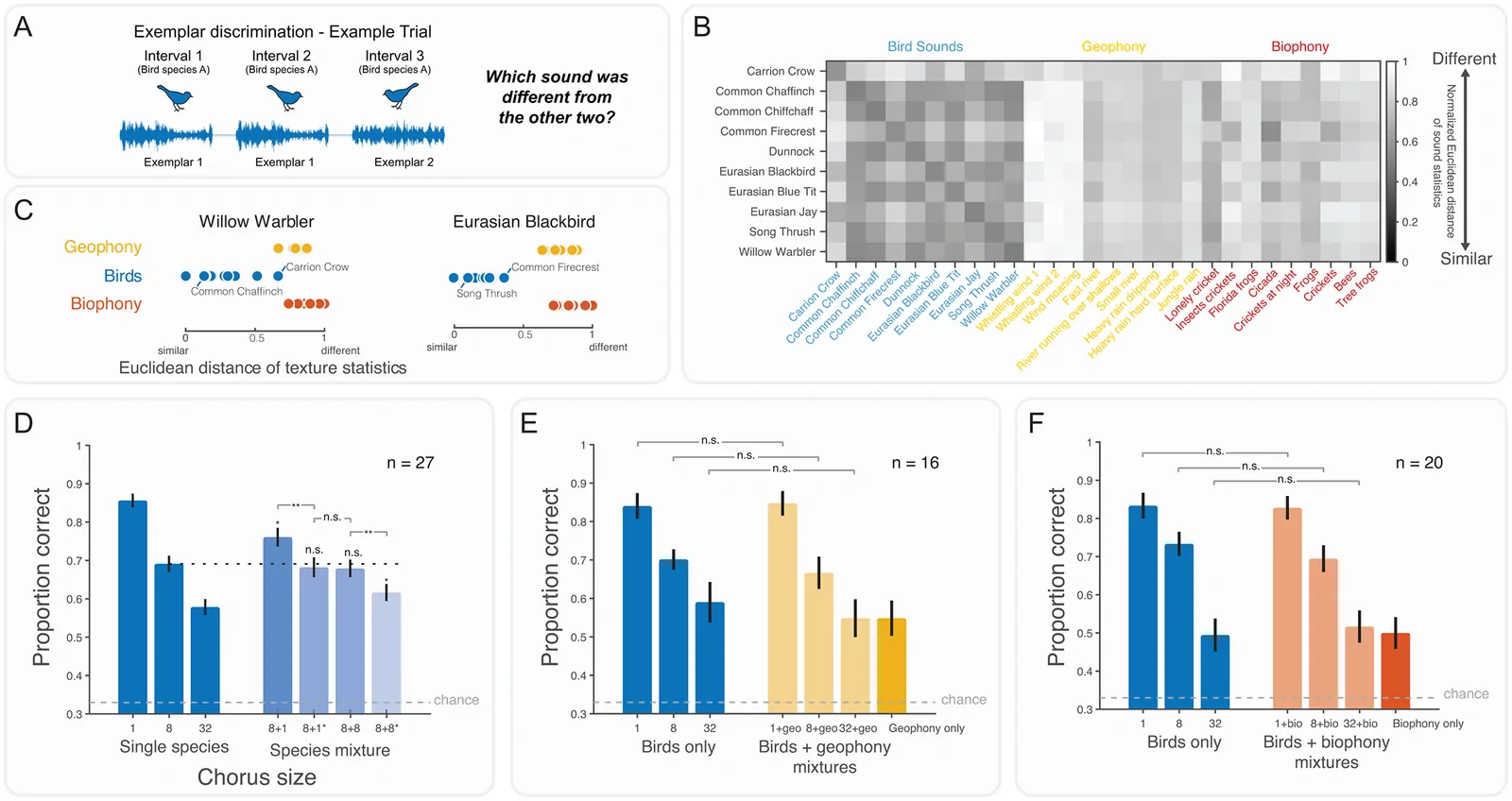
Experiment 5 and 6a/b: Auditory segregation of species mixtures may be related to the disparities of their summary statistics. **(A)** Example trial for exemplar discrimination task. Listeners heard three 2-second intervals. Two intervals were identical sounds (exactly the same waveform) and the other interval was a different exemplar from the same source(s). Listeners were asked to report which sound was different. **(B)** Euclidean distance of texture statistics between bird species as well as geophysical (e.g., rain, river, wind) and biotic (e.g., frogs, insects) sounds, referred to as geophony and biophony respectively. The statistics of the sound sources were computed from the Auditory Texture Model. Distance of the statistics was computed relative to a reference species. **(C)** Two reference species examples are shown, one for the Willow Warbler and the second for the Eurasian Blackbird. For Experiment 5, the most similar bird species to the Willow Warbler was the Common Chaffinch (8 + 1* and 8 + 8* conditions) and the most different bird species was the Carrion Crow (8 + 1 and 8 + 8 conditions). The bird species most similar to the Eurasian Blackbird was the Song Thrush and the most different was the Common Firecrest. The biophony and geophony background sounds used in Experiment 6 had statistics that were overall more different than the bird species. See Table 2 for all bird species pairs and Table 4 for the geophony and biophony background sounds. **(D)** Experiment 5 results. The average proportion correct is plotted as a function of chorus size. Error bars show standard error of the mean. Chorus size with a single number corresponds to single species conditions. Chorus size with 2 numbers corresponds to species mixture conditions. Statistical significance from a chorus of 8 is marked by an asterisk (*) and non-significance by the letters n.s., while mixture condition statistical significance is marked by a double asterisk (**) and non-significance by the letters n.s. Statistical analysis was run as repeated measures ANOVA and corrected (Bonferroni) for multiple comparisons (see Table 3). **(E)** Experiment 6a results for background of geophony. The average proportion correct is plotted as a function of chorus size. Error bars show standard error of the mean. Chorus size with background condition shown as “+geo”. Background only condition shown as “Geophony only”. See Table 4 for list of geophysical background sounds. **(F)** Experiment 6b results for the biophony background. The average proportion correct is plotted as a function of chorus size. Error bars show standard error of the mean. Chorus size with background condition shown as “+bio”. See Table 4 for list of biotic background sounds. Background only condition shown as “Biophony only”. Number of participants (n) is shown at the top of panels D, E and F.

Because the extent to which streaming occurs is directly related to the salience of the perceptual difference between sounds [38], we first measured the texture statistics for each of the 10 bird species and calculated the Euclidean distance between the species pairs. We then identified pairs of species that were most similar and the pairs of species that were most different (Fig 6B). For instance, the Willow Warbler is most similar to the Common Chaffinch and most different from the Carrion Crow (Fig 6C). The mixtures were carefully selected so that one condition included species mixtures with pairs that were most similar and another condition with pairs that were most different. There were four mixture conditions. The first two conditions consisted of 1 individual bird vocalization combined with 8 bird vocalizations from a different species, which were created using either the most different (8 + 1) or the most similar (8 + 1*) species pairs. The next two conditions consisted of 8 bird vocalizations combined with 8 bird vocalizations from a different species, which again were created using either the most different (8 + 8) or the most similar (8 + 8*) species pairs. In addition, baseline conditions were replicated from Experiment 3 including a single bird vocalization, 8 bird chorus, and 32 bird chorus.

The results from Experiment 3 showed that exemplar discrimination performance decreased with increasing chorus size when the chorus was composed of recordings from the same species (Fig 6D). The results were replicated here in Experiment 5, where exemplar discrimination performance was high for a single bird vocalization and then decreased for the chorus of 8 and 32 (F_2,52_ = 108.3, p < 0.001, η^2^_p_ = 0.81).

The mixture results for Experiment 5 are shown in Fig 6D. The first mixture condition consisted of a chorus of 8 birds paired with a single bird vocalization with the most different statistics (condition 8 + 1). The results show that performance was significantly higher (difference = 0.070, SE = 0.017, p = 0.004) than the condition with 8 birds only, suggesting listeners can use stimulus properties from the single bird vocalization when performing the discrimination. However, performance does not match that of a single bird vocalization presented in isolation, which may be a consequence of perceptual masking caused by the concurrent chorus of 8 birds. The second condition consisted of a chorus of 8 birds paired with a single bird vocalization with the most similar statistics (condition 8 + 1*). The results show that discrimination performance decreased slightly from a chorus of 8, though not significantly. In the 8 + 1* condition, listeners do not seem to benefit from the single bird vocalization and appear to be hindered by the additional bird vocalization, as indicated by the decrease in discrimination performance. The third condition consisted of an 8-bird chorus paired with an 8-bird chorus with the most different statistics (condition 8 + 8). Listeners’ discrimination performance was similar to that of an 8-bird chorus presented in isolation, suggesting listeners may be able to perform the discrimination with either species group. Lastly, the fourth mixture condition consisted of an 8-bird chorus paired with an 8-bird chorus with the most similar statistics (condition 8 + 8*). The results show a significant decrease (difference = 0.075, SE = 0.019, p = 0.004) in discrimination performance relative to a chorus of 8 birds only, pointing to a detrimental effect on discrimination by adding two choruses with similar texture statistics. Additionally, there is a significant difference between the paired mixture conditions (8 + 1 vs. 8 + 1*: difference = 0.079, SE = 0.017, p = 0.001; and 8 + 8 vs. 8 + 8*: difference = 0.063, SE = 0.020, p = 0.039), demonstrating that the discriminability of the exemplars is dependent on the disparities of the summary statistics of the species mixtures. Overall, performance differed across conditions, suggesting listeners’ discrimination performance was modulated by the chorus size. Additionally, the mixture conditions performance varied with the similarity of the sound statistics. The results are consistent with the notion that segregation is promoted by the disparity of the summary statistics and indicate that listeners may group bird choruses with similar statistics and segregate bird choruses with different statistics.

### Experiment 6: Do listeners stream background and foreground sounds?

Natural environments often contain multiple sound sources that extend beyond bird species mixtures to include geophysical sources, such as the sound of rain or wind, as well as biological sources distinct from birds, such as the sound of insect stridulations or frog chirps/croaks. To understand the effects of the background sounds when listening to bird vocalizations, we modified the exemplar discrimination task to include two main background sound categories: (i) geophony sounds and (ii) biophony (non-bird) sounds. The background sounds were selected to be temporally homogeneous, and likely to be perceived with time-averaged summary statistics [43]. The background sound statistics were found to be different from the bird species vocalizations (Fig 6B and 6C). The stimuli consisted of mixtures of bird vocalizations, either 1, 8 or 32 birds, without (foreground-only conditions) and with background sound sources (foreground+background conditions). The background sound level was fixed for all mixture conditions and set to the rms level of a single bird. Therefore, the foreground-to-background ratio was 0dB for 1 bird, approximately + 9dB for 8 birds, and approximately + 15dB for 32 birds. The background sound level is a simplification of the variation that is observed in nature, where the level of the signal at the receiver is dependent on several factors including source level, source distance and the propagation conditions, such as objects in the local environment [24,53,54]. Both the foreground and background sounds conform to the exemplar discrimination task, where the standard intervals use the same sound excerpt, and therefore have identical waveforms. If listeners incorporate the background sound into their judgements, we may observe a decrease in discrimination performance when comparing foreground-only sounds to foreground+background sounds.

The results for the geophony background sound conditions are shown in Fig 6E. The foreground-only condition for chorus size of 1, 8 and 32 replicated the in-lab results from Experiment 3, where performance decreased with increasing chorus size (F_2,30_ = 10.3, p < 0.001, η^2^_p_ = 0.41). When comparing the foreground-only condition to the foreground+background (geophony) condition, we did not observe a significant difference in performance for a chorus size of 1 (p = 0.894), 8 (p = 0.453), or 32 (p = 0.449). This result suggests that listeners may be segregating the background geophony sound from the bird chorus. Further, discriminability of the large chorus size of 32 with or without background was similar to the background-only condition (F_2,30_ = 0.368, p = 0.695, η^2^_p_ = 0.02), suggesting again that large bird choruses may be perceived with a summary statistics representation.

The results for the biophony (non-bird) background sound conditions are shown in Fig 6F. The birds-only condition for chorus size of 1, 8 and 32 again replicated the results from Experiment 3, where performance decreased with increasing chorus size (F_2,38_ = 31.9, p < 0.001, η^2^_p_ = 0.63). When comparing the foreground-only condition to the birds+biophony (non-bird) condition we did not observe a significant decrease in performance for a chorus size of 1 (p = 0.841), 8 (p = 0.367), or 32 (p = 0.630). This result also suggests that listeners do not incorporate the biological background sounds into the discrimination of bird species, regardless of the category of biophony constituting the background (i.e., bird vs insect or bird vs amphibian).

The results from Experiment 6 suggest human listeners may segregate the foreground sound from the background, even when the background is of a similar category (i.e., biophony). Certain mixture conditions exhibited decreased performance when comparing the foreground-only to the foreground+background condition. This decrease could be the result of listeners incorporating sound features of the background into their judgments. However, another possible explanation is that the addition of a background noise decreases the signal-to-noise ratio of the foreground sound. Although the naturalistic auditory scenes used here may be oversimplified, the results suggest foreground sounds are segregated from background sounds when perceiving auditory scenes across various geophony and biophony background conditions.

### Auditory Model revisited: Auditory grouping or segregation?

Auditory scene analysis could be implicated when listening to species mixtures. For example, in Experiment 5, the results could be explained by listeners grouping or segregating species mixtures of bird vocalizations depending on the disparity of the sound statistics. In addition, the results of Experiment 6, where bird choruses were mixed with other biophony or geophony, could be explained by listeners estimating the properties of the foreground sound separately from the background. If auditory streaming is occurring for species mixtures, we might expect a model estimating global properties of the entire auditory scene to fail.

The auditory texture observer model was used to evaluate the human performance of Experiment 5, operating on the experiment stimuli (Fig 7A). For the single species conditions, the model performance decreased with increasing chorus size, which qualitatively replicated human performance. An ablation analysis of the auditory texture model revealed the Envelope Correlation statistics class largely contributed to model exemplar discrimination performance (see S4 Fig). However, the model failed to replicate the performance of humans for the mixture condition. The model showed little difference in the 8 + 1 and 8 + 1* mixture conditions as well as between the 8 + 8 and 8 + 8* mixture conditions. The failure of the auditory texture observer model that operates on the entire auditory scene is consistent with the idea that listeners perform stream segregation under certain species mixture conditions.

**Fig 7:**
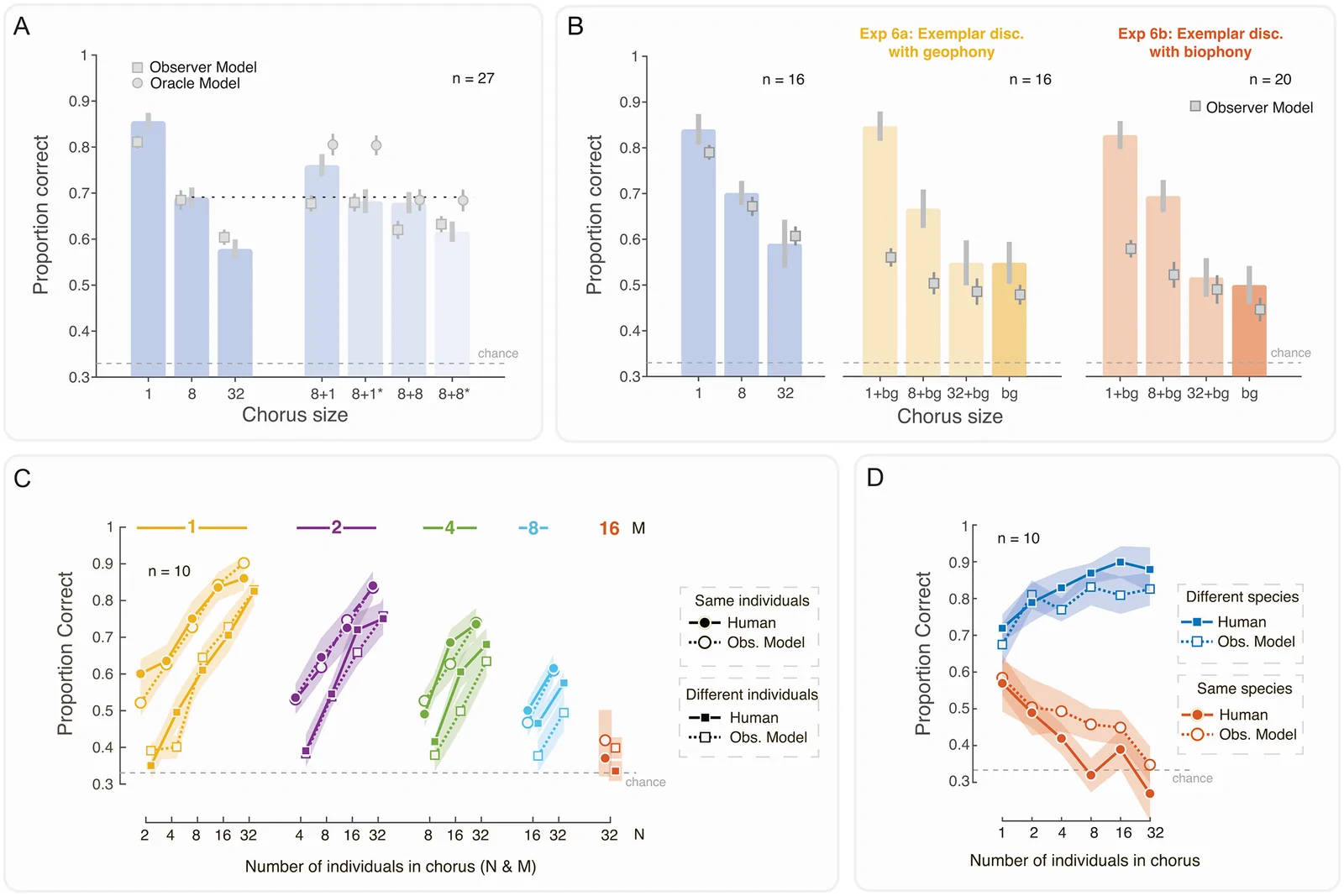
Auditory texture observer model qualitatively replicates human performance for some, but not all, species abundance and richness (mixture) experiment results. **(A)** Experiment 5 human and observer model results for species exemplar task. Two models are presented. The Observer model operates on the stimulus mixture. The Oracle model operates on the individual sources of the mixtures. Please see “Methods and Models: observer models” for more information. The Observer model qualitatively replicated the decrease in performance for single species conditions (1, 8 and 32). However, the Observer model performance deviated from human performance for species mixture conditions, with similar performance for 8 + 1 and 8 + 1* conditions and the 8 + 8 and 8 + 8* conditions. The Oracle model yielded higher performance for the mixture conditions compared to the Observer model. However, both the Observer model and Oracle model failed to capture the variation in human listeners’ performance across the mixture conditions, particularly the difference in performance between the 8 + 1 and 8 + 1* conditions and the 8 + 8 and 8 + 8* conditions. Human results (bars) show participant mean and error bars show standard error across participants. Observer model (squares) shows means and error bars show standard deviation across sound sets. **(B)** Experiment 6a and 6b human and observer model results. The observer model operates on the experiment stimuli. The Observer model qualitatively replicated human performance for single species conditions (1, 8, and 32) shown for Experiment 6a. For the Experiment 6a and 6b mixture conditions, performance deviated from humans for small chorus mixtures but was similar to humans for the large chorus mixtures and the background-only condition. **(C)** Experiment 1 human and observer model results for abundance discrimination. The Observer model performance qualitatively replicated human performance across abundance discrimination conditions, both for same individuals (circles) and different individuals (squares) conditions. Human results (solid circles and squares) show mean and shaded region shows standard error across participants. The Observer model results (open circles and squares) show mean and shaded region shows standard deviation across sound sets. **(D)** Experiment 2 human and observer model results for species discrimination. The Observer model performance qualitatively replicated human performance across abundance conditions for both the different species (blue) and same species (red) conditions. Human results (solid squares and circles) show mean and shaded region shows standard error across participants. The Observer model results (open squares and circles) show mean and shaded region shows standard deviation across sound sets.

We then implemented an “oracle” version of the auditory texture model that operated on the individual species of the mixtures. Performance was higher for the 8 + 1 and 8 + 1* conditions versus the 8 + 8 and 8 + 8* conditions. However, the oracle model results do not qualitatively replicate the change in performance between the most different and most similar conditions. Additionally, the oracle model outperformed human listeners’ performance in the 8 + 1 conditions, suggesting that humans may sub-optimally segregate the two species in the mixture. An analysis of the statistical similarity of species mixture and discrimination performance revealed a positive correlation, but this correlation was not always significant (see S5 Fig). The results are consistent with the notion that streaming may be selective and dependent on the disparities in the statistics of the bird species vocalizations.

The auditory texture model was also run on the foreground+background stimuli from Experiment 6. The human results of Experiment 6 showed listeners’ discrimination performance was not adversely affected by the presence of the geophony (Fig 6E) or biophony (Fig 6F) background sounds. The results in Fig 7B show the model qualitatively replicated human performance for the single species conditions (1, 8 and 32). However, the model deviated from human performance for the foreground+background mixture conditions when the chorus size was small (1 + bg and 8 + bg). The model performance was more comparable to that of humans when the chorus size was large or when the background was presented in isolation (32 + bg and bg). The deviation of the model from human performance in certain mixture conditions may suggest that listeners are utilizing features from the individual sound sources to perform the discrimination, rather than relying on a global representation that estimates the statistics from all of the sources in the auditory scene.

### Auditory Model revisited: Abundance discrimination as auditory texture discrimination?

Discrimination of species richness may rely, in part, on auditory stream segregation to separate the individual sources. However, discrimination of species abundance may rely on a simple estimation of the chorus size via a summary statistic. Experiments 1a and 1b showed that abundance discrimination improved with the divergence in abundance size. Could such a species abundance discrimination be facilitated by an auditory texture observer model that estimates the statistics from the entire auditory scene?

We ran the auditory texture observer model on the stimuli of Experiment 1. The observer model results qualitatively replicate human performance differences across conditions, with performance increasing with divergence of abundance number (Fig 7C). The observer model also appeared to capture the discrimination performance for the same species and different species conditions of Experiment 2 (Fig 7D). Together, the results suggest that species abundance could be represented in the auditory system with summary statistics.

## Discussion

### Summary of findings

We characterized the sensitivity of human listeners to mixtures of bird vocalizations that varied in abundance and species richness. We found that humans could discriminate bird choruses up to around 8 individuals from the same species but tended to be less sensitive to size differences between large bird choruses. This capacity is likely sufficient for listening situations in temperate forests. Discrimination performance of exemplars decreased as a function of chorus size, suggesting listeners may lose access to the temporal detail and thereafter transition to a summary statistic representation. When presented with mixtures of species-pairs, listeners’ performance was lower for mixture conditions when the sounds had similar statistics and was higher for mixture conditions when the sounds had different statistics. The results are broadly consistent with established principles of auditory scene analysis already demonstrated with artificial, music and speech stimuli [2]. The results are also compatible with recent behavioral results obtained with a subjective psychophysical paradigm (a numerosity task [10]). In addition, we implemented several computational models and found that discrimination of the mixtures could be conducted by a global estimate of the mixture properties, suggesting that auditory streaming is not necessary. In a more direct test of auditory streaming, we used an exemplar discrimination task with species mixture-pairs that were most similar and most different in terms of their texture statistics. The results showed exemplar discrimination performance was significantly higher for conditions where the mixture statistics were different, even when the total chorus size was the same. The difference in performance could be interpreted as listeners grouping mixtures of bird species when their statistics are similar and segregating mixtures of bird species when their statistics are different. Lastly, we conducted experiments with mixtures of bird vocalizations with natural biological and geophysical background sounds and found that human listeners may segregate the background sound from the foreground composed of bird vocalizations. Overall, these findings, based on objective psychophysical measurements and naturalistic stimuli, provide an initial characterization of the human capacity to perceive biodiversity through the auditory sensory modality. Moreover, the present findings suggest a potential contribution of time-averaged summary statistics, known to be implicated in texture perception. To summarize, we have attempted to link the streaming/segregation processes of auditory scene analysis to the perception of sounds in natural environments, and hypothesized the conditions under which the human auditory system may integrate acoustic details of bird vocalizations and group or segregate natural sound sources.

### Relationship to prior work

Auditory scene analysis of environmental sounds has garnered relatively little attention compared to speech and music research [55–58]. Work with environmental sounds typically used a wide variety of real-world sounds that spanned a range of environments within a single study, including sounds from domestic and urban settings to rural areas and natural parks. In our study, we focused on a single natural environment – a temperate forest – attempting to preserve the ecological balance of species and sources. Our results are consistent with previous work on natural sound perception, but by using mixtures of natural sounds we were able to expand previous findings of auditory scene analysis, specifically in the domain of sound texture perception.

The experimental stimuli used were almost entirely composed of mixtures of sound sources, challenging the listener to extract auditory features from the mixture in each discrimination task. Previous work has highlighted many auditory features that facilitate streaming, such as long-term spectral cues, pitch, temporal modulation and temporal coherence [42]. Many of these features are foundational for the perception of sound texture, which has been identified as a potential cornerstone of auditory scene analysis [45,59]. Our findings generally support the role of texture in auditory scene analysis, suggesting that background texture sounds may be segregated from foreground bird vocalizations, as was shown in Experiments 5 and 6. Furthermore, Experiment 5 introduced evidence that concurrent textures may be grouped or segregated depending on the disparities of their time-averaged statistics, suggesting listeners may be able to represent multiple concurrent texture streams.

Our findings draw parallels between texture perception and auditory scenes containing naturalistic bird vocalizations, particularly for large bird chorus mixtures. The results of Experiment 3a expand on previous findings of texture perception for multi-talker babble as well as other temporally dense sounds, like rainfall or idling traffic. Specifically, the results of Experiment 3a show that for single bird vocalizations performance was maintained across durations, suggesting listeners retain the temporal detail of the sound. However, listeners lost this ability as the number of birds in the chorus increased. The results are analogous to previous findings using temporally dense texture sounds, suggesting that regardless of the source type, these dense sound textures composed of many similar acoustic events are perceived by their time-averaged statistics.

To our knowledge, this is the first comprehensive behavioral study that attempts to estimate the auditory sensitivity of human listeners when discriminating levels of species abundance and species richness using objective, forced-choice, psychophysical measurements. This study therefore extends the pilot study conducted with human listeners by Grinfeder et al. (2025) showing that the perceived number of birds in a reconstructed forest setting was significantly higher for mixtures of bird songs from different species compared to mixtures of bird songs from the same species. Sound recordings have been used by ecoacousticians to characterize the level of biodiversity of natural environments by way of computational metrics [15,21]. The metrics attempt to assess the number of species in a sound recording by measuring global statistics across the entire sound recording. However, it has been shown that these metrics are susceptible to concurrent abiotic sound sources, such as wind or rain sounds as well as anthropophonic sounds, such as airplane noise [22,60]. Our results revealed that human listeners may segregate the sources when listening to mixtures of sounds, which suggests listeners may be able to estimate the level of biodiversity from separate auditory streams rather than from the entire sound scene. These findings further support the role of auditory scene analysis when listening to natural sounds.

The psychophysical studies conducted here are based on objective three-interval forced-choice paradigms, namely the triangular or oddity method [61–63], which allowed us to rigorously assess the sensitivity of our human observers by eliminating potential (decision-making) biases in these auditory discrimination tasks. These psychophysical experiments characterize the auditory abilities of human observers in perceiving bird biodiversity in natural settings, in contrast to a pilot study that used a subjective numerosity judgment paradigm - susceptible to response biases - based on a single observation interval and applied to bird choruses generated from data from another temperate forest [10,64]. The pilot study revealed that human observers cannot estimate more than 3 or 4 birds within a natural soundscape simulating the contribution of ambient background noise and habitat transfer function as well as the vocal behavior of birds [10]. This finding differs from the perceptual limit of 8 birds observed in the first auditory discrimination task of bird chorus size using the forced-choice triangular method, suggesting that numerosity judgments – although more ecological – are limited by higher-level factors of a cognitive nature (e.g., attentional, decision-making). Thus, the perceived richness of bird choruses in natural environments is probably greater than previously reported [10]. Still, the previous results were generally consistent with the idea that streaming mechanisms contribute to the estimation of chorus size (perceived size was greater for choruses made out of birdsongs from different species compared to choruses made out of birdsongs from the same species), in line with the outcome of the present studies.

### Limitations

Naturalistic stimuli generated from real-world recordings are often difficult to implement in perceptual experiments due to the lack of well controlled source material. The source material can be laborious to collect by an individual. However, citizen science initiatives, like the Cornell Lab of Ornithology | Macaulay Library, have amassed a large database of natural sound recordings which are accessible by researchers. Although the recording technique and environment noise were variable across samples, we were able to create a bird sound database with sufficient quality to generate the experimental stimuli and test our main hypotheses.

Species biodiversity varies from region to region. The diversity and abundance of species can vary from a few individuals in a dry desert to large groups and high levels of diversity in a tropical rainforest [65]. The sounds used in the experiments are found in temperate forests, where the level of biodiversity can be limited to several individuals from different species and small groups of individuals from the same species [48]. However, our experiments push the limit beyond what has been measured in these types of temperate forests. The creation of the chorus sizes was a compromise between ecological validity and the goal of probing the limits of auditory perception of biodiversity. Therefore, the generalizability of the results is likely relevant for small to modest chorus sizes, but more speculative for larger chorus sizes. However, a similar experimental approach could be applied using sound sources and chorus sizes for regions with higher levels of biodiversity, such as a tropical rainforest.

Another limitation of the in-lab experiments is the lack of temporal and spatial cues found in the real-world recordings. The temporal cues would relate to the temporal occurrence of the bird vocalizations, as dictated by the animal’s behavior. There is evidence to suggest that animal vocalizations are not random but are produced with temporal patterns to facilitate communication or mating calls [26–28,66]. The temporal relationship between vocalizations would inevitably affect perception. However, our experiments have laid a foundation on which alternate stimuli that include animal behavior could be incorporated. The spatial cues could be the physical location of the sound sources that would engage the sound localization and distance perception of the human auditory system. As stated throughout this paper, auditory scenes are often composed of multiple sound sources that are separated in space. The physical distance between sound sources can impact the streaming of the sources [67,68]. Our experiments did not include any spatial location or distance cues, which would have a potential effect on the streaming of different sources. Incorporating spatial location or distance cues could promote streaming and is a natural extension of many of our experiments.

It is also important to note that acoustic biodiversity can vary across moments of the day, seasons, habitats and biomes [15]. This can result in vastly different auditory scenes, which can vary from extremely sparse to highly dense sound environments. For our experimental stimuli, we sampled bird species from a single temperate forest in France. The present protocol did not consider the above-mentioned ecological factors known to affect biodiversity. For these reasons, external ecological validity of the present research that only aimed to explore the *limits* of the human auditory system is limited.

### The role of sound texture in auditory scene analysis

It has been suggested that sound texture perception is a cornerstone of auditory scene analysis, where foreground sounds are embedded in background texture [43,45,59]. However, there are only a few studies showing how texture sounds are perceived when a foreground sound is present. Evidence from behavioral studies revealed the continued monitoring of background texture statistics when interrupted by foreground noise, and that the foreground sound is not included in the estimate of the background texture statistics [69]. Additionally, studies have shown that the sensitivity to foreground outlier sounds increased with more exposure to the background texture sound [59]. This phenomenon has been shown with speech in noise studies, where speech reception thresholds decrease with more exposure to the background noise [70]. Our results provide additional evidence that listeners perceptually segregate foreground sounds from the background texture.

The role of texture in auditory scenes has previously been explored either in isolation or as part of a foreground-background texture task [43–45,47]. In the set of studies presented, we began to explore the perception of concurrent textures, and how the auditory system may be able to generate multiple concurrent statistical representations of textures. It is unclear how independent the texture statistics representations may be for concurrent textures, and whether the representation is biased when a second texture is present. Our results show that listeners could use acoustic information from concurrent textures in a discrimination task, but our experiments cannot reveal the representation of the individual texture streams.

The auditory texture model was a good candidate model that qualitatively replicated the discrimination performance of human listeners across several experiments. However, the model included several classes of statistics, and it was not clear how they contributed to the overall model performance. An ablation analysis revealed that Modulation Power and Envelope Correlation played an outsized role in the observer model performance (see S4 Fig). However, a more nuanced view of the ablation results revealed that the contribution of the statistics classes varied across experiments: performance in Experiment 4b – species mixture discrimination – appeared to depend primarily on Modulation Power, whereas performance in Experiment 5 – species mixture exemplar discrimination – appeared to depend primarily on Envelope Correlation. The ablation analysis suggests Modulation Power may drive species-level discrimination, while Envelope Correlation may drive the finer-grained similarity judgments.

### Human and machine hearing of biodiversity

The advancement of audio signal processing techniques has enabled the estimation of biodiversity from sound recordings alone [21]. Several tools have been developed that use global metrics to estimate the abundance and diversity of the species present in sound recordings. This approach has been successful as a non-invasive tool for monitoring the biodiversity of an environment. However, these global metrics are affected by geophysical sound sources, such as rain or wind sounds, as well as anthropophonic sound sources, such as airplane noise. Although our experiments do not explicitly test biodiversity estimation, the results suggest that auditory scene analysis plays an important role when monitoring sounds in natural environments. As such, any biodiversity estimation metric from a sound recording may benefit from some form of segmentation, where different sources are separated and only those sounds of interest, in this case biophony, are used in the estimate of biodiversity. The current findings may therefore inspire novel signal-processing strategies based on sound-source separation principles to assess biodiversity in a robust manner through soundscape recordings.

Biodiversity assessment using ecoacoustic indices derived from recordings of large soundscape datasets has recently been called into question for both methodological and theoretical reasons [71,72]. Nevertheless, research based on synthesis of soundscapes that manipulate the degree of avian biodiversity under fully controlled conditions, suggests that algorithms performing a preliminary decomposition of recordings into individual sound sources provide more accurate estimates of species richness than indices calculated across the entire signal [73]. Such source-separation algorithms include those based on automatic identification or regions of interest. Notably, these approaches operate on principles similar to those underlying human auditory scene analysis [74]. While the present results, which assess human auditory discrimination performance of sound mixtures, do not translate directly into estimates of the species richness of a soundscape, they nevertheless support the relevance of source separation approaches for estimating biodiversity.

### Changing biodiversity

A primary goal of ecoacoustics is to accurately monitor and detect changes in biodiversity from sound recordings [21]. Ecoacousticians monitor changes in the sound scene over a variety of timescales, from moment of the day and seasons to changes over years and decades. The human auditory system is also tuned to monitor and detect changes in the environment. Change detection in the human auditory system is typically evaluated on relatively short timescales, on the order of milliseconds to seconds as in the case of natural scenes [75], and it is unclear how sensitive listeners are to changes in the auditory environment for longer timescales, such as those monitored by ecoacousticians. There is evidence suggesting that human listeners are not sensitive to slow temporal changes in the auditory scene [76]. Our experiments indicate that listeners are sensitive to differences in the biodiversity of the auditory scene, for instance when comparing species mixtures, but it is unclear whether such a discrimination could be performed over long timescales.

### Future directions

Auditory scene analysis, or more specifically the phenomenon of auditory streaming, has a long history in behavioral experiments [2,6,39]. However, many of the behavioral results would benefit from models of computational auditory scene analysis. Models that include auditory inspired features, learned representations, and generative models are valuable tools to understand auditory processing for scenes composed of multiple concurrent sound sources [41,77,78]. The behavioral results presented in this study would benefit greatly from an auditory model of streaming. Specifically, a signal-computable model that operates on the texture statistics with added sound segregation capability would allow for the exploration of what sound texture features or statistics classes are necessary for grouping or streaming. Many of the sound texture statistics have been related to auditory streaming, and it seems plausible that an auditory texture model that can generate pairs of statistics from sound mixtures coupled with a sound texture synthesis system could extend our understanding of grouping or streaming of sound mixtures.

To understand the auditory system, behavioral experiments must be extended to use ecologically valid natural sounds for which the auditory system has developed efficient neural codes through evolution [79]. Unfortunately, natural sounds are often difficult to incorporate into psychophysical experiments due to many factors, for instance the lack of control of the sound signal as compared to artificial stimuli. In this study, we used natural sounds to create auditory scenes by superimposing waveforms from different recordings. This allowed us to test basic hypotheses about listening to natural sounds, such as bird songs, but it could be argued that the way the scenes were created, and the diversity of species and abundance, are outside of the range of ecological validity. For instance, it is unlikely for a listener to encounter 32 birds singing concurrently for the species and biome we selected. In addition, the stimuli lacked certain dimensions of natural scenes, such as source distance, position, and temporal occurrence. The source distance and position are significant cues that likely promote segregation of sound sources in complex environments. The temporal occurrence relates to the sequencing of bird vocalizations, a phenomenon that is well documented [66,80]. Any future experiments would benefit from including additional realism, or ecological validity, to know if the auditory phenomena we observed in our experiments translate to real-world listening.

Changes in biodiversity occur over a variety of timescales. However, there has been an observable decrease in acoustic biodiversity in certain biomes due to several factors, including human activity [81–84]. This change has altered the auditory scene by decreasing the species diversity and abundance of vocalizing animals, changing the human auditory experience in natural environments. An alternate anthropocentric view is that of changes in hearing capacities, such as hearing impairment [12,85,86]. Hearing impairment affects millions of individuals worldwide and the loss of detailed environmental sound perception could have negative effects on quality of life [87]. The experiments presented here could easily be extended to investigate the effects of hearing impairment on biodiversity perception and offer potential strategies to overcome the loss of environmental sound perception by way of hearing devices, such as hearing aids.

## Methods and models

### Ethics statement

Consent for the in-lab participants was obtained verbally and consent for the online participants was obtained in writing. The experiments were approved by the French National Institute of Health and Medical Research (INSERM) and by the INSERM Ethics Evaluation Committee (CEEI) (IRB00003888, IORG0003254, FWA00005831).

### Stimulus selection

#### Reference recordings.

The reference field recordings were conducted in the Parc naturel régional de la Haute Vallée de Chevreuse, France by researchers from the Muséum National d’Histoire Naturelle. The recordings were evaluated for the type and abundance of species by an expert ornithologist [48]. In total, 25 bird species were identified from the original field recordings. Accompanying the labelled field recordings were 25 high-quality reference recordings of each individual species. From the 25 identified species, 10 bird species were selected to form the basis of our bird sound database used in the experiments. The 10 bird species were selected because they were relatively common in the original field recordings in that they were often identified in the recordings by ornithologists and provided across-species diversity in their acoustic properties (Table 1). The diversity was measured as the Euclidean distance between the normalized texture statistics (Figs 1E and S2).

**Table 1:** 10 bird species used in experiments. Labels show scientific names, common names, and the number of recordings used from the Macaulay Library for each species.

Scientific Name	Common Name	Number of Recordings
Corvus corone	Carrion Crow	80
Fringilla coelebs	Common Chaffinch	132
Phylloscopus collybita	Common Chiffchaff	94
Regulus ignicapilla	Common Firecrest	80
Prunella modularis	Dunnock	124
Turdus merula	Eurasian Blackbird	113
Cyanistes caeruleus	Eurasian Blue Tit	92
Garrulus glandarius	Eurasian Jay	80
Turdus philomelos	Song Thrush	138
Phylloscopus trochilus	Willow Warbler	80

#### Bird sound database.

The experiments required the creation of a sound database for 10 bird species. The aim was to select sounds that were acoustically similar to the reference recordings for each species. Specifically, the recordings were meant to be composed of a single bird vocalization, with a stereotyped song or call, and minimal background noise. To create the database, we downloaded sound recordings from the Macaulay Library at the Cornell Lab of Ornithology. The Macaulay Library has an extensive sound database, composed of a diverse set of recordings created with varying recording setups and a wide range of geographical locations and environments. When generating our sound database, we identified 3 main issues we wanted to address: (1) recordings with incorrect species label, (2) recordings containing multiple concurrent bird vocalizations, and (3) samples with significant noise, for example background noise, sound events or recording equipment noise.

Sound recordings from the Macaulay Library database contained extensive metadata that included bird species labels and sound quality ratings. For each species, we collected 300 recordings that had the highest sound quality rating as defined on the Macaulay Library website, resulting in 3000 recordings in total.

We analyzed the recordings with the BirdNET species identification software [88]. The BirdNET software was configured to analyze segments of the recordings with a non-overlapping analysis window with a duration of 3 seconds. The BirdNET software provided a bird species label along with a species probability for each segment. The software could identify more than one species for a given segment. The 3 second recording segments were sorted based on their species probability from highest to lowest. Once the segments were identified, they were evaluated in three steps. First, the authors compared the sound segments to the reference recordings and selected those that were audibly similar. Second, segments that contained more than one label per segment were not included in the sound database. In addition, the authors listened to the sound segments and eliminated those that contained more than one bird vocalization. Third, sounds that contained significant background noise were removed from the database. For sounds with low to moderate background noise, we applied denoising to the individual sound sources using Adobe Audition noise reduction process to standardize the sounds across the database. For each sound, a noise capture was performed on a noisy segment of the recording. This noise capture was used to apply a noise reduction of between 6-12dB. Finally, the time-averaged spectrum of the sounds was normalized within-species, with the primary goal of minimizing differences in background noise and differences in the recording setup.

The procedure was used to create a sound database with at least 80 individual bird vocalizations per species and 3 segments for each individual. The final database had 1013 individual bird recordings, and with the 3 segments per individual the result was 3039 unique sounds (see Table 1 and accompanying Supporting Information S1 Table). The accompanying S1 Table file contains all sounds with Macaulay catalog ID, Species, Segment, and start/end times. Overall, the within-species sounds were relatively homogeneous in terms of their spectral and temporal acoustic properties, with example spectrograms shown in the Fig 1C as well as S1 Fig.

#### Assumptions about the database.

The real-world sounds used in our Bird Sound database were distinct recordings uploaded to the Macaulay Library. We assumed that each recording was of a unique bird. Therefore, 1 recording was equal to 1 individual bird.

#### Auditory texture model.

The auditory texture model processed an input sound waveform through linear-nonlinear-linear filter stages and measured statistics of their outputs. The filter bank stages, which were a modification of the original McDermott and Simoncelli model [43], are described in an earlier paper [45]. The first linear filter bank approximated the frequency selectivity of the cochlea. The nonlinear processing stage approximated the processing of the inner ear to the auditory nerve. The output of the nonlinear processing stage was filtered with a temporal modulation filter bank. The texture statistics measured from these representations consisted of the mean, coefficient of variation, and skewness of the cochlear envelopes, pair-wise correlations across cochlear envelopes, the power from the modulation filters, and pair-wise correlations across modulation bands.

To model the cochlear frequency analysis, sounds were filtered into subbands by convolving the input with a filterbank with different center frequencies and bandwidths. Fourth-order gammatone filters were used as they approximate the tuning properties of human auditory filters. The filterbank consisted of 34 bandpass filters with center frequencies defined by the equivalent rectangular bandwidth (ERB)_N_ scale (spanning 50Hz to 10kHz). The output of the filterbank represents the first linear processing stage from our model.

The resulting filterbank outputs were subsequently processed with a non-linear layer with a power-law compression value of 0.3, intended to model the non-linear behavior of the inner ear. The subband envelopes were then computed from the analytic signal (Hilbert transform), to approximate the transduction from the mechanical vibrations of the basilar membrane to the auditory nerve response, and subsequently downsampled to 400 Hz prior to the second linear processing stage.

The next linear processing stage filtered each cochlear subband into amplitude modulation rate subbands by convolving each envelope with a second linear filterbank. The modulation filterbank consisted of 18 half-octave spaced bandpass filters (0.5 to 200 Hz) with constant Q=2. The modulation filterbank models the selectivity of the human auditory system thought to occur in the thalamus. The modulation bands represent the output of the final processing stage of our auditory texture model.

The model input was a discrete time-domain waveform, x(t). The texture statistics were computed on the cochlear envelope subbands, $x_{k}(t)$, and the modulation subbands, $b_{k,n}(t)$, where k indexes the cochlear channel and n indexes the modulation channel. The windowing function, w(t), obeyed the constraint that $\sum_{t}w(t)=\text{1}$.

The cochlear envelope statistics include the mean, coefficient of variation, and skewness and represent the first three marginal moments. The marginal moments capture the sparsity of the time-averaged subband envelopes. The moments were (in ascending order)


$$\begin{array}{c} \begin{array}{c} \mu_{k}=\sum_{t}w(t)x_{k}(t), \end{array} \end{array}$$ (1)

$$\begin{array}{c} \begin{array}{c} \frac{\sigma_{k}^{\text{2}}}{\mu_{k}^{\text{2}}}=\frac{\sum_{t}w(t){\left(x_{k}(t)-\mu_{k}\right)}^{\text{2}}}{\mu_{k}^{\text{2}}}\;, \end{array} \end{array}$$ (2)

$$\begin{array}{c} \begin{array}{c} \eta_{k}=\frac{\sum_{t}w(t){\left(x_{k}(t)-\mu_{k}\right)}^{\text{3}}}{\sigma_{k}^{\text{3}}}, \end{array} \end{array}$$ (3)

$$\begin{array}{c} k\in [\text{1}\ldots \text{3}\text{4}]\text{ in each case}. \end{array}$$

Pair-wise correlations were computed between the eight neighboring cochlear bands. The correlation captures broadband events that would activate cochlear bands simultaneously and is written as


$$\begin{array}{c} \begin{array}{c} c_{jk}=\frac{\sum_{t}w(t)\left(x_{j}(t)-\mu_{j}\right)\left(x_{k}(t)-\mu_{k}\right)}{\sigma_{j}\sigma_{k}}\;, \end{array} \end{array}$$ (4)

$$\begin{array}{c} j,k\in [\text{1}\ldots \text{3}\text{4}]. \end{array}$$

The power at different modulation rates was measured as the modulation subband variance normalized by the corresponding total cochlear envelope variance. The modulation power measure was


$$\begin{array}{c} \begin{array}{c} \sigma_{k,n}=\frac{\sum_{t}w(t){\left(b_{k,n}(t)-\mu_{k,n}\right)}^{\text{2}}}{\sigma_{k}^{\text{2}}}, \end{array} \end{array}$$ (5)

$$\begin{array}{c} \;k\in [\text{1}\ldots \text{3}\text{4}],\;n\in [\text{1}\ldots \text{1}\text{8}]. \end{array}$$

Finally, the texture representation included correlations between modulation subbands of neighboring cochlear channels. Some sounds have correlations across many modulation subbands (e.g., rain drops), whereas others have correlations only between a subset of modulation subbands (bird vocalizations or insect chirps). These correlations are given as


$$\begin{array}{c} \begin{array}{c} c_{jk,n}=\frac{\sum_{t}w(t)\left(b_{j,n}(t)-\mu_{j,n}\right)\left(b_{k,n}(t)-\mu_{k,n}\right)}{\sigma_{j,n}\sigma_{k,n}}, \end{array} \end{array}$$ (6)

$$\begin{array}{c} j\in [\text{1}\ldots \text{3}\text{4}],\;(k-j)=[\text{1},\;\text{2}],n\in [\text{3}\;\text{5}\;\text{7}\;\text{9}\;\text{1}\text{1}\;\text{1}\text{3}]. \end{array}$$

The texture statistics identified here resulted in a parameter vector ζ.

### Species distance metric (Normalized Euclidean distance of sound statistics)

To characterize the differences in our bird database, we measured the normalized Euclidean distance between sound pairs. We began by measuring the summary statistics of all of the sounds with the auditory texture model. The measured statistics were z-scored across the sounds for each of the 6 statistics classes. The distance was computed for each statistics class and then averaged across the 6 classes, which gave a single number as the distance between two sounds in our database. Finally, the sounds were normalized by the largest distance between two sounds, such that the smallest distance was 0 and the largest distance was 1. The results can be found in Figs 1E and S2. To assess species separability, the texture statistics were classified using an error-correcting output codes (ECOC) classifier (one-versus-all coding), in which each binary learner was a GentleBoost ensemble of 100 decision trees. Accuracy was assessed with 10-fold cross-validation (S3 Fig).

### Auditory grouping features

We characterized the sound mixtures used in Experiment 4b with six acoustic cues commonly associated with sound source grouping or segregation (Fig 4E). The six cues were: (1) frequency spectrum (envelope mean), (2) envelope coefficient of variation (broadband amplitude modulation), (3) envelope correlation (envelope co-modulation), (4) amplitude modulation power, (5) loudness, and (6) pitch. For each cue, we computed the Euclidean distance between the two species in the sound mixture.

The cues 1–4 were measured from the corresponding statistics from the auditory texture model. The time-averaged statistics resulted in a vector for the two sounds in the mixture. The vector varied in length depending on the statistics class.

The loudness cue, 5, was based on a dynamic loudness model with a default parameter configuration [89]. The loudness between the two sounds was taken as the time-averaged loudness for the 147 audio frequency bands between 1kHz and 15kHz. The Euclidean distance was computed between the 147 element vectors of the two mixture sound species.

The pitch cue, 6, was computed with the *YIN* software package that measures the fundamental frequency, also known as f-zero, of an input sound [90]. The YIN software was configured with a base fundamental frequency of 12Hz and a hop size of 512 samples, a low pass filter of 12kHz, a buffer size of 40,000 samples, and a threshold of 0.5, otherwise all the parameters were configured as default. The output of the YIN processing was a time-domain f-zero. For each sound, the mean and variance of the time-domain f-zero were computed, resulting in two parameters per sound. The mean and variance were z-scored across sounds so that the two parameters were on the same scale. Lastly, the Euclidean distance was computed between two parameters of the sound species mixtures.

### Observer models

Seven observer models with different measured parameters were implemented, as shown in Fig 5A and 5D. The models are (1) auditory texture, (2) frequency spectrum, (3) envelope coefficient of variation (4) envelope correlation, (5) amplitude modulation power, (6) loudness, and (7) pitch. The observer model had a similar structure for all the different model types (see Fig 5A). The model measured the parameters from the three intervals on each trial of the experiment over the entire extent of the interval. The Euclidean distance between the three intervals was computed and the interval that was most different from the other two was selected.

The auditory texture model and YIN pitch model were unique in that they consisted of multiple classes of statistics. Because the different classes of statistics have different units, each statistics class was z-scored across all the experiment sounds.

The observer models where performance was greater than human performance contained additive internal noise applied to the measured parameter. For Experiment 4, the noise was tuned to the mean performance across experiment conditions. For Experiment 5, where only the auditory texture model was evaluated, the model noise was tuned to best match performance across the bird-only conditions, as described in the next paragraph.

The auditory texture model was unique in that it had different noise weightings for the different statistics classes. To achieve performance comparable to humans, a statistics class dependent noise weighting was used. The weighting was determined by fitting the model to the single species conditions of 1, 8 and 32 birds of Experiment 5. The model noise weighting for the six classes of statistics was (1) envelope mean = 0.06, (2) envelope coefficient of variation = 0.06, (3) envelope skewness = 0.11, (4) envelope correlation = 0.06, (5) modulation power = 0.44, (6) modulation correlation = 0.27. The weighting was fixed for all auditory texture model results in Experiment 5 and 6 as well as the abundance discrimination model results of Experiment 1 and 2, all of which are shown in Fig 7.

The oracle observer model included in Fig 7A operated on sounds of the individual species of the mixture. The oracle model was a variant of the auditory texture model that took as input the individual species sound sources. Simply put, the oracle model can be thought of as an optimal stream segregation model, that measures the statistics from the individual sources of the mixture. For instance, the oracle model input for the 8 + 1 condition would be the 8 individuals from a species in isolation, as well as the 1 individual from a different species in isolation, resulting in two streams and independent sets of statistics. The oracle model decision was based on the species with the largest distance between the target and standard.

The model error bars on Fig 5B were obtained by fitting the model to bootstrapped human data. First the mean of the bootstrapped human data was computed. Next, the noise parameter was tuned to the bootstrapped human data. This process was repeated 1,000 times. The resulting error bars show the 95% confidence intervals (2.5th-97.5th percentiles) across bootstrapped model fits. The error bars shown in Fig 5C were obtained by fitting the model to half of the human data and then measuring the error on the left-out data. This process was repeated 1,000 times. The error bars show ±1 SD (16th and 84th percentiles) across 1,000 random splits. The error bars for Fig 7 show standard deviation across sound sets. For Fig 7A and 7B, there were 50 sound sets, where one sound set was equivalent to that heard by one participant. For Fig 7C and 7D, there were 10 sound sets, which correspond to each of the 10 participants in Experiment 1a and 1b. In that way, the model operated on the same stimuli as the human listeners.

### Experiments

Experiments were run both in-lab and online. The in-lab experiments were conducted at the testing facilities in the Département d’Études Cognitives (DEC) at the École Normale Supérieure in Paris, France. The stimuli were presented to listeners in a soundproof booth over headphones. The sound playback equipment included an Apple MacMini computer running MATLAB (Mathworks) and Psychtoolbox, an RME Fireface soundcard, and Sennheiser HD650 headphones. The online experiments were hosted on local servers at École Normale Supérieure.

In-lab subject recruitment was conducted via the platform RISC (*Relais d’Information sur les Sciences de la Cognition, UMS CNRS 332*) at École Normale Supérieure. In-lab participants were self-reported normal hearing listeners. No inclusion/exclusion criteria were applied for the in-lab participants. Online subject recruitment and payments were conducted using the Prolific online experiment platform. The online participants were required to pass a headphone screening task in order to participate in the main experiment.

The presentation level was different between the in-lab and online experiment. The in-lab experiments were calibrated to have a presentation level of 60dB SPL. The online experiment relied on users listening to a calibration noise signal and setting the volume on their playback system to a comfortable level. The rms level of the calibration noise signal was the same as that of the experiment stimuli.

Individual participant experiment data can be found in S2 Table.

#### Experiments 1 and 2: Abundance discrimination and species discrimination (in-lab).

The stimuli for Experiment 1 and 2 were created for a 3-interval oddity task, where two intervals were similar, referred to as the standard, and one interval was different, referred to as the target.

Stimuli. For each trial, we constructed stimuli composed of N or M number of superimposed bird vocalization audio samples. Each sample corresponded to a single bird vocalization. The values of N and M were 1, 2, 4, 8, 16, or 32, corresponding to a chorus size range of 1 bird singing to 32 birds singing. The original audio samples were 3 seconds in duration. The audio segment contributing to the trial interval was 2 seconds in duration and was randomly sampled from the original audio sample with an onset time between 0 and 1 seconds.

Two versions of the stimuli were created: (Experiment 1a) where the standard was created from audio samples from the same recording(s), and thus the same individuals, and (Experiment 1b) where the standard was created from audio samples from different recordings, and thus different individuals. For Experiment 1, all permutations of the chorus size of N and M were created. In this experiment the standard and target intervals were composed of vocalizations from the same species. In the main experiment conditions, N never equaled M. This resulted in 30 chorus size comparisons for each of the 10 species, resulting in 300 trials in total.

The stimuli for Experiment 2 included two conditions. The first condition, N equaled M, resulted in two choruses of the same size but composed of different individuals, referred to as “same species”. The trials for the first condition were intermixed with Experiment 1a. The second condition, N equaled M, but the standard and the target were composed of samples from different species, referred to as “different species”. The second experiment trials were intermixed with both Experiment 1a and 1b.

A unique set of stimuli were generated for each participant, varying both in the sound samples used to create the bird choruses and the sample onset time. For Experiment 2, the stimuli for different species condition were randomly sampled and unique for each participant.

Each trial was composed of three, 2 second intervals of sound. A raised cosine window with 20ms onset/offset ramps was applied to 2 second intervals of sound. The stimuli were presented at 60dB SPL. Background pink noise, high-pass filtered at 50Hz with a 5^th^ order Butterworth filter applied with a zero-phase forward-reverse filter, was added to the stimuli at 40dB SPL to mask any extraneous low-level sounds that might be present in the original samples. Intervals were separated by a 500ms segment of silence. The sample rate was 20kHz.

Procedure. Experiment 1a consisted of 300 trials intermixed with 120 trials for Experiment 2 (60 trials for the within-species condition and 60 trials for the across-species condition). Experiment 1b consisted of 300 main experiment trials intermixed with 60 trials for Experiment 2 (second condition). On each trial, participants judged which interval was different from the other two.

Participants. A total of 10 participants were recruited for the experiment. Of these participants, 7 identified as female and 3 as male. The average age of participants was 24.3 (s.d. = 3.5).

Sample size. In order to determine the sample size required for Experiment 1 and 2, we ran a version of the same species discrimination task online. The experiment was similar to Experiment 2 for the same species condition, with the chorus size varying from 2 to 32. We collected data from 22 participants. We found that detecting a change in performance across conditions using a repeated measures analysis of variance (ANOVA) statistical method varied from 7% for 2 participants to 100% for 22 participants. We decided to set a detectability level of 80%, which could be achieved with 10 participants for experiments 1 and 2.

Statistics and data analysis. We performed a repeated measures ANOVA to analyze the effect of chorus size on discrimination performance. We assumed the data was normally distributed which we verified with an informal analysis. Mauchly’s test indicated that the assumption of sphericity had not been violated. For each main effect and interaction of interest, we reported F-statistics and p-values.

#### Experiment 3: Exemplar and species discrimination (in-lab).

The stimuli for Experiment 3 were created for a 3-interval oddity paradigm of an exemplar discrimination task. In the task, two intervals were the same, referred to as the standard, and one interval was different, referred to as the target.

Stimuli. For the Experiment 3a exemplar discrimination task, all three intervals of sound were the same chorus size. For each trial, we created stimuli consisting of a chorus size of 1, 8 or 32 birds. The original audio samples were 3 seconds in duration. The audio segment contributing to the trial interval was 2 seconds in duration and was randomly sampled from the original audio segment with an onset time between 0 and 1 seconds. The sound consisted of two standard intervals that were identical waveforms and a target that was a different waveform from the same set of audio samples. The duration of the intervals was either 50ms or 2 seconds.

The short 50ms segments and the longer 2s segments were selected to have the same time-averaged spectrum. We did this to ensure there was sufficient sound energy for the short 50ms segments. We began by measuring the time-averaged spectrum of the 2s segment via the Auditory Texture Model. We then randomly sampled 50ms segments from the original sound and found the segment where the time-averaged spectrum was most similar (smallest distance) to the 2s segment. This was selected as the 50ms segment.

A 10ms raised cosine window was applied to the onset/offset of the sound intervals. Intervals were separated by a 500ms segment of silence. The sample rate was 20kHz.

Experiment 3b consisted of both an exemplar discrimination task and a species discrimination task. The exemplar discrimination task stimuli were constructed using the same method as Experiment 3a for the chorus size of 32. The species discrimination task consisted of two standard intervals that were generated from sound samples of one species and a target interval that was created from sound samples of a different species. The species pairs were randomly selected for each trial. A unique set of stimuli were generated for each participant, varying both the sound samples used to create the bird choruses as well as the sample onset times.

Each trial consisted of three intervals of sound. A raised cosine window with 10ms onset/offset ramps was applied to intervals of sound. The stimuli were presented at 60dB SPL. Background pink noise, high-pass filtered at 50Hz with a 5^th^ order Butterworth filter applied with a zero-phase forward-reverse filter, was added to the stimuli at 40dB SPL to mask any extraneous low-level sounds that might be present in the original recordings. Intervals were separated by a 500ms segment of silence.

Procedure. Experiment 3a consisted of 100 trials for each of the two durations and three chorus size conditions, resulting in 600 trials in total. Experiment 3b consisted of 100 trials for the two durations and two main experiment conditions, exemplar and species discrimination, resulting in 400 trials. On each trial, participants judged which interval was different from the other two.

Participants. For experiment 3a, a total of 9 participants were recruited for the experiment. Of these participants, 5 identified as female and 4 as male. The average age of participants was 24.5 (s.d. = 3.3). For experiment 3b, 8 participants were recruited for the experiment. Of these participants, 4 identified as female and 4 as male. The average age of participants was 27.4 (s.d. = 4.2).

Sample size. To determine the sample size required to obtain stable results, we ran an impoverished version of the experiment for the exemplar discrimination task. The stimuli and task were similar to Experiment 3a, with a chorus size of 1, 8, and 32 and interval duration of 2s. Split-half reliability was computed by dividing the sample in half and measuring the Pearson correlation coefficient. We repeated the procedure for 10,000 random splits. The split half reliability increased from 0.64 to 0.89 with splits from 2 to 6. We therefore targeted 8 participants in order to reach a split-half reliability greater than 0.9. We assumed the target number of participants would also yield stable results for Experiment 3a short duration conditions and the species discrimination Experiment 3b.

Statistics and data analysis. We performed a repeated measures analysis of variance (ANOVA) to analyze the effect of chorus size on discrimination performance. We assumed the data was normally distributed which we verified with an informal analysis. In addition, we used a paired, two-tail t-test to check for a significant change in performance as a function of duration for each chorus size.

#### Experiment 4: Species mixture discrimination (online).

The stimuli for Experiment 4 were created for a three-interval oddity task, where two standard intervals were similar, and the target interval was different.

Stimuli. In the Experiment 4a species mixture discrimination task, all three intervals of sound had the same number of birds. For each trial, we created mixture stimuli consisting of 2, 4, 8, 16 or 32 birds. There were three conditions: (1) different species discrimination, (2) mixture of species discrimination, and (3) same species discrimination. The different species discrimination trials consisted of two standard intervals created from one species and the target interval created from a different species. The mixture discrimination task consisted of trials where either the standard or the target was created from a mixture of two species. The mixture interval(s) always had an equal number of individuals from each species. For instance, the mixture of size 4 consisted of 2 birds from one species and 2 birds from a different species. The species mixtures were randomly selected. The same species discrimination task consisted of trials where the standard and target intervals were generated from samples of the same species but different recordings, and thus different individuals.

The stimuli for Experiment 4b included the same three conditions as Experiment 4a, but for a chorus size of 16 birds. The size 16 was selected because the results of Experiment 4a yielded the largest difference between the three conditions. The stimuli for Experiment 4b were created using the same procedure as Experiment 4a. However, in Experiment 4b the mixtures were not randomly selected but covered all possible combinations across the 10 reference species. This resulted in 45 species pairs in total.

For both experiments, the original audio samples were 3 seconds in duration. The audio segment contributing to the trial interval was 2 seconds in duration and was randomly sampled from the original audio segment with an onset time between 0 and 1 seconds. The duration of the intervals was 2 seconds. A 20ms raised cosine window was applied to the onset/offset of the sound intervals. Intervals were separated by a 400ms segment of silence. The stimuli were low-pass filtered at 10kHz with a sample rate of 48kHz.

Procedure. Experiment 4a consisted of 3 sections: (1) headphone check, (2) training, and (3) main experiment. First, participants performed a 6-trial binaural level discrimination task to ensure the listeners were wearing headphones [91]. Participants had to exceed 65% correct on the headphone check in order to proceed to the training and main task.

Next, the participants performed a 10-trial training task with a random sample of the three discrimination conditions. Participants received feedback in the training task. Participants had to exceed 50% correct to continue to the main section. The main section of Experiment 4a consisted of 50 trials for each of the three experiment conditions resulting in 150 trials in total. The main section of Experiment 4b consisted of 90 species discrimination trials, 180 mixture discrimination trials and 30 individuals discrimination trials, resulting in 300 trials in total. On each trial, participants judged which interval was different from the other two.

Participants. For Experiment 4a, a total of 24 participants were recruited. Of these participants, 8 identified as female and 16 as male. The average age of the participants was 34.3 (s.d. = 14.1). For Experiment 4b, 23 participants were recruited for the experiment. Of these participants, 11 identified as female and 12 as male. The average age of the participants was 40.1 (s.d. = 11.9).

Sample size. To determine the sample size required we used pilot data from a similar oddity discrimination task for the same species condition. This data was the same pilot data used to justify the sample size for Experiments 1 and 2. We aimed for a sample size of 20, which would give a 100% chance of detecting a difference across the within-species conditions using a repeated measures ANOVA analysis. We aimed for higher sample size for Experiment 4 because it was assumed the variability in judgements for the mixture condition could be higher compared to the within-species and across-species conditions.

Statistics and data analysis. For Experiment 4a, we performed a repeated measures analysis of variance (ANOVA) to analyze the effect of chorus size on discrimination performance for the species, mixture and individuals conditions. We assumed the data was normally distributed which we verified with an informal analysis.

For Experiment 4b, we compared the discrimination performance for each of the 45 sound pairs to 6 acoustic features commonly associated with auditory grouping. To obtain the distance measure for each sound pair, we generated 100 exemplars of a chorus size of 8 for each of the 10 species. The chorus size of 8 was selected as it was the same size as the mixture interval in Experiment 4b (i.e., 8 birds from species A + 8 birds from species B). Then, for each acoustic feature, we measured the parameters as described in the Auditory Grouping features section. Next, we computed the distance between each pair of species, resulting in 45 distance values. Lastly, the R-squared correlation was computed (via Pearson's R) between the acoustic feature distance and the human discrimination performance on the mixture conditions. The significance was reported as a p-value.

#### Experiment 5: Species mixture exemplar discrimination (in-lab and online).

The stimuli for Experiment 5 were created for a 3-interval oddity task. The task was an exemplar discrimination, where the two standard intervals were the same waveform and the target interval was a different waveform.

Stimuli. The stimuli were created for two sets of conditions. The first set of conditions had a chorus size of 1, 8 or 32 and were generated from a single species. The stimuli were created using the same method as Experiment 3a.

The second set of conditions were created from mixtures of two species. We began by measuring the statistics for each of the 10 bird species for all the 3 second samples. Then, we computed the Euclidean distance of the statistics between pairs of samples. Next, we identified the pairs of species that had the largest distance (most different species) and those with the smallest distance (most similar species). The list of species mixtures for the most different and most similar conditions are shown in Table 2.

**Table 2:** Bird species mixtures used in Experiment 5. The reference species is mixed with either the most different species for the “8+1” and “8+8” conditions and the most similar species for the “8+1*” and the “8+8*” conditions. Pairs were assigned with the constraint that each species appeared only once per condition.

Reference Species	Most Different Species	Most Similar Species
Carrion Crow	Willow Warbler	Eurasian Jay
Common Chaffinch	Common Firecrest	Willow Warbler
Common Chiffchaff	Eurasian Blackbird	Eurasian Blue Tit
Common Firecrest	Song Thrush	Dunnock
Dunnock	Eurasian Jay	Common Firecrest
Eurasian Blackbird	Eurasian Blue Tit	Song Thrush
Eurasian Blue Tit	Common Chaffinch	Common Chiffchaff
Eurasian Jay	Dunnock	Carrion Crow
Song Thrush	Common Chiffchaff	Eurasian Blackbird
Willow Warbler	Carrion Crow	Common Chaffinch

From the species distance analysis, we created four conditions with mixtures of different size choruses in the following configurations: (1) pair of species that were most different for a chorus size of 8 and chorus size of 1, or the “8+1” condition, (2) pair of species that were most similar for a chorus size of 8 and chorus size of 1, or the “8+1*” condition, (3) pair of species that were most different for a chorus size of 8 and a chorus size of 8, or the “8+8” condition, (4) pair of species that were most similar for a chorus size of 8 and a chorus size of 8, or the “8+8*” condition. For each of the mixture conditions, the rms level was normalized for each individual bird recording contributing to the chorus size. Therefore, the 8 + 1 and 8 + 1* conditions, the chorus size of 1 was -9dB relative to the chorus of 8, whereas the 8 + 8 and 8 + 8* conditions had roughly equivalent levels (i.e., relative difference between species choruses in the mixture was approximately 0dB).

There were 7 conditions in total, with 3 single species conditions (“1”, “8”, and “32”) generated from a single species and 4 conditions (“8+1”, “8+1*”, “8+8”, and “8+8*”) generated from mixtures of pairs of species. For each condition the interval duration was 2s, with a 20ms raised cosine for the onset/offset and an inter-stimulus-interval of 500ms. The stimulus presentation level for the in-lab participants was 60dB SPL and the sample rate was 20kHz. For the online experiments the presentational level was set using a calibration noise as described in the Experiments methods section.

Procedure. The main experiment for the in-lab and online experiments consisted of 5 trials for each of the 10 species and each of the 7 conditions, resulting in 350 trials in total. On each trial, participants judged which interval was different from the other two.

The in-lab experiment consisted of the 350 main experiment trials. The online experiment consisted of 3 sections: (1) headphone check, (2) training, and (3) main experiment. First, participants would perform a 6-trial level discrimination task to ensure the listeners were wearing headphones [91]. Participants had to exceed 65% correct on the headphone check in order to proceed to the training and main task. Next, the participants performed an 18-trial training task with a random sample of single species conditions for chorus sizes of 1, 8, and 32. Participants received feedback in the training task. If performance was greater than 50%, participants continued to the main experiment.

Participants. A total of 27 participants were recruited for the experiment. 10 participants completed the experiment in-lab and 17 participants completed the experiment online. Of these participants, 11 identified as female and 16 as male. The average age of participants was 34.7 (s.d. = 11.5).

Sample size. To determine the sample size required to obtain stable results, we ran a version of the exemplar discrimination task. The stimuli and task were similar to the Experiment 5, except the rms level for the individual species was equivalent for the 8 + 1 and 8 + 1* conditions. We ran the analysis on the conditions of 8 + 1, 8 + 8 and 8 + 8*. The Split-half reliability was computed by dividing the sample in half and measuring the Pearson correlation coefficient. We repeated the procedure for 10,000 random splits. The split half reliability increased from 0.25 to 0.65 with splits from 2 to 6. Extrapolating beyond splits from 2 to 10, we initially targeted 10 participants with the aim of reaching a split-half reliability greater than 0.9. However, we found that more participants were required to detect a statistical difference between the mixture conditions.

Statistics and data analysis. We performed a repeated measures analysis of variance (ANOVA) to analyze the effect of chorus size on discrimination performance. We assumed the data was normally distributed which we verified with an informal analysis. The repeated measures ANOVA found a significant difference across all 7 conditions (1, 8, 32, 8 + 1, 8 + 1*, 8 + 8, and 8 + 8*), F_6,156_ = 47.69, p <  0.001, η²_p_ = 0.65. The Greenhouse-Geisser correction was applied to account for potential violations of sphericity, and the effect remained significant following correction (p < 0.001). Next, we performed repeated measures ANOVA, corrected for multiple comparisons (Bonferroni) between the chorus of 8 and the 4 mixture conditions (8 + 1, 8 + 1*, 8 + 8, and 8 + 8*), resulting in pairwise comparisons among five conditions. See Table 3 for a list of the p-values for each comparison.

**Table 3:** Experiment 5, chorus of 8 and mixture condition (8 + 1, 8 + 1*, 8 + 8, and 8 + 8*) difference, standard error, and significance values (p-values) for repeated measures ANOVA corrected (Bonferroni) for multiple comparisons. The highlighted results show the main comparisons presented in Fig 6 (Experiment 5), with significant differences between the 8 + 1 and 8 + 1* conditions and the 8 + 8 and 8 + 8* conditions, but no significant difference between the 8 + 1* and 8 + 8 conditions.

Comparison	Difference (proportion correct)	Standard Error	p
8 vs. 8 + 1	0.070	0.017	.004
8 vs. 8 + 1*	0.009	0.017	1.000
8 vs. 8 + 8	0.012	0.018	1.000
8 vs. 8 + 8*	0.075	0.019	.004
8 + 1 vs. 8 + 1*	0.079	0.017	.001
8 + 1 vs. 8 + 8	0.081	0.020	.003
8 + 1 vs. 8 + 8*	0.144	0.017	<.001
8 + 1* vs. 8 + 8	0.003	0.022	1.000
8 + 1* vs. 8 + 8*	0.066	0.017	.008
8 + 8 vs. 8 + 8*	0.063	0.020	.039

#### Experiment 6: Source mixture exemplar discrimination (online).

The stimuli for Experiment 6 were created for a 3-interval oddity task. The experiment was an exemplar discrimination task, where the two standard intervals were the same waveform and the target interval was a different sample from the same set of sources, and thus had a different waveform.

Stimuli. The stimuli were created for two main condition types. The first set of conditions had a chorus size of 1, 8 or 32 and were generated from a single species. The stimuli were created using the same method as Experiment 3a. These represent the foreground-only conditions.

The second set of conditions consisted of a chorus size of 1, 8 or 32 generated from a single species mixed with a background sound, referred to as the foreground+background conditions. The two categories of background sounds were geophony (environmental sounds) type (Experiment 6a) or a biophony (biological, non-bird sounds) type (Experiment 6b). There were 9 geophysical and 9 biological background sounds used in the experiment (see Table 4). The 9 background sounds were randomly paired with a bird species of a given chorus size. The background sound was set to the same rms level as an individual bird recording. This resulted in an SNR of 0dB for the 1-bird mixture condition, 9dB for the 8-bird mixture condition, and 15dB for the 32-bird mixture conditions.

**Table 4:** Background sounds used in Experiment 6. There were 9 geophysical background sounds (geophony) for Experiment 6a and 9 biotic background sounds (biophony) for Experiment 6b.

Geophony	Biophony
Heavy rain falling and dripping	Many bees flying around
Heavy rain on a hard surface	Cicada close
Jungle rain	Insect cricket
Fast running river	Lonely cricket
River running over shallows	Crickets at night
Small river	Crickets
Wind whistling v1	Florida frogs gathering
Wind whistling v2	Frogs
Wind moaning	Treefrog symphony with crickets

In addition to the foreground-only and foreground+background conditions, there was a background-only condition and a control condition. The background-only condition did not include any foreground bird sounds. The control condition was a different species discrimination, similar to Experiment 2, where two standard intervals were sampled from one species and a target interval that was sampled from a different species.

There were 8 conditions in total, with three foreground-only conditions (“1”, “8”, and “32”), three foreground-background conditions (“1+bg”, “8+bg”, “32+bg”), one background-only condition (“bg”), and a control condition (present in experiment data file S2 Table). For each condition the interval duration was 2s, with a 20ms raised cosine for the onset/offset and an inter-stimulus-interval of 400ms. The stimuli were low-pass filtered at 10kHz with a sample rate of 48kHz.

Procedure. Experiment 6 consisted of 3 sections: (1) headphone check, (2) training task, and (3) main task. First, participants would perform a 6-trial level discrimination task to ensure the listeners were wearing headphones [91]. Participants had to exceed 65% correct on the headphone check in order to proceed to the training and main task. Next, the participants also performed a 12-trial training task with a combination of foreground+background discrimination task for chorus sizes of 1 + bg, 8 + bg, and 32 + bg. Participants received feedback in the training task. The main experiment consisted of 9 trials for each condition, resulting in 72 trials in total. For the mixture conditions, each background sound was presented once. On each trial, participants judged which interval was different from the other two.

Participants. For Experiment 6a, a total of 28 participants were recruited for the experiment. 16 participants continued to the training and main experiment. Of these participants, 7 identified as female and 9 as male. The average age of the participants was 39.3 (s.d. = 7.8). For Experiment 6b, a total of 24 participants were recruited for the experiment. 20 participants continued to the training and main experiment. Of these participants, 11 identified as female, 8 as male, and 1 did not report/other. The average age of the participants was 41.4 (s.d. = 11.1).

Sample size. To determine the sample size required to obtain stable results, we ran a version of the Experiment 6a for the exemplar discrimination task with 25 participants. The stimuli and task were similar to the current experiment, with a slightly different version of the stimuli. The split-half reliability was computed by dividing the sample in half and measuring the Pearson correlation coefficient for the 3 chorus sizes of 1, 8 and 32 for both the foreground-only conditions and the foreground+background conditions. We repeated the procedure for 10,000 random splits. The split half reliability increased from 0.53 to 0.96 for the birds only condition and 0.28 to 0.92 for the mixture condition with splits from 2 to 24. We targeted 16 participants in order to reach a split-half reliability greater than 0.8. We assumed the target number of participants would also yield stable results for Experiment 6b.

Statistics and data analysis. We performed a repeated measures analysis of variance (ANOVA) to analyze the effect of chorus size on discrimination performance for both the foreground-only and foreground+background conditions. We assumed the data was normally distributed which we verified with an informal analysis. In addition, we used a paired, two-tail t-test to check for a significant change in performance between the foreground-only and foreground+background conditions.

## Supporting information

S1 Fig Bird species song cochleagrams. (PDF)

S2 Fig Normalized Euclidean distance of summary statistics for individual sound pairs of Bird Sound database. (PDF)

S3 Fig Multi-class classification of the 10 bird species using an ECOC classifier with boosted decision-tree learners. (PDF)

S4 Fig Ablation of Auditory Texture Model for Species Mixture Discrimination Task (Experiment 4) and Species Mixture Exemplar Discrimination Task (Experiment 5). (PDF)

S5 Fig Scatter plots of the normalized Euclidean distance and human proportion correct (Experiment 5). (PDF)

S1 Table Bird Database (Cornell Lab of Ornithology | Macaulay Library) reference IDs. (XLSX)

S2 Table Human subject experiment data. (XLSX)

S1 Text Photograph Citations (Fig. 1C). (PDF)
