# Supplementary material for: Human perception of avian biodiversity in naturalistic auditory scenes
Source: PLoS Comput Biol. 2026 Sep 21;22(9):e1014775. doi: 10.1371/journal.pcbi.1014775 (PMC13645227; doi:10.1371/journal.pcbi.1014775)
Supplement: Supplementary file 1 [file pcbi.1014775.s001.pdf]

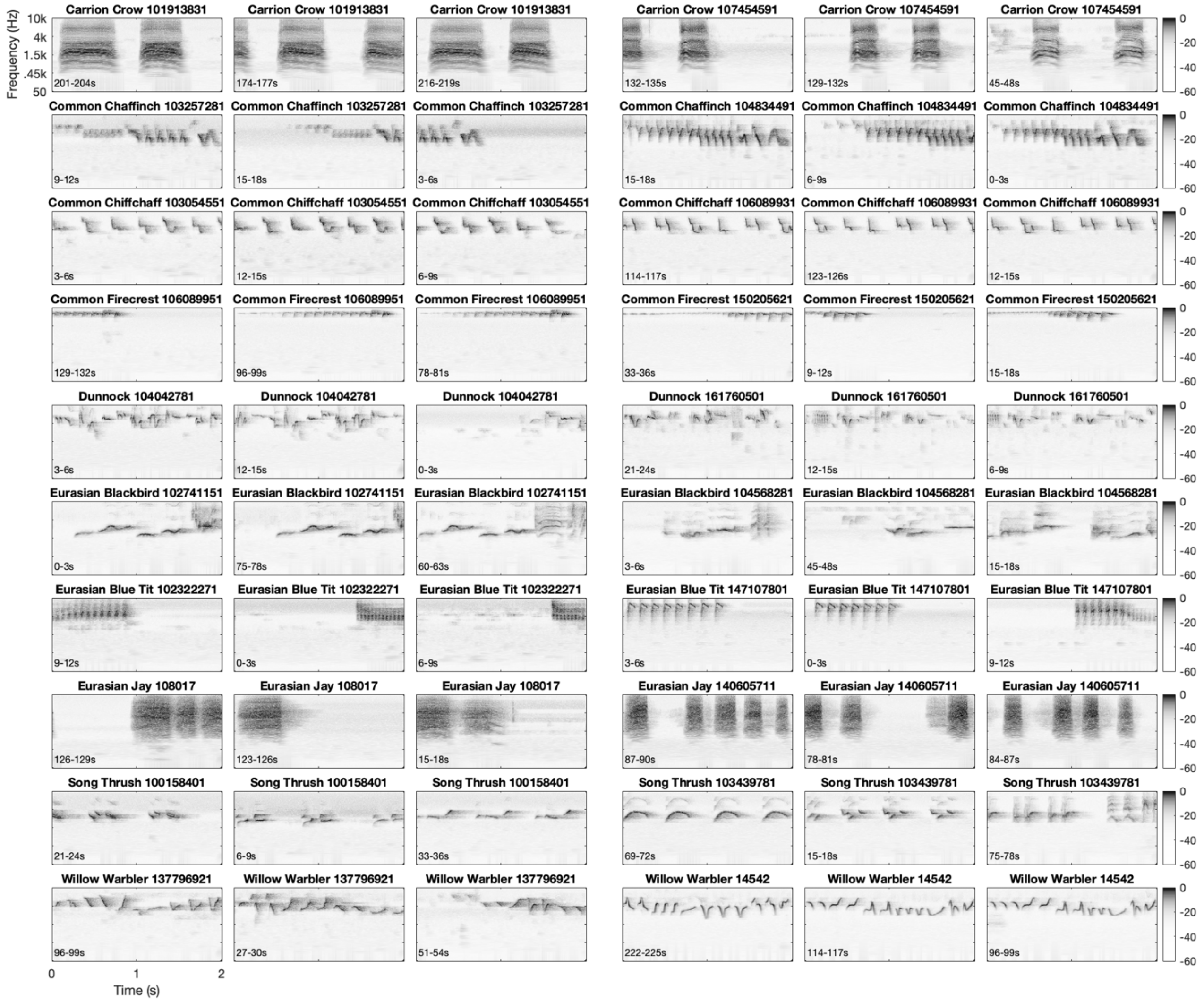

Figure S1 – Bird species song cochleagrams. Each row shows a one of the 10 species. The first three columns are cochleagrams measured from the same bird and the second three columns are cochleagrams of the same species but a different bird. The title for each cochleagram is the common bird name followed by the Cornell Lab for Ornithology | Macaulay Library recording catalog ID. The inset text details the time segment where the 2 second exemplar was sampled.
