# Supplementary material for: Human perception of avian biodiversity in naturalistic auditory scenes
Source: PLoS Comput Biol. 2026 Sep 21;22(9):e1014775. doi: 10.1371/journal.pcbi.1014775 (PMC13645227; doi:10.1371/journal.pcbi.1014775)
Supplement: Supplementary file 2 [file pcbi.1014775.s002.pdf]

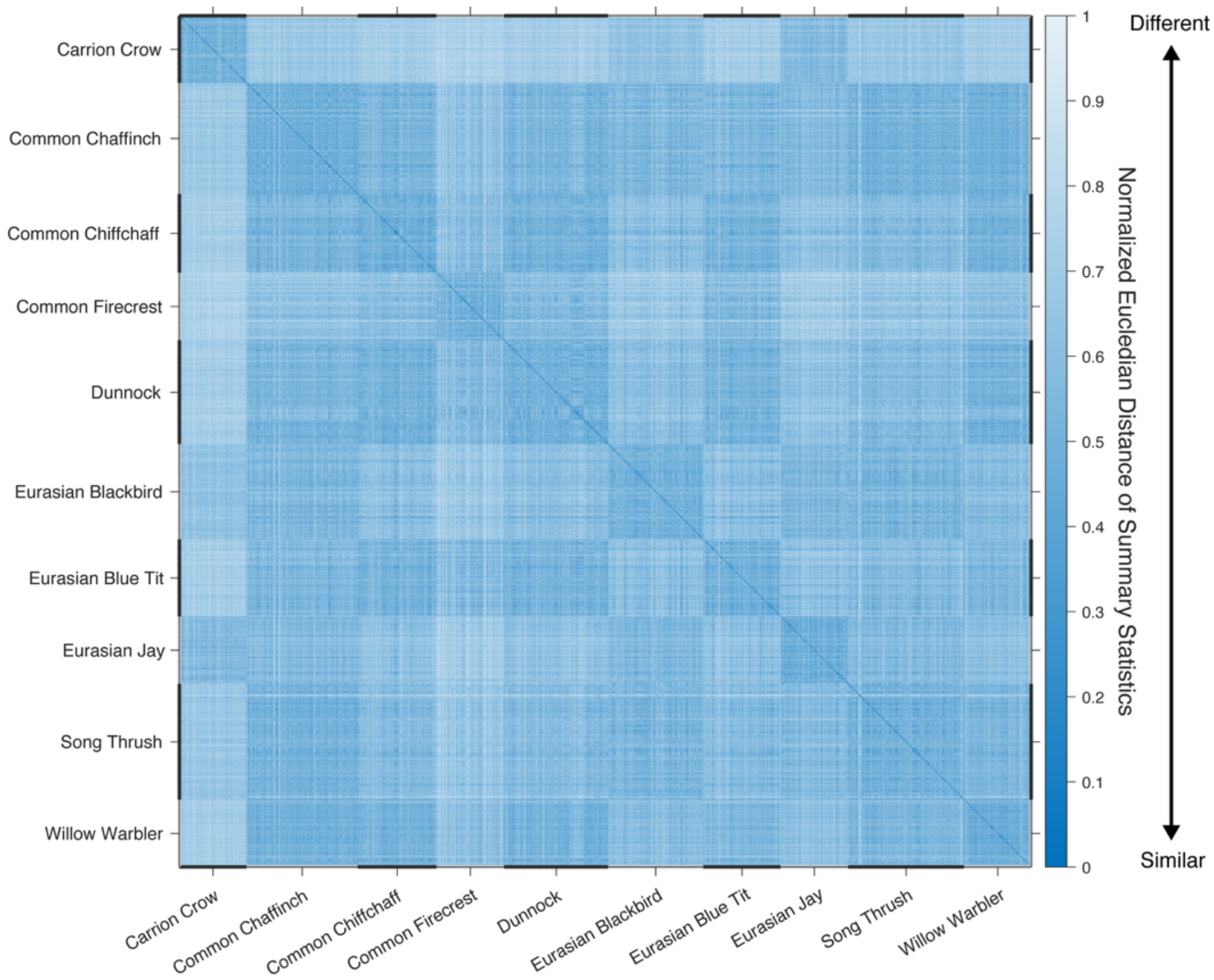

Figure S2 – Normalized Euclidean distance of summary statistics for individual sound pairs of Bird Sound database. The correlation shows both the within-species and across-species similarity. The diagonal has a value of 0 as the distance is computed between identical sound samples. The plot shows the individual sound similarity, whereas the mean within and across species similarity is shown in Figure 1E. The light and dark bands on the perimeter of the plot show the range of sound samples attributed to the species. A different number of sound samples was included for each species, which ranged from 80 to 132 individuals. There were 3 samples per individual, so the final matrix varied for each species from 240 to 396. The total number of sounds samples analyzed was 3039.
