# Supplementary material for: Human perception of avian biodiversity in naturalistic auditory scenes
Source: PLoS Comput Biol. 2026 Sep 21;22(9):e1014775. doi: 10.1371/journal.pcbi.1014775 (PMC13645227; doi:10.1371/journal.pcbi.1014775)
Supplement: Supplementary file 3 [file pcbi.1014775.s003.pdf]

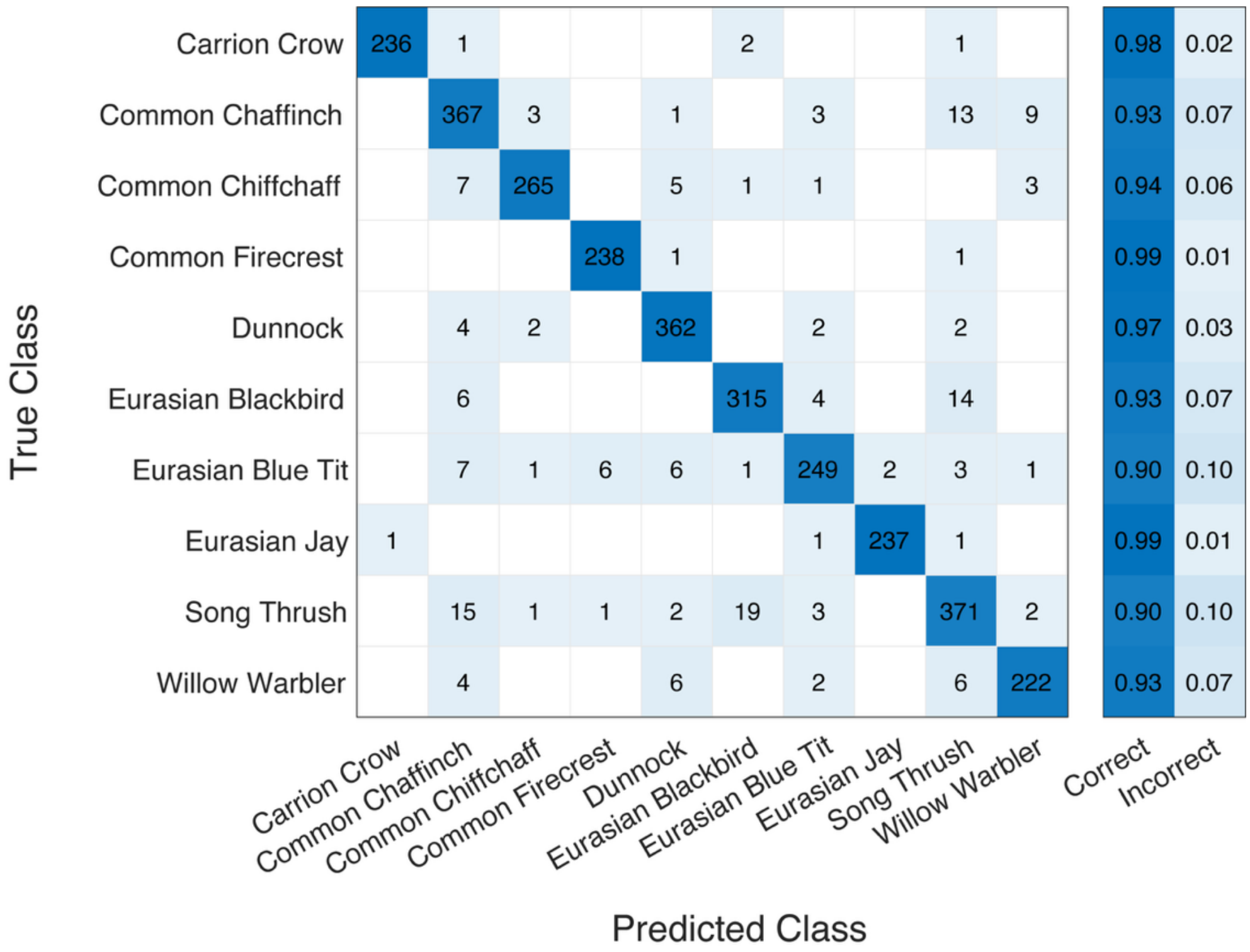

Figure S3 – Multi-class classification of the 10 bird species using an ECOC classifier with boosted decision-tree learners. The classification uses a boosted ensemble of 100 decision trees (Gentle Boost). This ensemble is then used as the learner inside an Error-Correcting Output Codes (ECOC) framework with a one-vs-all approach. The results show the 10-fold cross-validation accuracy of the classifier model. The actual class, or "True Class", is shown on the y-axis and the predicted class is shown on the x-axis. The values on the diagonal show the number of correct classifications, while the off-diagonal numbers indicate the incorrect classifications. The proportion correct for each species is indicated in the right-most two columns labelled "Correct" and "Incorrect". Because there were different numbers of recordings for each species, the total number of classification observations varied. The results suggest that statistics measured from the auditory texture model are sufficient to identify (classify) the bird species with at least 0.90 proportion correct accuracy.
