# Supplementary material for: Human perception of avian biodiversity in naturalistic auditory scenes
Source: PLoS Comput Biol. 2026 Sep 21;22(9):e1014775. doi: 10.1371/journal.pcbi.1014775 (PMC13645227; doi:10.1371/journal.pcbi.1014775)
Supplement: Supplementary file 4 [file pcbi.1014775.s004.pdf]

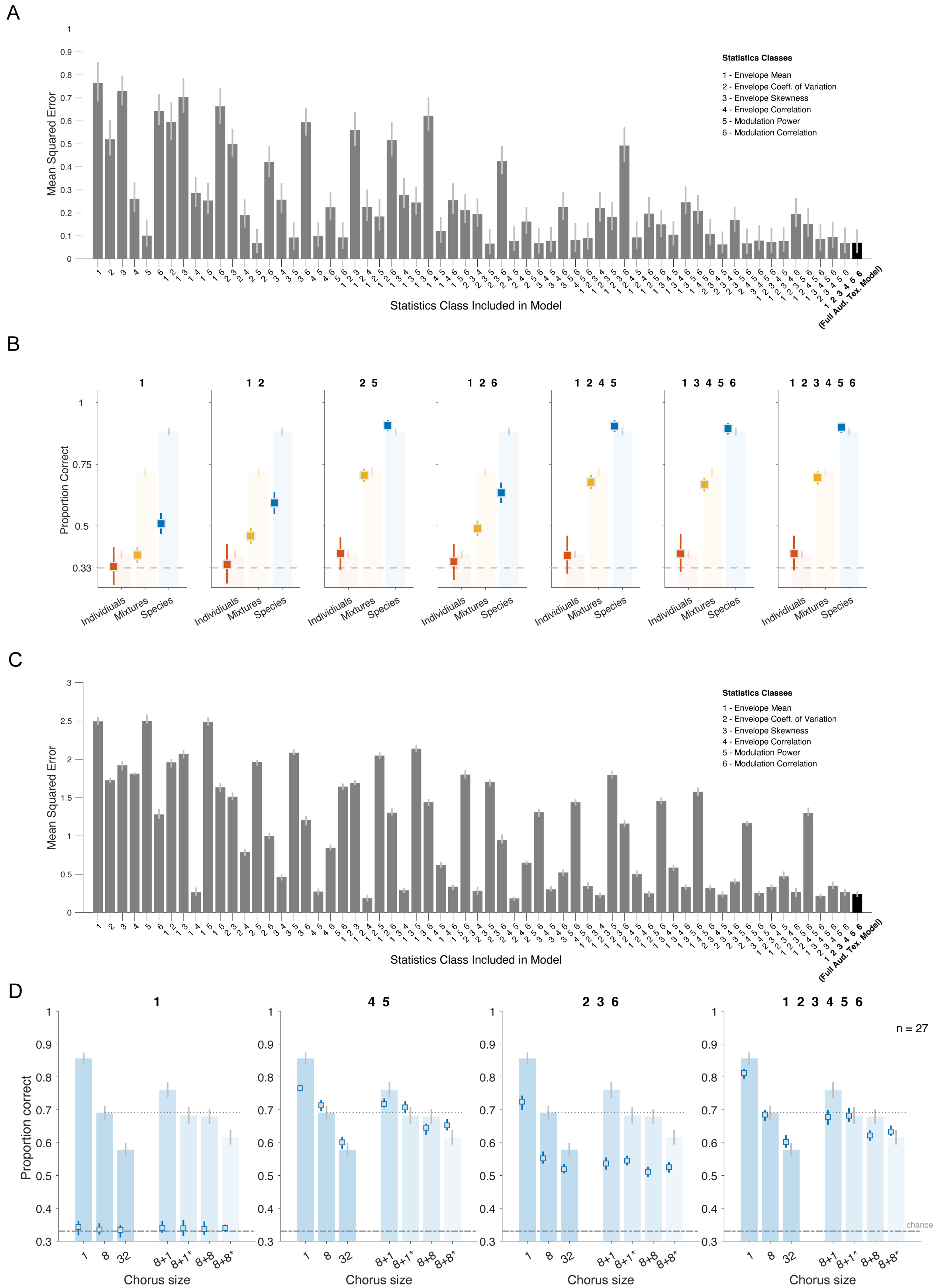

Figure S4 – Ablation of Auditory Texture Model for Species Mixture Discrimination Task (Experiment 4) and Species Mixture Exemplar Discrimination Task (Experiment 5). The auditory texture model was ablated by removing statistics classes. The auditory texture model includes 6 statistics classes: (1) Envelope Mean, (2)

Envelope Coefficient of Variation, (3) Envelope Skewness, (4) Envelope Correlation, (5) Modulation Power, and (6) Modulation Correlation. (A) The plot shows the mean squared error for the 62 ablated models in addition to the complete model for Experiment 4. The statistics class is denoted in the plot legend and corresponds to the x-axis tick mark labels. The x-axis labels describe the statistics classes that are included in the model. The right-most datapoint, which is highlighted in black, is the full auditory texture model. The results show that the model performance varies when the model statistics classes are ablated. Although the ablated models are difficult to differentiate, the Modulation Power appears to play a critical role in capturing the performance variation across experiment conditions. (B) 7 example ablated model results compared to the 3 experiment conditions from Experiment 4. The model with only the Envelope Mean performs poorly relative to the auditory texture model. Ablated models that include the Modulation Power perform better overall, suggesting Modulation Power may be the statistics class the accounts the model performance approaching that of humans. (C) The plot shows the mean squared error for the ablated auditory texture models and the complete model for Experiment 5. The x-axis again denotes the statistics classes included in the model. The right-most datapoint is the full auditory texture model. The results suggest the Envelope Correlation contributes to the performance variation across conditions. (D) 4 example ablated models that vary in performance. The model with Envelope Mean only, appears to preform quite poorly relative to the auditory texture model. The model including the Modulation Power and Envelope Correlation appears to better capture the performance variation across conditions as compared to a model that includes the Envelope Coefficient of Variation, Skewness and Modulation Correlation.
