# Supplementary material for: Human perception of avian biodiversity in naturalistic auditory scenes
Source: PLoS Comput Biol. 2026 Sep 21;22(9):e1014775. doi: 10.1371/journal.pcbi.1014775 (PMC13645227; doi:10.1371/journal.pcbi.1014775)
Supplement: Supplementary file 5 [file pcbi.1014775.s005.pdf]

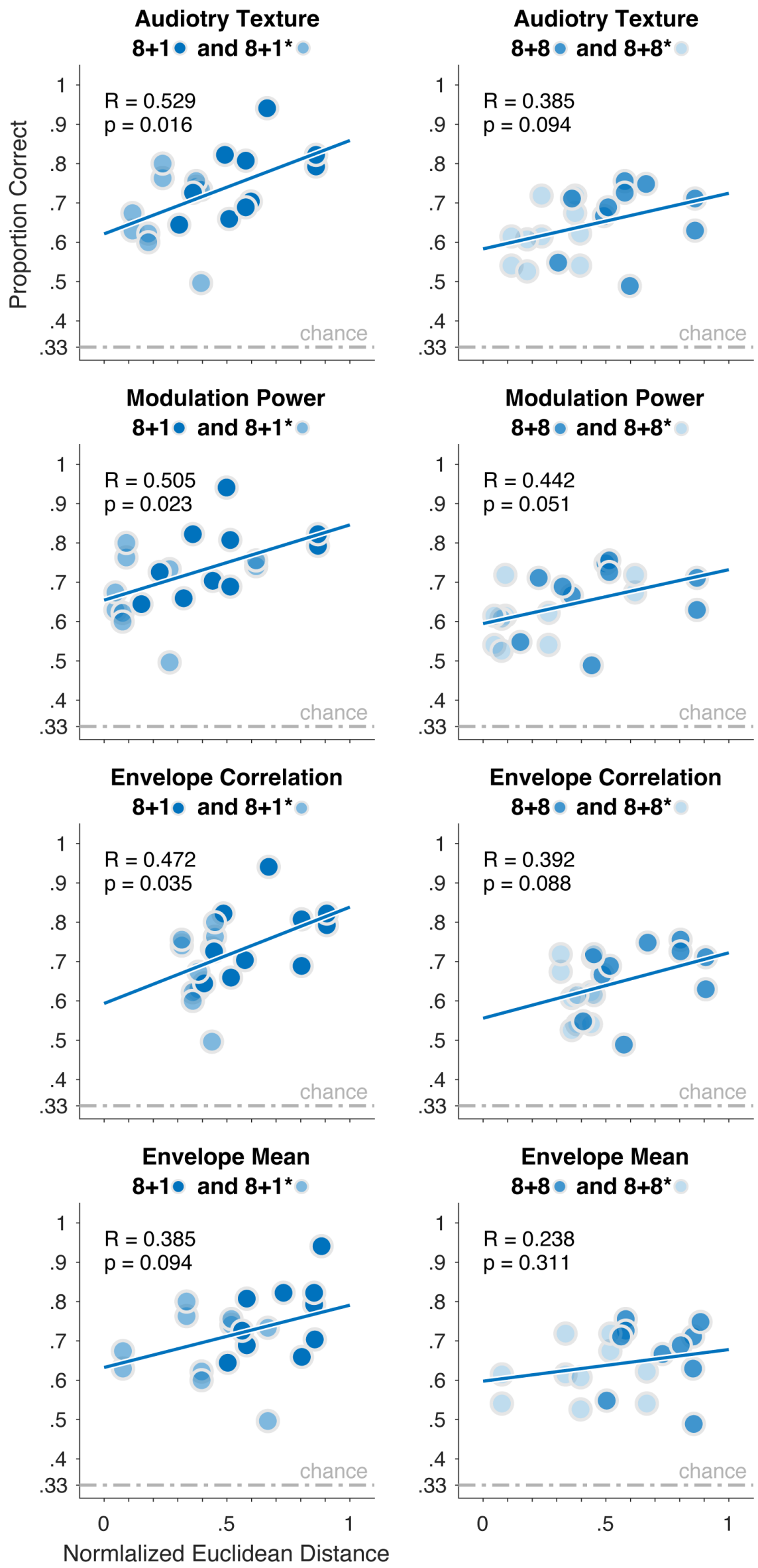

Figure S5 – Scatter plots of the normalized Euclidean distance and human proportion correct (Experiment 5). The four rows correspond to four classes of statistical distance measures: 1) Auditory Texture, 2) Modulation Power, 3) Envelope Correlation, and 4) Envelope Mean. The two rows correspond to paired conditions: 1) 8+1 and 8+1*, and 2) 8+8 and 8+8*. The reported R values are Pearson correlation coefficient and corresponding p values for significance testing. All plots show a positive correlation between the distance measure and human discrimination performance, suggesting the distance between the sound pairs is correlated with discriminability of exemplars. However, the significance of the correlation varies across distance measures. There is a significant correlation for the 8+1 and 8+1* condition for the Auditory Texture, Modulation Power and Envelope correlation, but not for the other conditions and/or distance measures.
