# Supplementary material for: Human perception of avian biodiversity in naturalistic auditory scenes
Source: PLoS Comput Biol. 2026 Sep 21;22(9):e1014775. doi: 10.1371/journal.pcbi.1014775 (PMC13645227; doi:10.1371/journal.pcbi.1014775)
Supplement: Supplementary file 6 [file pcbi.1014775.s008.pdf]

S1 Text

Image citations.

Reference images used in Figure 1C:

Carrion Crow: Carrion Crow 2023 11 19 02.jpg. (2025, October 20). *Wikimedia Commons*. Retrieved March 31, 2026, from https://commons.wikimedia.org/w/index.php?title=File:Carrion_Crow_2023_11_19_02.jpg&oldid=1102544776.

Common Chaffinch: Common chaffinch.jpg. (2026, May 12). *Wikimedia Commons*. Retrieved July 29, 2026, from https://commons.wikimedia.org/w/index.php?title=File:Common_chaffinch.jpg&oldid=1212718991.

Common Chiffchaff: Common Chiffchaff 2025 04 04 01.jpg. (2026, March 19). *Wikimedia Commons*. Retrieved March 31, 2026, from https://commons.wikimedia.org/w/index.php?title=File:Common_Chiffchaff_2025_04_04_01.jpg&oldid=1183629849.

Common Firecrest: Regulus ignicapilla 91940885.jpg. (2026, February 2). *Wikimedia Commons*. Retrieved March 31, 2026, from https://commons.wikimedia.org/w/index.php?title=File:Regulus_ignicapilla_91940885.jpg&oldid=1158395383.

Dunnock: Dunnock (Prunella modularis) 2021.jpg. (2026, May 16). *Wikimedia Commons*. Retrieved July 29, 2026, from https://commons.wikimedia.org/w/index.php?title=File:Dunnock_(Prunella_modularis)_2021.jpg&oldid=1215125906.

Eurasian Blackbird: Turdus merula 1308.jpg. (2025, March 5). *Wikimedia Commons*. Retrieved March 31, 2026, from https://commons.wikimedia.org/w/index.php?title=File:Turdus_merula_1308.jpg&oldid=1005735011.

Eurasian Blue Tit: Eurasian Blue Tit 2025 04 07.jpg. (2026, June 20). *Wikimedia Commons*. Retrieved July 29, 2026, from https://commons.wikimedia.org/w/index.php?title=File:Eurasian_Blue_Tit_2025_04_07.jpg&oldid=1234679390.

Eurasian Jay: Garrulus glandarius 1 Luc Viatour.jpg. (2025, November 22). *Wikimedia Commons*. Retrieved March 31, 2026, from https://commons.wikimedia.org/w/index.php?title=File:Garrulus_glandarius_1_Luc_Viatour.jpg&oldid=1119613450.

Song Thrush: Song Thrush (Turdus philomelos).jpg. (2026, March 13). *Wikimedia Commons*. Retrieved March 31, 2026, from https://commons.wikimedia.org/w/index.php?title=File:Song_Thrush_(Turdus_philomelos).jpg&oldid=1180140858.

Willow Warbler: Willow Warbler - Phylloscopus trochilus2.jpg. (2026, April 13). *Wikimedia Commons*. Retrieved July 29, 2026, from https://commons.wikimedia.org/w/index.php?title=File:Willow_Warbler_-_Phylloscopus_trochilus2.jpg&oldid=1196888908.

Center (circular) image: Chemin Jean Racine 2017-06 n1.jpg. (2025, November 26). *Wikimedia Commons*. Retrieved July 29, 2026, from https://commons.wikimedia.org/w/index.php?title=File:Chemin_Jean_Racine_2017-06_n1.jpg&oldid=1121704868.
