# Supplementary material for: Human perception of avian biodiversity in naturalistic auditory scenes
Source: PLoS Comput Biol. 2026 Sep 21;22(9):e1014775. doi: 10.1371/journal.pcbi.1014775 (PMC13645227; doi:10.1371/journal.pcbi.1014775)
Supplement: Supplementary file 7 [file pcbi.1014775.s009.pdf]

Summary

In this work, the authors investigate how human listeners perceive biodiversity in acoustic scenes composed of bird vocalizations, focusing on two components: species abundance and species richness. They construct a curated database of bird calls and synthesize bird choruses by summing different numbers of individuals and species. Across a series of oddity-discrimination experiments, they ask: (i) how well listeners can discriminate differences in chorus size within a species; (ii) how they discriminate between species and between mixtures of species; and (iii) how additional background sounds affect performance. They complement the behavioral data with several observer models, including an auditory texture model and simpler cue-based models. The findings are interpreted as evidence that large bird choruses are processed as auditory textures, represented via summary statistics, and that species mixtures are grouped or segregated depending on disparities in these statistics.

Overall, this is a carefully executed and thoughtfully framed study that is a natural next step to previous ecoacoustics studies from the authors' research group. It extends earlier work on perceived acoustic biodiversity and natural soundscape discrimination by:

- Switching from subjective numerosity judgments to objective oddity discrimination
- More cleanly manipulating abundance vs richness with synthetic choruses
- Leveraging auditory texture models and cue-based observers to interpret the behavioral data mechanistically

The broad conceptual story is plausible and appealing. The stimulus construction appears careful, the experimental paradigms are appropriate, and the modeling is a welcome addition. At the same time, some of the central interpretive claims are stated more strongly than the current analyses justify. In particular, the texture model is not clearly shown to be uniquely required relative to simpler, long-term cues, and the role of explicit streaming versus global scene statistics in mixtures remains somewhat qualitative. The ecoacoustic implications could also be more carefully delimited given the artificial nature of the stimuli.

I believe the manuscript would benefit substantially from a more quantitative comparison of models, clearer framing of the evidence for texture and streaming, and a more nuanced discussion of ecological validity and the relationship to previous work from the same group.

Major comments

1. Specificity of texture perception. The behavioral and modeling results are nicely consistent with a summary-statistics / texture interpretation, especially for dense

choruses. However, in a few places (e.g. the abstract and Discussion) the wording suggests that large choruses are definitively "perceived as textures" or "are perceived by their time-averaged statistics".

Given that simpler long-term cues (e.g. spectrum, modulation power) appear to account for much of the observed pattern in performance, I would suggest slightly softening these formulations (e.g. "consistent with a texture-like summary-statistics representation") to better reflect the level of constraint provided by the current data. Cross-validated prediction accuracy or some formal measure (AIC/BIC, deviance) could help show whether the texture model adds explanatory value beyond simpler statistics. If it does not, the interpretive claims should be softened accordingly.

2. Quantitative delineation of streaming vs global statistics. The mixture experiments (especially Exp. 4–5) provide intriguing evidence that performance depends on which species are combined, consistent with the idea that listeners sometimes treat mixtures as a single fused source and sometimes access species-specific information. However, the current modeling mainly shows that a purely global texture representation is insufficient and that a fully segregated "oracle" also does not match human data, rather than precisely pinning down when and how streaming occurs. I would suggest slightly softening categorical statements such as "listeners group mixtures when their statistics are similar but segregate species when their statistics are different," and, if possible, adding a brief quantitative analysis linking a continuous measure of acoustic similarity (e.g., distances in spectral/modulation/texture spaces) to performance across mixture conditions to more concretely support the role of similarity in promoting grouping vs segregation.

3. Ecological validity and conclusion scope. The manuscript is framed in terms of natural parks, temperate forests, and biodiversity monitoring. However, the choruses used here are strongly artificial: up to 32 individuals of the same species, fully overlapping, no spatialization, no explicit modeling of species-specific call timing, and a relatively clean listening context compared to real forests.

This is not a criticism of the experimental design per se: controlled synthetic stimuli are appropriate for mechanistic psychophysics. It does, however, limit how directly the results can be generalized to real soundscapes and field-based ecoacoustic monitoring.

The authors have already mentioned most, if not all, of the limitations I have described above individually in the Discussion section, but I suggest that they more explicitly acknowledge these limitations and define the ecoacoustic implications as mechanistic constraints on how humans might perceive abundance and richness, rather than as a direct account of human perception of actual natural choruses.

It may also be useful to briefly relate these findings to the known limitations of common acoustic indices (which often correlate only moderately and inconsistently with biodiversity in real recordings), emphasizing that human perception relies on richer scene-analysis mechanisms than those indices capture.

4. Positioning relative to previous work. This study sits naturally alongside earlier work from the same research group on perceived acoustic biodiversity and natural soundscape perception. However, the manuscript currently does not fully spell out what is new relative to, for example, previous numerosity and soundscape discrimination studies like Grinfeder, et al. (2025).

I recommend adding a short paragraph in the Introduction and/or Discussion that explicitly summarizes how this work extends and complements those studies: use of objective oddity discrimination, cleaner manipulation of abundance vs richness in synthetic choruses, explicit texture/observer modeling, etc. This will help readers, especially those outside this subfield, understand the incremental conceptual advance.

5. Statistical methodology. The behavioral results are mostly presented as percent correct per condition, analyzed with ANOVAs and multiple t-tests. This is understandable given the design, but it is not the most robust approach for complex, factorial psychophysical data.

A mixed-effects logistic regression model (or other appropriate GLMMs) in this case would be more robust and make use of the within-participant performance variability. The model would for example have fixed effects for the main experimental factors (chorus size, species, mixture type, presence of background, etc.), and random effects for subjects.

I am not asking the authors to redo all their analyses using mixed-effects models, but doing so would make the statistical basis of the findings stronger. I would therefore suggest that the authors apply such a model to at least the main experiment and report the results, for example in the supplementary materials.

6. Multiple comparisons. Considering the multiple comparisons performed in comparison groups, the authors should explicitly address how multiple comparisons were handled (e.g., correction methods or pre-specified contrasts). For the larger effect sizes this does not make a difference, but for example in experiment 5 (comparing the single-species chorus of 8 birds to the 8+1, 8+1*, 8+8, 8+8* mixtures) where some p-values are only just below 0.05, a correction for multiple comparisons can make these contrasts statistically non-significant.

Minor comments

1. Participant description. The text would benefit from a brief, explicit description of participant demographics and experience beyond age and gender (e.g., hearing status, any exclusion criteria, and musical or ornithological background). Even if all participants were naive students, stating this clearly would help assess generalizability.

2. Code availability. At the time of review, the code does not appear to be accessible at the specified URL (likely because the repository is still private). It would be helpful for reviewers to have access to the code before final publication.

3. Typos and clarity. There are a few typos in the manuscript, but one specifically makes the sentence unreadable. The sentence at line 77 is missing a word: I think it was meant to be “Second, variation in the spectral and temporal characteristics of bird vocalizations…”. That paragraph is generally hard to read and could use some rephrasing for clarity.
