# Supplementary material for: Human perception of avian biodiversity in naturalistic auditory scenes
Source: PLoS Comput Biol. 2026 Sep 21;22(9):e1014775. doi: 10.1371/journal.pcbi.1014775 (PMC13645227; doi:10.1371/journal.pcbi.1014775)
Supplement: Supplementary file 8 [file pcbi.1014775.s010.pdf]

We would like to sincerely thank the three reviewers for their thoughtful and thorough comments and feedback. We have tried our best to incorporate all of the suggestions into the paper and answer the questions with convincing and well supported responses. We feel that all of the reviewers raised important questions and concerns about the study and the updated version of the paper is much improved, with stronger, evidence-based conclusions.

The questions/comments raised by the three reviewers resulted in updates to the manuscript text, additional analyses, and new figures. The six major updates include:

1. We implemented a new classifier (decision tree / one-versus-all / ECOC) to reveal similarity of bird species. The one-versus-all decision tree was used as we have multiple classes (i.e. 10 species). This analysis was intended to make clear the relationship between the 10 bird species. The classifier operated on the statistics measured from the auditory texture model. The classifier results were meant to complement the Euclidean distance metric used to characterize the similarity between species (e.g. Figure 1E).

   Using a k-fold analysis, we found that the within species classification accuracy was high (above 90%) but varied across species. The confusions also mirrored those measured from the Euclidean distance metric and were therefore congruent with the similar/different species mixtures used in experiment 5.

   The classifier used all the statistics measured using the auditory texture model, which includes approximately 1500 parameters. This is quite high relative to the roughly 3000 sounds that comprise our Bird Sound Database. However, a classification using subsets of the texture statistics classes yielded comparable classification accuracy.

   The resulting analysis is provided in Supporting Information S1: Figure 3 as well as the inclusion of the classification description in the methods section.

   Thank you to Reviewer #1 for the thoughtful suggestion.

2. In order to tease apart how each of the statistics classes of the auditory texture model contributed to the model performance, we ran an ablation analysis of the model for Experiment 4b (Individuals/Species/Mixtures discrimination), and Experiment 5 (More/Less similar species exemplar discrimination task). The model was partitioned into 6 classes: (1) Envelope Mean, (2) Envelope Coefficient of Variation, (3) Envelope Skewness, (4) Envelope Correlation, (5) Modulation Power, and (6) Modulation Correlation. Each statistics class contributed equally to the model decision. The model parameters, namely the internal noise, was held constant for all model ablations.

The model ablations were applied in all possible configurations of the 6 statistics classes, resulting in 62 ablated models and the full auditory texture model (63 models in total).

The ablated models varied in similarity to human performance, which is shown in Supporting Information S1: Figure 4.

For experiment 4b, the results are shown as a mean squared error (mse) between the model proportion correct and human proportion correct for the 3 experiment conditions (Individuals/Mixture/Species). In addition to the mse, 7 ablated models are shown with the human performance (bars) and model performance (squares).

For experiment 5, the results are shown as a mean square error (mse) between the model and the human performance for the 7 model conditions. In addition to the mse, 4 ablated models are shown to exemplify the variation in model performance across ablations.

Thank you again to Reviewer #1 for the valuable suggestion.

3. The original manuscript makes overstated claims about the contributions of segregation/integration/ masking. To address this issue, we have attempted to revise the abstract, results and discussion sections to be more in-line with the behavioral and modeling results.

   In addition, we added an additional model analysis of experiment 6. The experiment compared exemplar discrimination performance across a range of mixture conditions including foreground and background mixtures. The behavioral results showed listeners were minimally affected by the introduction of background sounds relative to the foreground only conditions.

   The model performance qualitatively replicated human performance for the foreground only conditions of bird choruses of size 1, 8 and 32. However, the model deviated from human performance for mixtures of small choruses with background noise. The model, in these cases, performs worse that humans. Lastly, the model performance for large chorus mixtures, and the background only condition are more comparable.

   These modeling results do not directly disentangle the question of segregation, integration and masking. However, the model results are in-line with those of experiment 5, where performance seemed to be modulated by the disparities in the mixture sounds summary statistics (i.e. performance was higher when the mixture

statistics were different, and lower when the mixture statistics were similar). Because the background sound had statistics that were quite different from the bird species (as shown by Figure 6b, and 6c), the performance of the foreground+background is similar to that of the 8+1 and 8+8 conditions of experiment 5.

Thank you very much to Reviewer #2 for bringing this important issue to the forefront.

4. The statistical analysis for the majority of the behavioral experiments was conducted via ANOVA or t-test. This approach was quite simple, and given the experiments can often include many conditions and stimuli, additional analyses may better expose the significance of experiment parameters. We therefore ran a mixed-effects logistic regression analysis on main experiment (Experiment 1a/b) to better handle the multi-factorial experiment design.

   The model equation had the chorus size and species as fixed effects and the participants were random effects. The chorus size had 2 values, as the experiment was a comparison between intervals of sound that were composed of different sized choruses (in the figure they are written as N and M).

   The results show that there is a significant effect of chorus size. In addition, there is a significant effect of species. This was somewhat unexpected result, but perhaps the species vary in the acoustic properties, and hence the way the individual vocalizations combine, which results in differences in discriminability.

   Lastly, the participants (random effects) are non-significant except for 1 participant. Which suggests that the main effect driving the discriminability is chorus size and species.

   Thank you to reviewer #3 for bringing up this important issue.

5. Lastly, the analysis of Experiment 5 included multiple comparisons which was not corrected for in the analysis. The analysis compared 5 conditions, including a chorus of 8 with 4 mixture conditions (8+1, 8+1*, 8+8, and 8+8*). The original analysis included a repeated measures ANOVA. The updated analysis includes a Bonferroni correction for multiple comparisons.

   We rerant the analysis as a repeated measures ANOVA corrected (Bonferroni) for multiple comparisons. Indeed, for the original data (N=20), a key condition moved

from being significant to non-significant. Specifically, the comparison between 8+8 and 8+8* conditions no longer showed a significant difference.

To address this issue, we collected another 7 participants to raise the total number of participants for Experiment 5 to N=27.

The main manuscript text has been updated with the new significance values. In addition, the caption for Experiment 5 (Figure 6) has been updated to describe the statistical analysis conducted. Lastly, the methods section for Experiment 5 now includes additional details about the statistical analysis, as well as the significance values for the main experiment conditions.

Thank you again to reviewer #3 for identifying this shortcoming in the analysis.

6. The results of experiment 5 show that exemplar discrimination performance is modulated by the disparity of the mixture sounds summary statistics. However, the results are plotted as averages across two broad categories (i.e. most similar and most different). This raises the question of whether the discriminability is indeed a function of the disparity of the summary statistics.

   In an attempt to address this question, we added a supplemental figure that shows a continuous measure of the acoustic similarity and performance across mixture conditions. The measure of acoustic similarity is performed for the spectrum (envelope mean), envelope correlation, modulation power, auditory texture spaces. The figure plots the paired conditions, 8+1 with 8+1* and 8+8 and 8+8*.

   The expectation is that if performance is indeed modulated by the disparity in summary statics, human exemplar discrimination performance should be positively correlated with distance. To put it clearly, if the distance is small, discrimination performance should be lower, and if the distance is large, discrimination performance should be higher.

   Indeed, we find a positive correlation between the distance and performance. However, not all distance measures and conditions yielded a significant correlation. For the auditory texture space, there is a significant positive correlation for the auditory texture model 8+1 with 8+1* conditions ($R=0.529$, $p=0.016$). However, the correlation is not significant for the 8+8 and 8+8* condition ($R=0.385$, $p=0.094$). Interesting, the correlation and significance values seem to vary based on the measured space, with the spectrum (envelope mean) yielding the lowest correlation values.

   We would like to thank Reviewer #3 from bring up this important point.

Thank you again to the three reviewers for the time put towards reviewing the manuscript and their thorough and thoughtful comments. We believe the manuscript has improved quite a lot because of the helpful and clear comments.

Below you can find responses to each individual comment.

Thank you again.

Kind regards

Richard McWalter and Professor Christian Lorenzi

Reviewer #1:

Pros:

The authors present a solid human psychoacoustics study focusing on bird vocalization perception with implications for auditory scene analysis. Using a battery of discrimination tasks, they demonstrate that humans can perceive differences in both the number of individuals (abundance) and the diversity of species (richness) within complex natural mixtures. This sensitivity declines once the chorus exceeds about 8 individuals, at which point the sounds are perceived as integrated auditory textures. Through behavioral experiments and computational modeling, the authors show that listeners use summary statistics rather than detailed temporal features to represent large mixtures. I appreciated the use of natural sounds in a psychophysics experiment, which isn't done as much as it should be.

Thank you for the positive comments.

Cons:

The manuscript text and clarity could be improved. I have noted areas for improvement + typos below. Although this work fills a necessary gap in the auditory psychoacoustics field, I worry the scope might be a little narrow for a general audience.

Thank you for your feedback. We have attempted to address all of the comments below.

For broader appeal, the authors could analyze the interpretability of the sound texture observer model, e.g. through ablations to identify which auditory features drive performance.

We have run the auditory texture model with all possible statistics class ablations. The result was quite informative, and pointed to the Envelope Correlation as providing a main cue a selection of the study experiments.

The model was partitioned into 6 classes: (1) Envelope Mean, (2) Envelope Coefficient of Variation, (3) Envelope Skewness, (4) Envelope Correlation, (5) Modulation Power, and (6) Modulation Correlation. Each statistics class contributed equally to the model decision. The model parameters, namely the internal noise, was held constant for all model ablations.

The model ablations were applied in all possible configurations of the 6 statistics classes, resulting in 62 ablated models and the full auditory texture model (63 models in total).

The ablated models varied in similarity to human performance, which is shown in Supporting Information S1: Figure 4.

For experiment 4b, the results are shown as a mean squared error (mse) between the model proportion correct and human proportion correct for the 3 experiment conditions (Individuals/Mixture/Species). In addition to the mse, 7 ablated models are shown with the human performance (bars) and model performance (squares).

For experiment 5, the results are shown as a mean square error (mse) between the model and the human performance for the 7 model conditions. In addition to the mse, 4 ablated models are shown to exemplify the variation in model performance across ablations.

They might also compare human texture models with bird-inspired or avian auditory models (if available) to test whether birds outperform humans, adding a valuable comparative dimension to the study.

This comparative approach was illustrated in a short modelling attempt conducted by Lorenzi et al. (Trends in Hearing, 2023 ; see Fig 7), where mid-level auditory representations (i.e., output of the modulation filterbank model, Dau J Acoust Soc Am 1997) were computed in response to natural soundscapes recorded in a variety of pristine habitats (tropical, temperate forests, savannah, desert) using a computational model of the auditory system tuned to either human or Zebra-Finch parameters (such as listening bandwidth (absolute thresholds), frequency selectivity, modulation-frequency selectivity and temporal resolution). These mid-level auditory representations (see Fig. 7 in Lorenzi et al., 2023) clearly show that natural soundscapes differing in biodiversity (the more equatorial habitats being the most biodiverse) should be easily discriminated based on perceptually-relevant spectro-temporal cues for both humans and Zebra Finches, although availability of spectro-temporal cues is reduced for Zebra finch. Conducting a similar study with the specific bird species inhabiting Vallée de Chevreuse would be interesting. Unfortunately, information about absolute thresholds, frequency selectivity, modulation-frequency selectivity and temporal resolution is lacking for the species under consideration, making this endeavor currently unfeasable. Such a research program would require a different design where the recorded bird species would be simultaneously characterized/screened through neuro-ethological/bioacoustic methods.

Comments:

Introduction

Line 33: This sentence is hard to follow - structure is a bit off and is a run-on. Consider splitting and/or rephrasing.

Thank you for highlighting this run-on sentence. We have split the sentence and tried to use parallel language to make it easier to follow.

Line 40: This sentence is hard to follow - structure is a bit off and is a run-on. Consider splitting and/or rephrasing.

Thank you again. We have revised the sentence and split it into two parts. The first sentence focuses on the stimuli and the second the environment which they most frequently occur.

Line 49-52: Which specific listening conditions are the authors referring to? The ecological-validity of the study could use much better framing here.

We have removed the identified sentence and replaced it with improving framing related to ecological validity.

Line 54-56: This sentence is hard to follow - structure is a bit off and is a run-on. Consider splitting and/or rephrasing.

Thank you. This sentence has been broken into two sentences, and some superfluous detail has been removed.

Line 59-61: I like this sentence a lot and it helps make the focus quite clear.

Thank you for the positive comment.

Line 62: Remove "The" to start the sentence.

Corrected. Thank you.

Line 66-67: I think a word or two is missing after "sound recordings,".

Thank you. This has been corrected to "soundscape field recordings".

Line 108-110: It would be more appropriate to pose the questions as hypotheses with testable predictions, rather than listing them.

Thank you. We have reformulated the list of questions as hypotheses with testable predictions. We state what we expect to occur for the different experiment conditions, both in terms of biodiversity perception and species mixtures, but highlight that we do not yet know the limits of this perception in human listeners.

General: Introduction contains many sentences that are difficult to follow (marked above). Please consider splitting/rephrasing these sentences.

We have attempted to address the deficiencies in the introduction section. We believe the reformulated introduction offers the reader and improved experience.

Moreover, the framing around perception of bird species could be improved. The authors mention ecological validity of being able to detect different species, which could be expanded to address this concern. Why is it advantageous for humans to be able to segregate abundance and richness of birds specifically?

Birds are ubiquitous and make a unique contribution to natural soundscapes (for a review, see Lorenzi et al., Trends in Hearing, 2023 ; Miller-Viacava et al., J. Acoust. Soc. Am., 2025). Assessment of abundance and species richness may have played a role in the *habitat selection behaviour* of our Homo Sapiens ancestors (Doligez & Boulinier, 2008; Farina et al., 2021; Mullet et al., 2017), and it is possible that this auditory ability plays a role in the restorative effects (Buxton et al., 2021) and impact on mental health of people (Buxton et al., 2024) previously documented for people exposed *to* natural settings.

I like the focus on natural sound processing generally, but the experiments end up focusing specifically on birds, whose findings are not necessarily generalizable to other natural sounds.

As indicated above, birds are ubiquitous and make a unique contribution to natural soundscapes (cf. Lorenzi et al., Trends in Hearing, 2023 ; Miller-Viacava et al., J. Acoust. Soc. Am., 2025). For instance, annotations of large soundscapes databases indicate that birds participate the most to natural soundscapes across a wide variety of terrestrial biomes (Chen et al., 2021; Divyapriya & Pramod, 2019; Gasc, Anso et al., 2018; Gasc, Gottesman, 2018; Mullet et al., 2016; Phillips et al., 2018). Given that bird sounds are quite different spectrally and temporally from insect, anuran and mammal sounds (Aubin & Bremond, 1983; Capranica et al., 1985; Catchpole & Slater, 2008; Fonseca, 2014; Gerhardt & Bee, 2007; Stein, 1968; Sueur & Aubin, 2003), this overrepresentation of birds imposes specific acoustic regularities (that is, unique statistical structures) to natural soundscapes, to which our human ancestors have been exposed for at least several million years (Senter, 2008).

Results

Figure 1C

-Add time/frequency axes to one of the spectrograms please (if all the same time/frequency). If not, I recommend adding a time scale bar and frequency min/max for each example.

Thank you. I have added time/frequency axis to one of the spectrograms. They are indeed all the same. The frequency (vertical) axis spans 50 to 10kHz and the time (horizontal) axis is 2 seconds.

-What kind of pre-processing was done to the spectrograms? For example, the Dunnock spectrogram has increased noise around each syllable of the song, as if the rest of the recording has been denoised (same for Willow Warbler and Eurasian Blue Tit).

We did apply denoising to the sounds in order to achieve a rough baseline signal to noise level across the sound database. A description of the denoising procedure has been added to the methods section (Stimulus selection: Bird sound database).

The denoising was performed on a sound-by-sound basis with Adobe Audition, using the noise reduction process. The goal was not to remove all of the noise, but to make the signal to noise ratio of the sounds more homogenous across the entire database. Due to the crowdsourced nature of the audio recordings used for experimental stimuli, there was considerable variability recording quality, such as signal to noise ratio, extraneous sound events, reverberation, and audio distortions/non-linearities. To create our database, we began with 300 recordings of each species with the goal of finding 80 that were suitable for use in our experiments (see methods and models: stimulus selection – bird sound database). However, there was indeed some variability in the quality of the recordings. This is acknowledged and highlighted in the results section, methods section as well as Supplemental Figure 1 and 2, which includes example spectrograms for different species, individuals, and excerpts.

There was limited documentation as to what pre- or post-processing might have been applied to the audio recordings before being uploaded to the database. Some sounds did have information about the recorder and microphone equipment, but this was sparsely reported. As such, it may be the case that recordings were filtered/altered with a denoising algorithm or filtering prior to uploading. However, the Macaulay library webpage suggests that advises uploaders to minimize any post-processing applied to the recorded sounds (see https://support.ebird.org/en/support/solutions/articles/48001064341 for details).

We must admit that given the variability of the number of sounds available, it was difficult to find enough quality recordings for all 10 species used in the experiments. However, the relative similarity to audio recordings within species (Supplemental Figure 2), the new Random Forest classification results showing high accuracy for species classification with kfold method (thank you for suggesting this addition to the manuscript!), as well as the

behavioral results showing listeners could discriminate between chorus sizes and species, and, all together, we feel these steps ensured that the quality of our Bird Sound Database was sufficient to answer our questions of the perception of biodiversity in humans.

Line 139: Can the authors comment on how they (or Cornell) confirmed that vocalizations were emitted by individual birds? Was there visual or quantitative confirmation?

This was done by the authors. The first step was the label adjacent 3s excerpts of the recordings with the BirdNet ID software. The authors chose the segments from the recording that had the highest single species label confidence. Next, we listened to the top 3 segments (those with the highest ID confidence) and removed sounds that contained additional species/bird vocalizations or significant levels of distortion (background noise, reverberation, anthrophony, recording equipment noise).

The procedure is outlined in the Methods section: Bird Sound Database.

Figure 1E

-It seems like many species can be confused for each other. e.g. Common Chaffinch vs. Willow Warbler/Common Chiffchaff. Instead of Euclidean distance, can you perform a decoding analysis (perhaps SVM or Random Forest trained on features from the auditory model) and plot a confusion matrix? I think this would be more interpretable than the distance metric.

Thank you for the valuable suggestion. We have performed the decoding analysis using a Random Forest classifier. We implemented a one-vs-all approach to account for the multiple classes (10 bird species). The classifier was trained using the sound texture statistics outlined in the methods section. We performed a kfold analysis to obtain accuracy for each species. The confusion matrix plots the percent correct for each species, along with a summary of each species, with the percentage correct.

The sound texture statistics appear to be sufficient to capture the differences across bird species vocalizations. The kfold test accuracy was ~90% or greater for all bird species. Additionally, the “confusions” seem to align with the distance measure. However, we did not perform an explicit comparison to verify.

Supplementary Figure 1

-The within-bird recordings are truncated at different phases of their respective songs. I imagine this will affect how similar they are perceived?

Yes, there is variation in the position of the vocalization within a segment. This wasn't considered specifically. When selecting the segments form the longer recording, we measured adjacent 3s segments and used the BirdNet sound identification tool to identify the sound. We selected the 3 segments form each recording that had the highest single-species (target) identification confidence.

It may have been better to randomly sample overlapping 3 second segments and select those that had the highest species label confidence. However, this was not what occurred.

It should be noted, that after the use of BirdNet, the authors, somewhat painstakingly, evaluated all of the recordings many times in order to "weed-out" samples that deviated from the reference recordings provided by the MNHN in Paris.

Figure 2D

-It surprises me that within species discrimination (i.e. individual recognition) isn't higher for n=m=1. Do the authors show any acoustic quantification of within-species differences that might help explain this? Also, are these statistics collapsed across all species? It would be interesting to see what the curve looks like separated by species.

There is indeed variability across within-species segments. This is best highlighted by the difference (distance) in texture statistics of the individual sound sources presented in Supporting Information S1 Figure 2. Two things are most striking in this figure. The first was that there appears to be some "boundaries" when comparing the individual sound segments, which can be seen by the larger checker-like pattern of the figure. However, second, it is also evident that the distance isn't homogenous for the within-species case, as observed by the variation in luminance/color variation from blue to white within each species block.

Figure 3A (legend): "trail" should be "trial".

Corrected. Thank you.

Figure 3: what is the significance of .05s as the "short" trial?

This was a duration used in previous studies investigating exemplar discrimination (McDermott et. al 2013). In addition, it is perhaps quite short, even in terms of basic auditory processing mechanisms. For instance, loudness perception is thought to be governed by integrating sounds over hundreds of milliseconds.

FIgure 3D

-How do you go about selecting the 0.05s exemplar? Figure 1C show substantial within-song spectrotemporal variation. Therefore, some segments from the song (e.g. the ones with high variation) would be easily perceivable as species specific. Moreover, S1 shows that individual exemplars vary in time, where some examples contain an entire song, while others contain truncated versions of the song. Further elaboration is desirable for: 1.) how exemplars were chosen and 2.) whether the aforementioned potential confounders affect interpretation of results

Thank you for this thoughtful question. We selected the short segments (0.05s) to have the same time-averaged spectrum as the longer (2s) segments. We first measured the time-averaged spectrum using the Auditory Texture model. We then measured the spectrum from random 0.05s segments sampled from the longer 2s segment. We then measured the distance of the spectrum between the short segments and the longer segment, and selected the short segment that had the most similar spectrum (smallest distance). This would ensure the short segments had similar level and frequency content comopared to the longer segments. Indeed, if we simply sampled the short 0.05s segment randomly from the 2s segment, there is a chance the short segment could have little or no sound energy.

We have added text in the Methods Section: Experiment 3 that describes the selection of the shorter 50ms segments.

The variation in signal within a 2 second segment is indeed present. This is, for better or worse, the issue with working with natural sounds. However, we think the main findings of experiment 3 are valid. One might expect that if the variability across segments was minimized, discriminability would still depend on the duration, with the exemplar discrimination decreasing with duration and the species discrimination increasing with duration. However, performance may vary depending on the homogeneity of the original samples (performance might higher for more homogeneous original sounds and lower for more variable natural sounds). This is indeed a very thought-provoking question, and one the authors battled with throughout the project.

Figure 4A (and throughout the manuscript)

-It’s difficult to appreciate the difference in silhouette between species in 4A. Perhaps color code (or have use a unique opacity for) each species?

Thank you for this observation. Indeed, with the number of experiments and conditions it was difficult to differentiate all of the figures with schematics throughout the paper.

We have revised all of the experiment schematics. There are two silhouettes, which signify different species. We then use different colors/shading to differentiate different individuals from the same species.

For instance, in Figure 4A shows birds from the same species as the same color and silhouette and birds of a different species as a different silhouette and color. Specifically, for the *species discrimination*, we use blue and teal to differentiate species along with a different silhouette. This is the same for the *mixture discrimination*, where the 3rd interval shows a mixture of two species highlighted by color (yellow and teal) and silhouette. Lastly, the *individiuals discrimination* shows the same silhouette for all three intervals, as all the sounds are sampled from the same species, and we use shading differentiate the different individuals selected from the same species.

-To this point, Figure 4A (bottom) is quite confusing and maybe a typo. Species A is listed for all birds, but the silhouettes appear to depict mixtures.

The *same species discrimination* experiment samples sounds from one species. The two standard intervals are sampled from one group and the target is sample from a different group. The two standard intervals are sampled from the same individuals but are unique waveforms, as the intervals have been created from different excerpts of the same recordings.

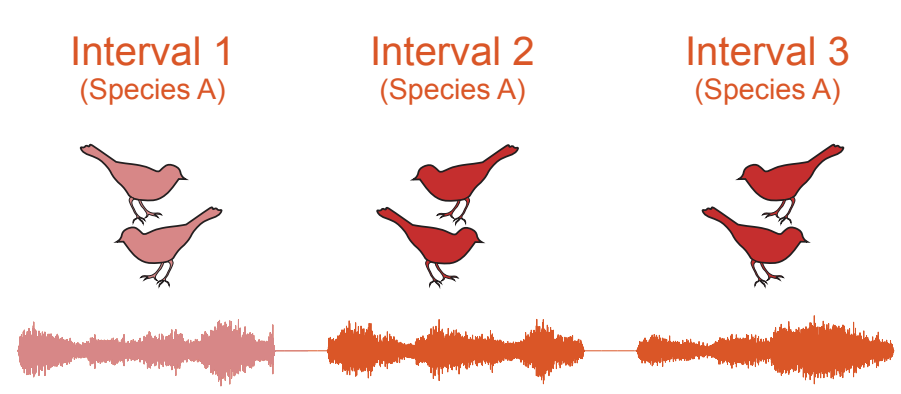

In the schematic above there are 3 intervals. All intervals are sampled from the same species. The first and second intervals is sample from one set of recordings (Group 1 – Species A) and the third interval is sampled from another set of recordings (Group 2 – Species A). The first and second intervals are different excerpts of the same set of recording.

The experiment was trying to test whether listeners could discriminate individuals from the same species.

The result of Experiment 4a showed that discrimination was possible when the chorus size was small (2 or 4 individuals) but approached chance when the chorus size was 8 or above.

Line 313-314: the order that you present these in are not in the same order as presented in Figure 4A (species, mixture, individual). I would consider changing the order to be consistent between the two.

Thank you. This has been corrected.

Figure 5A (legend): “an Euclidean” should be “a Euclidean”

Corrected. Thank you.

General appeal is wanting. E.g. if you used a bird texture model instead of human, how well do birds do? This is a nice human psychoacoustics study focusing on bird vocalization perception, but a little narrow in terms of broader appeal. I think better for a specialist acoustics journal

It is quite difficult to anticipate birds’ performance for the present discrimination tasks without making very strong (and potentially questionable) assumptions as to absolute thresholds, frequency selectivity, modulation-frequency selectivity and temporal resolution for the bird species under study (Vallée de Chevreuse). To the best of our knowledge, this information is available for Zebra Finch only as it was studied extensively over the last decades by Theunissen and coll., but Zebra Finch is not found in Vallée de Chevreuse (it is typical of Australian sub-arid regions). Pilot computer simulations reported in Lorenzi et al. (2023) illustrated the effects of reducing listening bandwidth, temporal and spectral resolution on the mid-level representation of natural scenes including bird songs based on Zebra Finch’s auditory characteristics. This pilot work indicates that, as suggested, it is technically possible to tune auditory-model parameters (such as the present auditory texture model) to bird specificities.

We agree that a comparative approach would be quite relevant and broaden the scope of the present study. However, human perspective on natural scenes – and especially bird choruses with or without acoustic background – has a broad appeal as such, first because of the ubiquity and over-representation of bird vocalizations in outdoor natural soundscapes (for a review, see Lorenzi et al., 2023), their specific contribution to restorative effects for humans (see Buxton et al., 2021) known to contribute significantly to well being and quality of life ; second, because the current listening situations (bird choruses) challenge principles of primitive auditory scene analysis (ASA) that have been mainly tested with artificial/lab stimuli (pure tones, noises) and speech or musical sounds. However, from an evolutionary perspective, primitive grouping mechanisms subserving primitive ASA must have predated urban/domestic situations, verbal communication and musical listening.

Figure 6B: which sound statistics were used to compute the distance? Texture model? Yes, the distance was computed between the statistics measured using the Auditory Texture Model. Text was added to the Figure 6 caption to make this explicit.

Figure 6B: It pains me to suggest this, as I think the panel is quite aesthetically pleasing, but I think the colormap should be the same across the 3 sound categories. It's very difficult for me to compare distances between different colored categories.

Yes, it is indeed more difficult to compare across the different mixtures and backgrounds. I have changed the figure colormap to be grayscale, which will make the comparison across all sounds easier. Thank you for this suggestion.

General (stylistic preference): The figure titles should be declarative, telling the reader the main result you show in the figure.

We have updated all the figure titles to be declarative, pointing to the main finding of the figures. The suggestion was very helpful, and will hopefully make the key results clearer to the reader. Thank you.

Figure 6D: I'm struggling to understand the difference between the single species experiment in 6D left and Figure 2D (same species). My previous comment about low same-species performance is put at ease in light of the 6D results, but I am left wondering specifically what is different between the two. Can the authors please comment on this?

The experiments are similar, 3-interval oddity tasks. However, there is a key difference in that the stimuli in experiment 6d (left) are created for an exemplar discrimination task, whereas the result so Figure 2D are from what we refer to as an "same species" discrimination task. The exemplar discrimination task the standard intervals are identical waveforms. The "same species" discrimination task, the standard intervals are sampled from the same individuals (same recording), but a different excerpt (i.e. different waveform). The "same species" task is more difficult as there is more variability across the three intervals. Hence, we see lower performance for the size 1 chorus condition for "same species" (Fig 2D) vs. the exemplar discrimination (Fig 6D).

We have added additional detail of the "same species" experiment in the methods section.

Line 526-528: Regarding background-to-foreground ratio, is there any empirical evidence for what the actual ratio is in nature?

The level selected for the background-to-foreground ratio was a simplification of the real-world situation, which can be dependent on the source level (how loud a bird species sings), the distance between the received and the source, the propagation of sound in the environment among other things. We have included two references to provide potential factors in the selection of the level. We, somewhat pragmatically, selected a SNR that would ensure both sources were audible to the listener.

SNRs probably vary greatly depending on species, time of day, distance, habitat, etc. In general, SNRs are quite low. In Michaud et al. (2025), the median SNR for Tengmalm's owls (individuals often far apart but in a very quiet nocturnal environment) is about 2 dB (see Figure 2).

Michaud, F., Sueur, J., Sèbe, F., Le Cesne, M., & Haupert, S. (2025). Acoustic detection of a nocturnal bird with deep learning: the challenge of low signal-to-noise ratio. *Ecological Indicators*, *181*, 114475.

Line 571: The sentence needs rephrasing, I'm not sure what it's trying to communicate.

This has been corrected.

Figure 7A: The observer model appears to achieve human-level performance in the 8+1* and 8+8* conditions, but achieves sub-human performance on the 8+1 and 8+8 conditions. My understanding is that the 8+1 and 8+8 conditions should be relatively easier to perform, since they contain species with acoustics most distinct from one another. Why then can the observer model get the asterisk conditions just fine (perceptually more difficult), but struggle on the non-asterisk conditions (perceptually more easy)?

Thank you for this thoughtful observation. Our interpretation of the model results, specifically how they compare to the human results, has to do with streaming. As stated, the observer model appears to capture the difference in performance for the 8+1* and 8+8* conditions. This could be because the similarity in statistics between the two species are sufficiently small that the listeners group the mixture into a single stream. In this case it may be 8+1* = 9 and 8+8* = 16. However, for the 8+1 and 8+8 mixture conditions, listeners may make use of some segregation of the two species, and can therefore discriminate on either auditory stream. The observer model does not segregate the input into streams, but averages the entire stimulus with a summary statistic. This is perhaps why the model underperforms relative to the human listeners for the 8+1 and 8+8 conditions. The oracle model is a window

into how an auditory texture model with streaming/segregation capabilities would perform. This model yields qualitatively similar performance to the 8+1 and 8+8 conditions but overperforms for the 8+1* and 8+8* conditions. We thought that the oracle model result provided further evidence of when listeners may segregate or group sound sources.

Figure 7: The oracle model is under-explained. A more detailed description would help the reader better understand differences in performance.

We have included additional detail of the oracle model in the caption of the Figure 7 as well as an expanded description in the Supplementary information: observer model section.

Line 626-627: “human listeners appear to lose access to the acoustic details of the chorus as its size increases” — do the authors provided quantitative evidence for this? One could use the observer model to substantiate the claim.

We have updated the sentence to read:

“Discrimination performance of exemplars decreased as a function of chorus size, suggesting listeners may lose access the to the temporal detail of the sound and transition to a summary statistic representation.”

Experiment 3a was similar in nature to that of McDermott et al. 2013 (experiment 4 and 5 in their paper), where listeners discrimination performance decreased as a function of number of talkers in a multi-talker babble stimuli. The McDermott experiment tested a range of stimulus durations and babble conditions. They found that performance was high at short durations (50ms) for all babble conditions, but at longer durations (2s) discrimination performance decreased as a function of the number of simultaneous talkers in the babble noise. They interpreted this decrease in performance as evidence of time-averaged summary statistics in auditory perception, but only for multi-talker babble (many talkers) at longer durations (“As sounds become more texture-like and of longer duration, listeners lose access to detailed temporal structure and become dependent on these summary statistics.” McDermott et al. 2013)

Experiment 3a is similar in that the duration of the stimuli and chorus size is varied. The results of Experiment 3a show discrimination performance is high at short durations (50ms), but at longer durations (2s) performance decreases as a function of chorus size.

Discussion

Line 702: “technic” should be “technique”, I think

Corrected. Thank you for finding this issue.

Discussion is otherwise comprehensive and well-written.

Thank you.

Reviewer #2:

Here the authors study human perception of natural bird vocalizations. They present results from multiple experiments examining discriminability of mixtures of multiple birds and bird species and the effects of sound duration, and they also assess discriminability in the presence of other natural background sounds. They use multiple acoustic features (e.g. spectrum, modulation, correlations) to evaluate differences across species and to predict/model human performance.

This is an interesting extension of ideas in computational audition about how sound summary statistics relate to perception of natural sound textures. The paper is well written, and the motivation, modeling, and figures are all clear as well. This is altogether an excellent paper, and I think it is likely to be of interest to many readers who study hearing. I have some suggestions but see these as mostly small improvements to an already strong paper.

Thank you for the comments.

Major Issues:

I wasn't entirely clear about the arguments relating to masking and foreground/background segregation in experiments 6a/b. Since this is a discrimination task as opposed to a recognition task, it's not obvious to me that the lack of a clear difference between foreground-only and foreground+background can be interpreted as an indication of segregation. This made it hard to parse some of the arguments in the results (line 528, 537, 548, 552). It would be helpful to have a clear statement of whether the background is frozen/unfrozen and how the results would be expected to look with/without segregation.

This is a very important point – thank you for raising it. Both the foreground and background sounds conform to the exemplar discrimination task. Therefore, the standard intervals are composed of the same sound exemplar (same waveform) and the target is a different exemplar from the same sound source. In this way, the standard intervals could be described as frozen, as the waveforms are identical. Listener could therefore perform the discrimination with either the foreground or background sound. However, the background sounds were chose to be texture-like, so that they were quite dense and temporally homogeneous.

My understanding (based on the geophony only/biophony only conditions) is that this is unfrozen noise, and the exemplars get different background waveforms. In this scenario, I could imagine that the presence of the background could improve discrimination as well as

reduce it. Since the SNR is fairly high for the larger choruses, I'm also not entirely sure how much masking is playing a role here (line 556).

The effects of masking for the large chorus condition is likely minor, as the SNR between the foreground (bird chorus) and the background (environmental sound) is approximately +15 dB.

By including a background only condition in the in Experiment 6, we were hoping to set a baseline performance for the task, where listeners should be at or around chance (33% for 3AFC) if the environmental sound is perceived as a texture. We did find that performance was above chance, between 45% and 50% correct, for the background only condition. However, the inclusion of the large chorus (32 birds) revealed that the performance was approximately equivalent to the background only condition.

As a further note on SNRs, they are probably vary greatly depending on species, time of day, distance, habitat, etc. In general, SNRs are quite low. In Michaud et al. (2025), the median SNR for Tengmalm's owls (individuals often far apart but in a very quiet nocturnal environment) is about 2 dB (see Figure 2).

Michaud, F., Sueur, J., Sèbe, F., Le Cesne, M., & Haupert, S. (2025). Acoustic detection of a nocturnal bird with deep learning: the challenge of low signal-to-noise ratio. *Ecological Indicators*, *181*, 114475.

It may be useful to make the claims in the text more neutral on these points and state that the human data is somewhat ambiguous about segregation/integration/masking. It may also be worthwhile to add some additional analysis....

1) One analysis could be to add model results for Exp 6 to Fig 7 (following Fig 6E/F). This may help adjudicate whether there is segregation, integration, or some mix consistent with the "suboptimal segregation" described for Exp 5.

Thank you for suggesting this approach to disentangling the segregation and integration question.

We have run the observer model on Experiment 6, which can now be found in Figure 7b. The results show that observer model performance qualitatively matches that of humans for the foreground only conditions (i.e. chorus size of 1, 8 or 32). However, the foreground+background conditions show a divergence in performance between the model and humans. The model appears to underperform relative to humans for conditions where the foreground sound is composed of smaller choruses (1+bg and 8+bg). However, the model and human performance is comparable for the large chorus condition (32+bg) and the background only condition.

One interpretation of the results is that the listeners segregate the foreground sound from the background when the chorus size is small. This interpretation is motivated by the fact performance is comparable between the 1 vs. 1+bg, 8 vs. 8+bg, and 32 vs. 32+bg conditions. Specifically, the performance is highest for the 1 and 1+bg conditions, lowest for the 32 and 32+bg conditions, and in-between the 8 and 8+bg conditions. The model result doesn't completely support this hypothesis, but this interpretation is consistent with the model results.

For the large chorus conditions, 32 and 32+bg, the listener may be segregating the sources, and since the individual sources are quite "dense" performance is likely to be low (see Experiment 3a). However, it could be that listeners are grouping the two sources together and estimating the statistics from the entire mixture. It's difficult to tell whether concurrent textures are segregated or integrated.

The modelling results offer an additional datapoint, which reveals an auditory texture model that operates on the entire scene does not account for human discrimination performance across a range of chorus sizes and foreground+background mixtures.

2) Another option might be to evaluate Exp 6 in a way that follows Fig 4D and evaluate whether there is any difference in discriminability depending on the similarity of the foreground species and specific backgrounds. Presenting these differences may help make the arguments about masking/segregation more clear. If this is something you choose to do, there is some recent work from Clonan et al. (J Neurosci, 2025) using summary statistics to predict speech intelligibility in natural backgrounds that may be relevant to discuss.

Because of the experiment paradigm, we didn't have sufficient data to perform this analysis. Unlike experiment 5, where listeners were presented with every condition (all mixture conditions), the experiment 6 listeners were presented with a subset of conditions.

Specifically, experiment 5 had mixture conditions with most similar and most different bird pairs across 4 conditions, along with 3 bird chorus size conditions, resulting in 350 trials.

Experiment 6 assumed that all the birds were a single source type, and across the 7 conditions there were only 63 trials, covering the 9 background types for the biophony and geophony experiments. In this way, we did not have each individual bird species paired with each background and we're unable to perform the suggested analysis.

Thank you for the thoughtful suggestion. We will likely keep this in mind for future experiments/studies.

Minor Issues:

Line 141 – It would be helpful to explain in more depth how the normalized Euclidean distance is calculated here for Fig 1E. In reading here and, also, the Fig 4/5 results, my biggest question was about how the distance is calculated when there are multiple features. In my opinion, it would be valuable to discuss explicitly in the results whether features are/ought to be z-scored or transformed or weighted (e.g. dB-transformed or A-weighting for spectrum or with learned coefficients).

We included an expanded description of Figure 1E in the results section. The description now highlights that the statistics classes were z-scored across sounds and normalized by the maximum distance between sounds in our database. We have also included a new Methods section titled "**Species distance metric (Normalized Euclidean distance of sound statistics):**"

Fig 3C/D – suggest adding a label/indication that the chorus size is 32.

We have added the chorus size to the legend of Figure 3C/D

Line 289 – "Experiment 3b species (circles) and abundance (squares) discrimination task." Should this say "exemplar (squares)"?

This has been updated. Thank you.

Line 356-359 – If I'm interpreting it correctly, it looks like the figure disagrees with the text here? In Fig 4E it looks like the strongest correlation is from Modulation Power (not spectrum) and the weakest is from Env CV (not Env Corr). It would also be useful to explicitly discuss the distance calculation/normalization here and the loudness and pitch features (since this is the first mention).

Thank you for finding this error. We have corrected the text to highlight Modulation Power as the strongest correlation and Env CV as the weakest. In addition, we have added a sentence describing how the normalized Euclidean distance was computed.

Fig 4D – it would be helpful to be able to connect the 1-10 numeric labels with species names, even if it's just in the caption

I have added the 10 species and corresponding numeric label to the Figure 4D. This was a very helpful suggestion. Thank you.

Line 962-964 – This wasn’t totally clear to me. Is time-averaged F0 calculated for individual birds and then mean and variance is calculated across birds? Or is this mean and variance over time for the sound mixture? And how is the distance then calculated - d’?

We have added clarifying text to the Methods section describing how the distance is calculated. The mean and variance are measured for each sound and are subsequently z-scored across sounds. The Euclidean distance is computed between the mean and variance of the fundamental frequency of the sound mixtures.

The motivation for using the mean and variance of the f0 is to make the “pitch” statistic time averaged. We thought this would make it easier to compare with all of the other statistics/auditory grouping feature that are also time-averaged.

Fig 6 – “E” and “F” panel labels might be switched

Thank you for finding this error. The figure labels have been updated.

Fig 6B – is the color scale for the bird sound similarity shifted? My expectation was that this would match Fig 1E, but somehow it is much less blue and much lower contrast. Fig 6E also seems to suggest to me that the blue should go all the way to ~0.

Another reviewer had asked to have the color scale normalized across the three sound types (though did mention how aesthetically pleasing the figure was). As a consequence, the shading is different than Fig 1E and Fig 6B are different.

However, the two plots are generated from the same measured statistics. One difference could be in the z-scoring. For Figure 1E, the sounds are Z-scored across the bird sound database. For Figure 6B the z-scoring includes the background sounds, both geophony and biophony. This should change the shading and similarity between bird species conditions.

Line 802 – it may be good to mention more specifically that/how source location might improve segregation/reduce masking. Binaural unmasking is a very active research area. We have added a sentence to elaborate on the scene analysis (sound segregation) based on the inclusion of source distance and position.

Title – I noticed that the title is similar to the title of the author's previous Grinfeder et al. paper. To avoid confusion, it may be useful to modify the title and make more of a distinction. I might also suggest including "bird biodiversity" for specificity.

Thank you for this suggestion. We agree completely. We have updated the title to "Human perception of avian biodiversity in naturalistic auditory scenes"

Reviewer #3

Summary

In this work, the authors investigate how human listeners perceive biodiversity in acoustic scenes composed of bird vocalizations, focusing on two components: species abundance and species richness. They construct a curated database of bird calls and synthesize bird choruses by summing different numbers of individuals and species. Across a series of oddity-discrimination experiments, they ask: (i) how well listeners can discriminate differences in chorus size within a species; (ii) how they discriminate between species and between mixtures of species; and (iii) how additional background sounds affect performance. They complement the behavioral data with several observer models, including an auditory texture model and simpler cue-based models. The findings are interpreted as evidence that large bird choruses are processed as auditory textures, represented via summary statistics, and that species mixtures are grouped or segregated depending on disparities in these statistics.

Overall, this is a carefully executed and thoughtfully framed study that is a natural next step to previous ecoacoustics studies from the authors' research group. It extends earlier work on perceived acoustic biodiversity and natural soundscape discrimination by:

• Switching from subjective numerosity judgments to objective oddity discrimination
• More cleanly manipulating abundance vs richness with synthetic choruses
• Leveraging auditory texture models and cue-based observers to interpret the behavioral data mechanistically

The broad conceptual story is plausible and appealing. The stimulus construction appears careful, the experimental paradigms are appropriate, and the modeling is a welcome addition. At the same time, some of the central interpretive claims are stated more strongly than the current analyses justify. In particular, the texture model is not clearly shown to be uniquely required relative to simpler, long-term cues, and the role of explicit streaming versus global scene statistics in mixtures remains somewhat qualitative. The ecoacoustic implications could also be more carefully delimited given the artificial nature of the stimuli. I believe the manuscript would benefit substantially from a more quantitative comparison of models, clearer framing of the evidence for texture and streaming, and a more nuanced discussion of ecological validity and the relationship to previous work from the same group.

Major comments

1. Specificity of texture perception. The behavioral and modeling results are nicely consistent with a summary-statistics / texture interpretation, especially for dense choruses. However, in a few places (e.g. the abstract and Discussion) the wording suggests that large choruses are definitively “perceived as textures” or “are perceived by their time-averaged statistics” .

Given that simpler long-term cues (e.g. spectrum, modulation power) appear to account for much of the observed pattern in performance, I would suggest slightly softening these

formulations (e.g. “consistent with a texture-like summary-statistics representation”) to better reflect the level of constraint provided by the current data. Cross-validated prediction accuracy or some formal measure (AIC/BIC, deviance) could help show whether the texture model adds explanatory value beyond simpler statistics. If it does not, the interpretive claims should be softened accordingly.

Thank you to the reviewer for identifying this issue. We have revised the manuscript to present the relationship between chorus size and texture perception as a plausible, though not definitive, explanation.

Specifically, we changed the abstract sentence highlighted to the following:

“We found that humans could discriminate mixtures but only up until around 8 individuals of the same species beyond which mixtures might depend on an time-averaged representation based on auditory texture summary statistics.”

In addition, we amended several sections of the discussion. The first paragraph of the discussion was changed to include following sections:

“When presented with mixtures of species-pairs, listeners performance was lower for mixture conditions when the sounds had similar statistics and performance was higher when mixture conditions had sound with similar statistics. The difference in performance could be evidence for grouping or segregation based on the statistical similarity of the mixture, and is generally consistent with general principles of auditory scene analysis already demonstrated with either artificial, music or speech stimuli [2].”

and

“The results showed listeners exemplar discrimination performance was significantly higher for conditions where the mixture statistics were different, even when the size of the chorus was the same. The difference in performance could be interpreted as listeners grouping mixtures of bird species when their statistics are similar, but segregating mixtures of bird species when their statistics were different.”

2. Quantitative delineation of streaming vs global statistics. The mixture experiments (especially Exp. 4–5) provide intriguing evidence that performance depends on which species are combined, consistent with the idea that listeners sometimes treat mixtures as a single fused source and sometimes access species-specific information. However, the current modeling mainly shows that a purely global texture representation is insufficient and that a fully segregated “oracle” also does not match human data, rather than precisely pinning down when and how streaming occurs. I would suggest slightly softening categorical

statements such as “listeners group mixtures when their statistics are similar but segregate species when their statistics are different,” and,

Thank you for the bringing up this important issue. Indeed, the conclusion of experiment 5 is overstated given the modeling results do not directly differentiate grouping vs. streaming. As suggested, we have softened the conclusion of experiment 5 by revising the sentence:

“Overall, the difference in performance between the bird mixture conditions suggests that listeners group sound mixtures when their statistics are similar but segregate species when their statistics are different.”

to

“Overall, performance differed across conditions, suggesting listeners discrimination performance was modulated by the chorus size. Specifically, the mixture conditions performance varied with the similarity of the sound statistics. The results are consistent with the notion that segregation is promoted by the diversity of the summary statistics and indicates that listeners may group bird choruses with similar statistics and segregate bird choruses with different statistics.”

...if possible, adding a brief quantitative analysis linking a continuous measure of acoustic similarity (e.g., distances in spectral/modulation/texture spaces) to performance across mixture conditions to more concretely support the role of similarity in promoting grouping vs segregation.

Thank you for the important suggestions.

We have created a new figure (Supporting Information S1: figure 5) that plots the continuous measure of acoustic similar against discrimination performance. We made the plots for the spectral, modulation power, and texture spaces. In addition, we included the envelope correlation, as it seemed to be one of the stats classes that seemed to account for the model performance (per the ablation analysis, supplemental figure 4).

The plot was divided into 2 rows: 21) 8+1 and 8+1*, and (2) 8+8 and 8+8, as well as 4 rows (i) texture, (ii) modulation power, (iii) envelope correlation, and (iv) spectrum.

All acoustic similarity spaces yielded a positive correlation with performance. The discrimination performance was lower when the sounds were more similar and performance was higher when the sounds were more different. Although there was a positive correlation, the correlation was not always significant.

3. Ecological validity and conclusion scope. The manuscript is framed in terms of natural parks, temperate forests, and biodiversity monitoring. However, the choruses used here are strongly artificial: up to 32 individuals of the same species, fully overlapping, no spatialization, no explicit modeling of species-specific call timing, and a relatively clean listening context compared to real forests.

This is not a criticism of the experimental design per se: controlled synthetic stimuli are appropriate for mechanistic psychophysics. It does, however, limit how directly the results can be generalized to real soundscapes and field-based ecoacoustic monitoring. The authors have already mentioned most, if not all, of the limitations I have described above individually in the Discussion section, but I suggest that they more explicitly acknowledge these limitations and define the ecoacoustic implications as mechanistic constraints on how humans might perceive abundance and richness, rather than as a direct account of human perception of actual natural choruses.It may also be useful to briefly relate these findings to the known limitations of common acoustic indices (which often correlate only moderately and inconsistently with biodiversity in real recordings), emphasizing that human perception relies on richer scene-analysis mechanisms than those indices capture.

Thank you for the significant observation. Indeed, the chorus size of 32 for the temperate forest region we selected as our test case is quite high.

We have added a paragraph in the discussion (Limitations section) to more clearly position the results relative to what would be deemed ecologically valid.

A recent computational study conducted Grinfeder et al. (2025b) tested an algorithm offering the possibility to simulate natural scenes from a protected European cold forest while controlling for the number of bird species and bird individuals, the temporal organization of their vocalizations, but also presence of geophysical background sounds (wind and rain with adjustable level), aircraft noise and effects of sound propagation. The generated soundscapes were then used to evaluate commonly-used ecoacoustic indices across a large range of controlled conditions. The simulations showed that all ecoacoustic indices were affected by background sounds, yielding underestimation or overestimation of bird-biodiversity levels. Importantly, a background of geophysical sounds was found to be the most challenging background condition for all ecoacoustic indices. This study demonstrates that presence of background sounds limits strongly the ability of the indices to assess biodiversity and its changes with high accuracy. These findings are consistent with conclusions of recent studies discussing the limitations of ecoacoustic indices (Alcocer et al., 2022; Llusia, 2024; Sethi et al., 2023). More importantly, this computational study showed that only ecoacoustic indices aiming to segregate bird vocalizations from background sounds consistently detected changes in biodiversity levels in background conditions, demonstrating the relevance of 'auditory scene analysis' prior to estimating the number of bird species, as achieved by the human auditory system.

References:

Alcocer, I., Herlander, L., Sugai, L.S.M., and Llusia, D. (2022). acoustic indices as proxies for biodiversity: a meta-analysis. Biol. Rev. 97, 2209–2236. https://doi.org/10.1111/brv.12890.

Grinfeder, E., Lorenzi, C., Teytaut, Y., Haupert, S., and Sueur, J. (2025b). Introducing evascape, a model-based soundscape assembler: impact of background sounds on biodiversity monitoring with ecoacoustic indices. Ecological Indicators, 178. doi : 10.1016/j.ecolind.2025.113882

Llusia, D., 2024. The limits of acoustic indices. Nat. Ecol. Evol. 8, 606–607. https://doi.org/10.1038/s41559-024-02348-1.

Sethi, S.S., Bick, A., Ewers, R.M., Klinck, H., Ramesh, V., Tuanmu, M.N., and Coomes, D.A., (2023). Limits to the accurate and generalizable use of soundscapes to monitor biodiversity. Nat. Ecol. Evol. 7, 1373–1378. https://doi.org/10.1038/s41559-023-02148-z.

4. Positioning relative to previous work. This study sits naturally alongside earlier work from the same research group on perceived acoustic biodiversity and natural soundscape perception. However, the manuscript currently does not fully spell out what is new relative to, for example, previous numerosity and soundscape discrimination studies like Grinfeder, et al. (2025).

I recommend adding a short paragraph in the Introduction and/or Discussion that explicitly summarizes how this work extends and complements those studies: use of objective oddity discrimination, cleaner manipulation of abundance vs richness in synthetic choruses, explicit texture/observer modeling, etc. This will help readers, especially those outside this subfield, understand the incremental conceptual advance.

We have added a paragraph to the Discussion that positions this work relative to the work of Grindfeder, et al. (2025) in the "Relationship to prior work" section.

"The psychophysical studies conducted here are based on objective three-interval forced-choice paradigms, namely the triangular or oddity method [60–62], which allow us to rigorously assess the sensitivity of our human observers by eliminating potential (decision-making) biases in these auditory discrimination tasks. These behavioral studies provide a characterization of the auditory abilities of human observers in these experiments on the perception of bird biodiversity in natural settings that differs from that reported in the pilot study conducted by using a subjective numerosity judgment paradigm based on a single observation interval and applied to bird choruses generated from data from another temperate forest [10,63]. The pilot study conducted by Grinfeder et al. (2025) reveals that human observers cannot estimate more than 3 or 4 birds within a natural soundscape simulating the contribution of ambient background noise and habitat transfer function as well as the vocal behavior of birds. This finding differs from the perceptual limit of 8 birds

observed in the first auditory discrimination task of bird chorus size using the forced-choice triangular method, suggesting that numerosity judgments – although more ecological – are limited by higher-level factors of a cognitive nature (e.g., attentional, decision-making). Thus, the perceived richness of bird choruses in natural environments is probably greater than previously [10]. Still, the previous results were generally consistent with the idea that streaming mechanisms contribute to the estimation of chorus size (perceived size was greater for choruses made out of birdsongs from different species compared to choruses made out of birdsongs from the same species), in line with the outcome of the present studies."

5. Statistical methodology. The behavioral results are mostly presented as percent correct per condition, analyzed with ANOVAs and multiple t-tests. This is understandable given the design, but it is not the most robust approach for complex, factorial psychophysical data. A mixed-effects logistic regression model (or other appropriate GLMMs) in this case would be more robust and make use of the within-participant performance variability. The model would for example have fixed effects for the main experimental factors (chorus size, species, mixture type, presence of background, etc.), and random effects for subjects. I am not asking the authors to redo all their analyses using mixed-effects models, but doing so would make the statistical basis of the findings stronger. I would therefore suggest that the authors apply such a model to at least the main experiment and report the results, for example in the supplementary materials.

Thank you again to the reviewer for suggesting the additional statistical analysis. The mixed-effects logistic regression analysis was applied to the main experiment (Experiment 1a and 1b). The results are summarized in the statistics and data analysis section of Experiment 1.

The model equation had the chorus size and species as fixed effects and the participants were random effects. The chorus size had 2 values, as the experiment was a comparison between intervals of sound that were composed of different sized choruses (in the figure they are written as N and M).

The model was implemented in MATLAB with the following function:

```
glme = fitglme(tbl,"s_resp ~ N + M + Species + (N | Participant) + (M | Participant) + (Species | Participant)",Distribution="Binomial");
```

where the table (tbl) contains the binary responses from the participants as well as the fixed and random effects.

The results show that there is a significant effect of chorus size and species. In addition, there is a significant effect of species. This was a somewhat unexpected result, but perhaps the species vary in the acoustic properties, and the way the individual species combine makes the discriminability different across chorus size.

Lastly, the participants (random effects) are non-significant except for 1 participant. Which suggests that the main effect driving the discriminability is chorus size and species.

6. Multiple comparisons. Considering the multiple comparisons performed in comparison groups, the authors should explicitly address how multiple comparisons were handled (e.g., correction methods or pre-specified contrasts). For the larger effect sizes this does not make a difference, but for example in experiment 5 (comparing the single-species chorus of 8 birds to the 8+1, 8+1*, 8+8, 8+8* mixtures) where some p-values are only just below 0.05, a correction for multiple comparisons can make these contrasts statistically non-significant.

Thank you for highlighting this important issue. We have re-run the analysis as a repeated measures ANOVA corrected (Bonferroni) for multiple comparisons. Indeed, with the original data (N=20), a key comparison changed from being significant to non-significant. Specifically, the 8+8 and 8+8* comparison was no longer significantly different.

To address this issue, we collected another 7 participants to raise the total number of participants for Experiment 5 to N=27.

The main manuscript text has been updated with the new significance values. In addition, the caption for Experiment 5 (Figure 6) has been updated to describe the statistical analysis conducted. Lastly, the methods section for Experiment 5 now includes additional details about the statistical analysis, as well as the significance values for the main experiment conditions.

This was a very important comment, and we would like to thank the reviewer again for raising this issue.

Minor comments

1. Participant description. The text would benefit from a brief, explicit description of participant demographics and experience beyond age and gender (e.g., hearing status, any exclusion criteria, and musical or ornithological background). Even if all participants were naive students, stating this clearly would help assess generalizability.
We have added several statements in the methods section related to subject recruitment and inclusion. All participants were self-reported normal hearing. There were no inclusion/exclusion criteria based on musical ability or ornithological background.

2. Code availability. At the time of review, the code does not appear to be accessible at the specified URL (likely because the repository is still private). It would be helpful for reviewers to have access to the code before final publication.
The GitHub repository is public and the experiment analysis and model code is being added.

3. Typos and clarity. There are a few typos in the manuscript, but one specifically makes the sentence unreadable. The sentence at line 77 is missing a word: I think it was meant to

be “Second, variation in the spectral and temporal characteristics of bird vocalizations…”. That paragraph is generally hard to read and could use some rephrasing for clarity.

Thank you for highlighting the clarity issues of this paragraph (and the introduction). We have corrected the sentence you identified as having an issue. We also restructured the paragraph with the goal of making it easier to parse.

Additional Editor Comments:

Dear Richard,

Your paper has now been reviewed by 3 experts in the field. You will see that they are for the most part quite positive and enthusiastic about this work but they have also raised both significant and minor issues that you can address. I look forward to reading your replies.

Frederic Theunissen

Journal Requirements:

1) Please ensure that the CRediT author contributions listed for every co-author are completed accurately and in full.

At this stage, the following Authors/Authors require contributions: Richard McWalter, and Christian Lorenzi. Please ensure that the full contributions of each author are acknowledged in the "Add/Edit/Remove Authors" section of our submission form. The list of CRediT author contributions may be found here: https://journals.plos.org/ploscompbiol/s/authorship#loc-author-contributions

This was done.

2) We ask that a manuscript source file is provided at Revision. Please upload your manuscript file as a .doc, .docx, .rtf or .tex. If you are providing a .tex file, please upload it under the item type LaTeX Source File and leave your .pdf version as the item type Manuscript.

This has been done.

3) Please upload all main figures as separate Figure files in .tif or .eps format. For more information about how to convert and format your figure files please see our guidelines: https://journals.plos.org/ploscompbiol/s/figures

This has been done.

4) We notice that your supplementary Figures are included in the manuscript file. Please remove them and upload them with the file type 'Supporting Information'. Please ensure that each Supporting Information file has a legend listed in the manuscript after the references list.

We have created separate 'Supporting Information' documents that is noted after the reference list.

5) Some material included in your submission may be copyrighted. According to PLOSs copyright policy, authors who use figures or other material (e.g., graphics, clipart, maps) from another author or copyright holder must demonstrate or obtain permission to publish this material under the Creative Commons Attribution 4.0 International (CC BY 4.0) License used by PLOS journals. Please closely review the details of PLOSs copyright requirements here: PLOS Licenses and Copyright. If you need to request permissions from a copyright holder, you may use PLOS's Copyright Content

Permission form.

Please respond directly to this email and provide any known details concerning your material's license terms and permissions required for reuse, even if you have not yet obtained copyright permissions or are unsure of your material's copyright compatibility. Once you have responded and addressed all other outstanding technical requirements, you may resubmit your manuscript within Editorial Manager.

Potential Copyright Issues:

- Figures 1, 2, 3, 4, and 6. Please confirm whether you drew the images / clip-art within the figure panels by hand. If you did not draw the images, please provide (a) a link to the source of the images or icons and their license / terms of use; or (b) written permission from the copyright holder to publish the images or icons under our CC BY 4.0 license. Alternatively, you may replace the images with open source alternatives. See these open source resources you may use to replace images / clip-art:

- https://commons.wikimedia.org
- https://openclipart.org/.

We have updated Figure 1, 2, 3, 4, and 6 so the images and clipart conform to CC BY 4.0. There is now a supporting information document that includes acknowledgments for the images used (all images from Wikimedia.org).

Figure 1a contains only original drawings or rainclouds, a bird, and insect, and an ear.

Figure 1b contains an original map.

Figure 1c contains images from Wikimedia.org with acknowledgements in S4.

Figure 1d contains an original model schematic.

Figure 2, 3, 4, and 6, all bird silhouettes are original drawings.

6) Please amend your detailed Financial Disclosure statement. This is published with the article. It must therefore be completed in full sentences and contain the exact wording you wish to be published.

- State the initials, alongside each funding source, of each author to receive each grant. For example: "This work was supported by the National Institutes of Health (####### to AM; ###### to CJ) and the National Science Foundation (###### to AM)."

- State what role the funders took in the study. If the funders had no role in your study, please state: "The funders had no role in study design, data collection and analysis, decision to publish, or preparation of the manuscript.".

If you did not receive any funding for this study, please simply state: The authors received no specific funding for this work.

This has been completed.
