# Supplementary material for: Human perception of avian biodiversity in naturalistic auditory scenes
Source: PLoS Comput Biol. 2026 Sep 21;22(9):e1014775. doi: 10.1371/journal.pcbi.1014775 (PMC13645227; doi:10.1371/journal.pcbi.1014775)
Supplement: Supplementary file 9 [file pcbi.1014775.s011.docx]

## Summary

The revised manuscript is substantially improved, and the authors have made a serious effort to address the reviewer comments. In particular, the framing of ecological validity is clearer, and the relationship to prior work is better developed. The additional model, ablation, and similarity analyses also help constrain the interpretation.

However, I have a few remaining issues that I think should be addressed before the paper is ready for acceptance. The newly added statistical reporting contains internal inconsistencies that need to be corrected, and the Experiment 5 effect-size reporting includes apparent numerical errors. I would also ask the authors to perform a careful copyedit before acceptance.

All line numbers refer to the tracked-changes version of the revised manuscript.

## Previous comments

My previously raised minor comments and Major comments 1–4 have been largely addressed to my satisfaction. Major comments 5–6 have unresolved issues.

**Major comment 5**

The new mixed-effects statistical analysis is a welcome addition, however there are several issues with the currently implemented methodology and reporting.

The authors have provided this formula in the response letter:

glme = fitglme(tbl,"s_resp ~ N + M + Species + (N | Participant) + (M | Participant) +

(Species | Participant)",Distribution="Binomial");

First, in Experiment 1, species richness is constant (every trial uses a single species), so the 'Species' term in the model must refer to species identity (which of 10 species was used). With 10 levels, a proper categorical coding would produce 9 contrast coefficients for the fixed effect and a 9- or 10-vector of random slopes per subject. The manuscript reports a single β for the fixed effect and a single β per subject for the random slope, which is only possible if Species was treated as a numeric variable (e.g., an alphabetical index 1-10). If that is what was done, please refit with Species as a properly categorical predictor and report either an overall LRT for the term or the full set of contrasts. If instead a categorical model was fit and a single contrast is being selectively reported, please state which species is the reference, which contrast is reported, and what the other contrast estimates were.

Second, in Experiment 1, N ≠ M on every trial, and the discrimination task is fundamentally about the dissimilarity between N and M (which chorus is the odd one out). The current model treats N and M as two independent linear predictors, which only recovers the discrimination signal if their coefficients happen to be equal and opposite. A more natural specification uses a single discriminability predictor — for instance, |log2(N) − log2(M)|, which takes the values 1–5 across the 30 chorus-size pairs and respects the doubling spacing of the stimulus design. I would suggest the following minimal alternative:

tbl.Species = categorical(tbl.Species);

tbl.AbsLogDiff = abs(log2(tbl.N) - log2(tbl.M));

glme = fitglme(tbl, 's_resp ~ AbsLogDiff + Species + (1 | Participant)', 'Distribution', 'Binomial', 'Link', 'logit');

Relative to the original formulation, this (i) replaces the two independent N and M predictors with a single discriminability variable that directly maps onto the experimental question, (ii) treats chorus size on the doubling scale that the stimuli were sampled on rather than the raw integer scale, and (iii) ensures that Species is a proper categorical predictor.

The treatment/reporting of Species as a predictor is essential to resolve. The N/M parameterization is a secondary concern, but if kept as-is, the authors should justify it and specify which coefficient is being interpreted as the chorus-size effect (N or M).

Third, besides the methodological concerns, the statistics reported in this section have internal inconsistencies. The most striking are in Experiment 1b:

- Negative standard error is impossible (Subject 1)
- Chorus-size β does not match its t
- Chorus-size CI is not centered on the reported β
- Chorus-size CI crosses zero, but the predictor is called "significant"

I would also like to acknowledge that the GLMM was added at my suggestion in the first round, and the analysis in its current form contributes relatively little beyond what the ANOVA-based results already show. If the authors prefer, an alternative to the corrections above is to simply remove the GLMM analysis from the manuscript, with a brief note in the Methods that the repeated-measures ANOVAs were the primary statistical analysis. I would consider either path — a corrected GLMM as specified above, or removal — fully responsive to my original comment.

**Major comment 6**

The statistics reported in manuscript lines 512-539 for Experiment 5 do not match those reported in Table 3 in the methods section. Please align the in-text values with Table 3, which appears to contain the correct numbers.

## New minor comments

Multiple copyediting mistakes were introduced during the revision. I suggest a thorough proof-reading, especially in the revised sections. Here’s a list of some of the examples I noticed in my reading, but there were more:

- Line 262: “that” is a typo / accidental insertion
- Line 306: “short to a long” was probably meant to be “short or long"
- Line 335: out-of-place “)” after “Experiment 3”
- Line 344: missing “of” after “sounds composed”
- Line 361: “was” should be removed in “with performance was relatively”
- Line 371: missing “of” between “type” and “bird species”
- Line 491: “a presented” -> “are presented”
- Line 558: “biopnony” -> “biophony”
- Line 563: “repeated measured ANOVA” -> “repeated measures ANOVA”
- Line 582: “ratio for was” -> “ratio was”
- Line 657: “Experiment N” is capitalized in most places in the manuscript, but sometimes like on this line it’s lowercase (“experiment 6”), would be best to use a consistent casing
- Line 1078: “(5) loudness, and (5) pitch” -> “(5) loudness, and (6) pitch”
- Line 1399: “the we found” -> “we found”
- Lines 1237, 1302, 1350, 1419: “3-inteval” -> “3-interval”
