# Supplementary material for: Human perception of avian biodiversity in naturalistic auditory scenes
Source: PLoS Comput Biol. 2026 Sep 21;22(9):e1014775. doi: 10.1371/journal.pcbi.1014775 (PMC13645227; doi:10.1371/journal.pcbi.1014775)
Supplement: Supplementary file 10 [file pcbi.1014775.s012.docx]

Dear Reviewers,

I would like to thank you very much for taking the time to evaluate and provide feedback on the manuscript. The manuscript has improved immeasurably from your comments, and the latest iteration is vastly improved from the first iteration.

The updated manuscript includes several corrections and improvements.

The major change includes the removal of the mixed-effects logistic regression analysis for Experiments 1a/b. There were several errors in the first analysis attempt, which were exposed by Reviewer 3’s carefully written and clear review. Although the analysis was removed from the manuscript, we followed through on the corrections and included them in the response to Reviewer 3. The main takeaway is that the mixed-effects logistic regression is congruent with the rmANOVA analysis provided in the main text. The mixed-effects analysis shows that both Experiments 1a/b replicated the main experiment findings: **large chorus ratios led to higher discrimination performance**. There were subtle differences between Experiment 1a and 1b in terms of species discrimination and individual differences, but the major model parameter driving performance was the difference in chorus size.

An additional change was a new paragraph in the Discussion section related to a comment from Reviewer 1 and the ablation analysis. The new paragraph offers the reader a better perspective on the ablation analysis and how the different statistics classes of the auditory texture model contribute to observer model performance.

Lastly, the statistics of Experiment 5 were brought in line with those found in Table 3, as highlighted by Reviewer 3. In addition, the rmANOVA on the single-species conditions of 1, 8 and 32 birds were updated to reflect the increase in participant size to N = 27 (and hence an F(2,52)).

We also identified a few areas where there were corrections needed. The first was in the reporting of participant numbers for Experiment 3a and Experiment 4a. These have been included in the updated methods section. Another change was the inclusion of the computation of the auditory grouping distance measures for Experiment 4b (Figure 5). These distances were computed on choruses of size 8, which corresponds to the size of the 2 choruses in the mixture condition (8+8) of Experiment 4b. A section in the Methods section has been added to make this distance computation clear. Lastly, statistics reporting for experiment 1 was reversed (Experiment 1a had ANOVA from Experiment 1b, and vice versa). This had been fixed. While uploading the model data to osf.io, we also noticed an issue with the Observer Model results for Experiment 1 (Figure 7 C&D) that was caused by reading in the wrong wave files for 1 of the 11 sound sets. The error has been resolved and the figure has been updated with the corrected model results.

Beyond these significant changes, there were several inconsistencies in naming conventions (e.g. within species 🡪 within-species, experiment 3 🡪 Experiment 3) that were corrected as well as several typos. These can all be found in the “tracked changes” version of the document.

Finally, PLOS requested changes to the images in Figure 1C due to licensing issues. The figure has been updated and now conforms with the journal licensing standards.

Thank you again to the Reviewers for the time and effort put towards the evaluation of the manuscript.

Kind regards,

Richard McWalter and Christian Lorenzi

## Reviewer 1

I thank the authors for their thorough and careful revision. They have addressed my concerns satisfactorily.

Thank you to Reviewer 1 for taking the time to evaluate the paper revisions; it is very much appreciated.

The addition of the ablation analysis (Supporting Information S1: Figure 4) is a welcome and informative contribution. The finding that Envelope Correlation has an outsized contribution to model performance meaningfully strengthens the interpretability of the texture model and broadens the general appeal of the manuscript. However, I note that the ablation results for Experiment 4b (Figure S1.4a) appear to show Modulation Power as the primary driver of MSE reduction, which would suggest the dominant cue differs across tasks. If so, this is an interesting and interpretable finding in its own right — Modulation Power driving species-level discrimination (Experiment 4b) and Envelope Correlation driving the finer-grained similarity judgments in Experiment 5 would be worth distinguishing explicitly in the discussion. The authors should clarify whether this discrepancy exists and, if so, discuss it rather than attributing the key role to Envelope Correlation across both experiments.

Thank you for the original suggestion of the ablation analysis. It was a useful and informative addition to the paper.

In order to reference and comment on the ablation results in the main manuscript, we have added a paragraph to the Discussion section.

“The auditory texture model was a good candidate model that qualitatively replicated the discrimination performance of human listeners across several experiments. However, the model included several classes of statistics, and it wasn’t clear how they contributed to the overall model performance. An ablation analysis (see Supporting Information S1: Figure 4) revealed that Modulation Power and Envelope Correlation played an outsized role in the observer model performance. However, a more nuanced view of the ablation results revealed that the contribution of the statistics classes varied across experiments: performance in Experiment 4b – species mixture discrimination – appeared to depend primarily on Modulation Power, whereas performance in Experiment 5 – species mixture exemplar discrimination – appeared to depend primarily on Envelope Correlation. The ablation analysis suggests Modulation Power may drive species-level discrimination, while Envelope Correlation may drive the finer-grained similarity judgments.”

The classifier and confusion matrix (Supporting Information S1: Figure 3) are also a clear supplement to the Euclidean distance metric alone, and the high cross-validated accuracy (≥90%) provides good validation of the bird sound database.

Thank you for suggesting the classifier and confusion matrix. This was a welcome addition and provided additional evidence for the Euclidean distance metric but also the composition of the bird sound database. Thank you again.

I accept the authors' explanation regarding the avian auditory model comparison. The lack of species-specific auditory parameters for the birds studied makes this infeasible at present, and the discussion of prior pilot work (Lorenzi et al., 2023) appropriately acknowledges the direction for future research.

Thank you. This suggestion will be a logical next step in this research program.

The writing revisions, figure improvements (particularly the unified colormap in Figure 6B and the declarative figure titles), and the expanded methods descriptions are all appreciated.

Your comments and suggestions were very welcome, and the new manuscript has dramatically improved because of your comments. Thank you.

## Reviewer 2

All of my concerns have been addressed. Thanks to the authors for their comprehensive response and edits

Thank you for the review. The auditory texture observer model results for Experiment 6 were a valuable addition. In addition, the new tone of the paper to bring the stated claims in line with the data was tremendously important. The paper improved considerably from your thoughtful comments. Thank you again.

## Reviewer 3

## Summary

The revised manuscript is substantially improved, and the authors have made a serious effort to address the reviewer comments. In particular, the framing of ecological validity is clearer, and the relationship to prior work is better developed. The additional model, ablation, and similarity analyses also help constrain the interpretation.

However, I have a few remaining issues that I think should be addressed before the paper is ready for acceptance. The newly added statistical reporting contains internal inconsistencies that need to be corrected, and the Experiment 5 effect-size reporting includes apparent numerical errors. I would also ask the authors to perform a careful copyedit before acceptance.

All line numbers refer to the tracked-changes version of the revised manuscript.

## Previous comments

My previously raised minor comments and Major comments 1–4 have been largely addressed to my satisfaction. Major comments 5–6 have unresolved issues.

**Major comment 5**

The new mixed-effects statistical analysis is a welcome addition, however there are several issues with the currently implemented methodology and reporting.

The authors have provided this formula in the response letter:

glme = fitglme(tbl,"s_resp ~ N + M + Species + (N | Participant) + (M | Participant) +

(Species | Participant)",Distribution="Binomial");

First, in Experiment 1, species richness is constant (every trial uses a single species), so the 'Species' term in the model must refer to species identity (which of 10 species was used). With 10 levels, a proper categorical coding would produce 9 contrast coefficients for the fixed effect and a 9- or 10-vector of random slopes per subject. The manuscript reports a single β for the fixed effect and a single β per subject for the random slope, which is only possible if Species was treated as a numeric variable (e.g., an alphabetical index 1-10). If that is what was done, please refit with Species as a properly categorical predictor and report either an overall LRT for the term or the full set of contrasts. If instead a categorical model was fit and a single contrast is being selectively reported, please state which species is the reference, which contrast is reported, and what the other contrast estimates were.

Second, in Experiment 1, N ≠ M on every trial, and the discrimination task is fundamentally about the dissimilarity between N and M (which chorus is the odd one out). The current model treats N and M as two independent linear predictors, which only recovers the discrimination signal if their coefficients happen to be equal and opposite. A more natural specification uses a single discriminability predictor — for instance, |log2(N) − log2(M)|, which takes the values 1–5 across the 30 chorus-size pairs and respects the doubling spacing of the stimulus design. I would suggest the following minimal alternative:

tbl.Species = categorical(tbl.Species);

tbl.AbsLogDiff = abs(log2(tbl.N) - log2(tbl.M));

glme = fitglme(tbl, 's_resp ~ AbsLogDiff + Species + (1 | Participant)', 'Distribution', 'Binomial', 'Link', 'logit');

Relative to the original formulation, this (i) replaces the two independent N and M predictors with a single discriminability variable that directly maps onto the experimental question, (ii) treats chorus size on the doubling scale that the stimuli were sampled on rather than the raw integer scale, and (iii) ensures that Species is a proper categorical predictor.

The treatment/reporting of Species as a predictor is essential to resolve. The N/M parameterization is a secondary concern, but if kept as-is, the authors should justify it and specify which coefficient is being interpreted as the chorus-size effect (N or M).

Third, besides the methodological concerns, the statistics reported in this section have internal inconsistencies. The most striking are in Experiment 1b:

- Negative standard error is impossible (Subject 1)
- Chorus-size β does not match its t
- Chorus-size CI is not centered on the reported β
- Chorus-size CI crosses zero, but the predictor is called "significant"

I would also like to acknowledge that the GLMM was added at my suggestion in the first round, and the analysis in its current form contributes relatively little beyond what the ANOVA-based results already show. If the authors prefer, an alternative to the corrections above is to simply remove the GLMM analysis from the manuscript, with a brief note in the Methods that the repeated-measures ANOVAs were the primary statistical analysis. I would consider either path — a corrected GLMM as specified above, or removal — fully responsive to my original comment.

Thank you for identifying the error in the analysis.

I would like to acknowledge, and very much appreciate, that your work and effort went above and beyond what was expected as a reviewer. Not only did you identify the errors in the analysis, but you also provided a clear analysis approach to resolve the errors. I especially appreciate you clarifying that species should be defined as categorical and that the chorus size comparison should be done as a log difference |log2(N)-log2(M)|. Thank you again.

We opted to remove the analysis from the methods section, as you offered as an option. However, we performed the analysis as you suggested to confirm that listeners were primarily sensitive to differences in chorus size and that the species and individual participants were less impactful on the results.

Discrimination performance was analyzed for both experiments (1a: Same individuals and 1b: Different individuals).

For Experiment 1a, the fixed effects and random effects were as follows:

```
Fixed effect coefficients: DFMethod = 'residual', Alpha = 0.05

    Name                               Estimate    SE          tStat       DF      pValue        Lower       Upper
    {'(Intercept)'            }        -0.34785     0.19013     -1.8295    2989      0.067426    -0.72065      0.024958
    {'Spec_Common Chaffinch'  }        -0.22982      0.1814     -1.2669    2989        0.2053    -0.58551       0.12587
    {'Spec_Common Chiffchaff' }          0.1032     0.18552     0.55627    2989       0.57807    -0.26056       0.46696
    {'Spec_Common Firecrest'  }        -0.30904     0.18073     -1.7099    2989      0.087387    -0.66341      0.045338
    {'Spec_Dunnock'           }        0.034033     0.18448     0.18447    2989       0.85365     -0.3277       0.39576
    {'Spec_Eurasian Blackbird'}        -0.26165     0.18112     -1.4446    2989       0.14866    -0.61679       0.09348
    {'Spec_Eurasian Blue Tit' }         0.13838     0.18608     0.74363    2989       0.45716    -0.22649       0.50324
    {'Spec_Eurasian Jay'      }        -0.34039      0.1805     -1.8859    2989       0.05941    -0.69431      0.013519
    {'Spec_Song Thrush'       }        -0.35601     0.18039     -1.9736    2989      0.048521    -0.70971    -0.0023135
    {'Spec_Willow Warbler'    }        -0.16548     0.18203    -0.90905    2989        0.3634     -0.5224       0.19145
    {'AbsLogDiff'             }         0.52236    0.036538      14.296    2989    6.7353e-45     0.45072         0.594


Random effect coefficients: DFMethod = 'residual', Alpha = 0.05

    Group            Level         Name                   Estimate     SEPred     tStat       DF      pValue        Lower        Upper
    {'SubID'}        {'1' }        {'(Intercept)'}          0.78945    0.17457      4.5223    2989    6.3539e-06      0.44717       1.1317
    {'SubID'}        {'2' }        {'(Intercept)'}          0.29266     0.1665      1.7577    2989      0.078908    -0.033816      0.61913
    {'SubID'}        {'3' }        {'(Intercept)'}         -0.36864    0.16142     -2.2838    2989      0.022453     -0.68514    -0.052144
    {'SubID'}        {'4' }        {'(Intercept)'}         -0.35508    0.16146     -2.1991    2989      0.027945     -0.67167    -0.038488
    {'SubID'}        {'5' }        {'(Intercept)'}          -0.2595    0.16186     -1.6033    2989       0.10898     -0.57686     0.057863
    {'SubID'}        {'6' }        {'(Intercept)'}        -0.078204    0.16293       -0.48    2989       0.63127     -0.39766      0.24126
    {'SubID'}        {'7' }        {'(Intercept)'}          0.30834     0.1667      1.8497    2989      0.064456    -0.018513       0.6352
    {'SubID'}        {'8' }        {'(Intercept)'}        -0.049758    0.16313    -0.30501    2989       0.76038     -0.36962      0.27011
    {'SubID'}        {'9' }        {'(Intercept)'}        0.0076678    0.16358    0.046874    2989       0.96262     -0.31308      0.32842
    {'SubID'}        {'10'}        {'(Intercept)'}         -0.28693    0.16173     -1.7741    2989      0.076149     -0.60405     0.030189
```

For the chorus size, the log2 absolute difference of the target to the standard was a significant predictor of discrimination performance (β = 0.522, SE = 0.037, t(2989) = 14.30, p < 0.001), revealing that the participants were more likely to correctly identify the target interval when the chorus size difference increased.

For the species, the differences were quite modest. Note that the Carrion Crow was used as the reference species. The Song Thrush was just significantly different from the reference (β = −0.356, SE = 0.180, t(2989) = −1.97, p = 0.049) and was harder to discriminate than the reference. The results suggest there was comparable discriminability across species when the samples were taken from the same individual.

For the participants (random effects), there were differences in baseline performance across participants. Three participants deviated from zero. Participant 1 performed significantly above the group average, and Participants 3 and 4 performed below average.

For Experiment 1b, the fixed effects and random effects were as follows:

```
Fixed effect coefficients: DFMethod = 'residual', Alpha = 0.05

    Name                               Estimate     SE          tStat        DF      pValue        Lower         Upper
    {'(Intercept)'            }         -0.93518     0.17401      -5.3745    2989     8.272e-08       -1.2764       -0.594
    {'Spec_Common Chaffinch'  }         0.015005     0.17323     0.086615    2989       0.93098      -0.32466      0.35467
    {'Spec_Common Chiffchaff' }          0.33662     0.17531       1.9201    2989      0.054935    -0.0071204      0.68037
    {'Spec_Common Firecrest'  }         -0.35767     0.17303      -2.0671    2989      0.038814      -0.69694    -0.018395
    {'Spec_Dunnock'           }          0.33662     0.17531       1.9201    2989      0.054935    -0.0071204      0.68037
    {'Spec_Eurasian Blackbird'}        -0.014987     0.17313    -0.086564    2989       0.93102      -0.35445      0.32448
    {'Spec_Eurasian Blue Tit' }         0.015005     0.17323     0.086615    2989       0.93098      -0.32466      0.35467
    {'Spec_Eurasian Jay'      }         -0.41748     0.17321      -2.4102    2989      0.016003      -0.75711    -0.077852
    {'Spec_Song Thrush'       }          0.10548     0.17363      0.60747    2989       0.54359      -0.23498      0.44593
    {'Spec_Willow Warbler'    }          0.35238     0.17546       2.0083    2989      0.044704     0.0083379      0.69641
    {'AbsLogDiff'             }          0.51503    0.033986       15.154    2989    4.9172e-50       0.44839      0.58167

Random effect coefficients: DFMethod = 'residual', Alpha = 0.05

    Group            Level         Name                   Estimate    SEPred     tStat      DF      pValue        Lower       Upper
    {'SubID'}        {'1' }        {'(Intercept)'}          0.7347    0.15208     4.8311    2989    1.4262e-06     0.43651       1.0329
    {'SubID'}        {'2' }        {'(Intercept)'}        -0.24128    0.14607    -1.6518    2989      0.098676    -0.52769     0.045127
    {'SubID'}        {'3' }        {'(Intercept)'}        -0.24128    0.14607    -1.6518    2989      0.098676    -0.52769     0.045127
    {'SubID'}        {'4' }        {'(Intercept)'}         0.13491    0.14695    0.91808    2989       0.35865    -0.15322      0.42304
    {'SubID'}        {'5' }        {'(Intercept)'}        -0.12542    0.14616    -0.8581    2989       0.39091    -0.41199      0.16116
    {'SubID'}        {'6' }        {'(Intercept)'}        0.095449    0.14677    0.65032    2989       0.51554    -0.19234      0.38324
    {'SubID'}        {'7' }        {'(Intercept)'}        0.082336    0.14672    0.56118    2989       0.57472    -0.20535      0.37002
    {'SubID'}        {'8' }        {'(Intercept)'}         -0.1512    0.14612    -1.0347    2989       0.30088    -0.43771      0.13532
    {'SubID'}        {'9' }        {'(Intercept)'}        -0.33132    0.14612    -2.2675    2989      0.023428    -0.61782    -0.044826
    {'SubID'}        {'10'}        {'(Intercept)'}        0.043107    0.14657     0.2941    2989        0.7687    -0.24429       0.3305
```

For the chorus size, again the log2 absolute difference of the target to the standard was a significant predictor of discrimination performance (β = 0.515, SE = 0.034, t(2989) = 15.15, p < 0.001), which is similar to Experiment 1a.

For the species, there were clearer differences in the species for the Exp 1b Difference Individuals experiment. The Eurasian Jay and Common Firecrest were significantly harder to discriminate than the reference species (i.e. Carrion Crow). For the Common Firecrest, this is somewhat expected as the bird song has a very narrow, high frequency song, that may not offer strong individual cues for discrimination. In contrast, the Eurasian Jay is statistically similar to the Carrion Crow (see Figure 1A), so it’s slightly surprising it was more difficult to discriminate. The Willow Warbler was slightly easier to discriminate than the reference species. The other species were not significantly different from the Carrion Crow reference species.

For the participants (random effects), there were again differences in baseline performance across participants. Two participants had significantly different performance from the group average. Participant 1 had better performance and Participant 9 was below the average.

It’s important to highlight that two species pairs yielded identical fixed-effects estimates: Common Chaffinch and Eurasian Blue Tit (Estimate = 0.015005) and Common Chiffchaff and Dunnock (Estimate = 0.33662). The two pairs happened to have the same number of correct trials (168/300 for the first pair and 189/300 for the second pair), which yields identical estimates, standard errors, and confidence intervals. The underlying data are indeed distinct and the trial-level data revealed the species performance curves were different (see Figure R1).

Figure R1 – Experiment 1b (different individuals) plot per species. Each species shows a unique curve across conditions.

Another noteworthy result was that Participants 2 and 3 had the same random intercept. This was a red flag as it might suggest a data duplication error. However, on closer inspection, the participants’ discrimination performance was indeed unique on a trial-by-trial basis. Although it may be unlikely, the finite number of trials (300) makes it possible that two participants might perform at the same level. Below is a plot of the individual participant curves for Experiment 1b, highlighting Participants 2 and 3. The results show the two participants have unique data (see Figure R2).

Figure R2 – Experiment 1b (different individuals) plot per participant. Each participant shows a unique curve across conditions. Note, Participant 2 and 3 are highlighted in bold as they had the same intercept.

As an additional check, we refit the model on a subset of the participants (the first 5 participants, N = 5). This perturbs each species’ number of total correct responses while leaving Participants 2 and 3’s data the same. The results show that the species now all have unique β estimates. However, the intercepts for Participants 2 and 3 are still the same, which is expected as their performance is unchanged. As we understand it, because of the balanced design of the mixed-effects model, equal accuracy produces equal estimates, both for the fixed-effects species coefficients and the predicted participant random-effects. We attribute these matches to coincidental ties in the number of correct trials rather than data duplication.

```
Fixed effect coefficients: DFMethod = 'residual', Alpha = 0.05
    Name                               Estimate       SE          tStat          DF      pValue        Lower       Upper
    {'(Intercept)'            }           -0.74385     0.27827        -2.6731    1489     0.0075983     -1.2897     -0.19799
    {'Spec_Common Chaffinch'  }           -0.37207     0.24963        -1.4905    1489       0.13631    -0.86175       0.1176
    {'Spec_Common Chiffchaff' }            0.26023     0.25541         1.0189    1489       0.30843    -0.24078      0.76124
    {'Spec_Common Firecrest'  }            -0.5552      0.2497        -2.2235    1489      0.026335      -1.045    -0.065395
    {'Spec_Dunnock'           }        -9.9464e-15     0.25186    -3.9492e-14    1489             1    -0.49404      0.49404
    {'Spec_Eurasian Blackbird'}          -0.063118     0.25125       -0.25121    1489       0.80168    -0.55596      0.42973
    {'Spec_Eurasian Blue Tit' }           -0.15677     0.25053       -0.62575    1489       0.53157    -0.64819      0.33466
    {'Spec_Eurasian Jay'      }           -0.77015     0.25073        -3.0717    1489     0.0021673      -1.262     -0.27833
    {'Spec_Song Thrush'       }           -0.18776     0.25033       -0.75003    1489       0.45335     -0.6788      0.30329
    {'Spec_Willow Warbler'    }            0.16094     0.25385        0.63398    1489       0.52619    -0.33701      0.65888
    {'AbsLogDiff'             }            0.55091    0.049514         11.126    1489    1.1136e-27     0.45378      0.64803

Random effect coefficients: DFMethod = 'residual', Alpha = 0.05
    Group            Level        Name                   Estimate    SEPred     tStat       DF      pValue        Lower       Upper
    {'SubID'}        {'1'}        {'(Intercept)'}         0.74231    0.21879      3.3928    1489    0.00071005     0.31314     1.1715
    {'SubID'}        {'2'}        {'(Intercept)'}        -0.31825    0.21469     -1.4824    1489       0.13845    -0.73937    0.10288
    {'SubID'}        {'3'}        {'(Intercept)'}        -0.31825    0.21469     -1.4824    1489       0.13845    -0.73937    0.10288
    {'SubID'}        {'4'}        {'(Intercept)'}        0.087537    0.21518      0.4068    1489       0.68421    -0.33456    0.50963
    {'SubID'}        {'5'}        {'(Intercept)'}        -0.19335    0.21471    -0.90054    1489       0.36798    -0.61451    0.22781
```

In summary, both experiments replicated the main experiment findings: **large chorus ratios led to higher discrimination performance**. The analysis showed similar slopes between Experiment 1a and 1b (Exp1a: β = 0.522; Exp1b: β = 0.515), which was also confirmed with an independent Bayesian analysis (Exp1a: β = 0.513; Exp1b: β = 0.519). The close agreement across experiments and analysis methods indicates that the discriminability of chorus size is stable and robust.

The main difference across the experiments is the overall response bias (performance). Exp 1a, where the samples were taken from the same individuals had higher performance (mean = 0.655) compared to Exp 1b, where samples were taken from different individuals (mean = 0.564). The decrease in performance for the different individuals experiment (Exp 1b) is consistent with the increased variability between the two standard intervals, making it more difficult for participants to form stable chorus size estimates.

There were differences in the discriminability of species relative to the reference (Carrion Crow) in Experiment 1a and Experiment 1b. Experiment 1a saw the Song Thrush as different from the reference. Experiment 1b saw the Common Firecrest and Eurasian Jay were more difficult to discriminate than the Carrion Crow, while the Willow Warbler was easier. The difference in species discriminability reveals a shift between the two experiments and may reflect some species generating more typical and stereotyped songs or choruses.

Lastly, individual differences were present and consistent across the two experiments, where the performance of Participant 1 was consistently above the group average.

Thank you again to Reviewer 3 for the time and care taken during the review. This was an important correction. Although the analysis was not included in the manuscript, the fact the results are consistent with the basic rmANOVA performed was reassuring.

**Major comment 6**

The statistics reported in manuscript lines 512-539 for Experiment 5 do not match those reported in Table 3 in the methods section. Please align the in-text values with Table 3, which appears to contain the correct numbers.

Thank you for finding this error. We have updated the Experiment 5 details in the manuscript to match Table 3.

## New minor comments

Multiple copyediting mistakes were introduced during the revision. I suggest a thorough proof-reading, especially in the revised sections. Here’s a list of some of the examples I noticed in my reading, but there were more:

We have attempted to be thorough in our latest review, resolving copyediting mistakes. Thank you very much for highlighting the issues below (which have been resolved).

- Line 262: “that” is a typo / accidental insertion
  - done
- Line 306: “short to a long” was probably meant to be “short or long"
  - done
- Line 335: out-of-place “)” after “Experiment 3”
  - done
- Line 344: missing “of” after “sounds composed”
  - done
- Line 361: “was” should be removed in “with performance was relatively”
  - done
- Line 371: missing “of” between “type” and “bird species”
  - done
- Line 491: “a presented” -> “are presented”
  - done
- Line 558: “biopnony” -> “biophony”
  - done
- Line 563: “repeated measured ANOVA” -> “repeated measures ANOVA”
  - done
- Line 582: “ratio for was” -> “ratio was”
  - done
- Line 657: “Experiment N” is capitalized in most places in the manuscript, but sometimes like on this line it’s lowercase (“experiment 6”), would be best to use a consistent casing
  - done
- Line 1078: “(5) loudness, and (5) pitch” -> “(5) loudness, and (6) pitch”
  - done
- Line 1399: “the we found” -> “we found”
  - done
- Lines 1237, 1302, 1350, 1419: “3-inteval” -> “3-interval”
  - done
