# Supplementary material for: Human perception of avian biodiversity in naturalistic auditory scenes
Source: PLoS Comput Biol. 2026 Sep 21;22(9):e1014775. doi: 10.1371/journal.pcbi.1014775 (PMC13645227; doi:10.1371/journal.pcbi.1014775)
Supplement: Peer review history

# Peer review: Decision Letter 0

**DOI:** 10.1371/journal.pcbi.1014775.r001

**Authors:** Frédéric E. Theunissen, Thomas Serre

20 Nov 2025

PCOMPBIOL-D-25-02004

Auditory Perception of Biodiversity in Humans

PLOS Computational Biology

Dear Dr. McWalter,

Thank you for submitting your manuscript to PLOS Computational Biology. After careful consideration, we feel that it has merit but does not fully meet PLOS Computational Biology's publication criteria as it currently stands. Therefore, we invite you to submit a revised version of the manuscript that addresses the points raised during the review process.

Please submit your revised manuscript by Jan 20 2026 11:59PM. If you will need more time than this to complete your revisions, please reply to this message or contact the journal office at ploscompbiol@plos.org. When you're ready to submit your revision, log on to https://www.editorialmanager.com/pcompbiol/ and select the 'Submissions Needing Revision' folder to locate your manuscript file.

Please include the following items when submitting your revised manuscript:

* A rebuttal letter that responds to each point raised by the editor and reviewer(s). You should upload this letter as a separate file labeled 'Response to Reviewers'. This file does not need to include responses to formatting updates and technical items listed in the 'Journal Requirements' section below.

* A marked-up copy of your manuscript that highlights changes made to the original version. You should upload this as a separate file labeled 'Revised Manuscript with Track Changes'.

* An unmarked version of your revised paper without tracked changes. You should upload this as a separate file labeled 'Manuscript'.

If you would like to make changes to your financial disclosure, competing interests statement, or data availability statement, please make these updates within the submission form at the time of resubmission. Guidelines for resubmitting your figure files are available below the reviewer comments at the end of this letter

We look forward to receiving your revised manuscript.

Kind regards,

Frédéric E. Theunissen

Academic Editor

PLOS Computational Biology

Thomas Serre

Section Editor

PLOS Computational Biology

**Additional Editor Comments:**

Dear Richard,

Your paper has now been reviewed by 3 experts in the field. You will see that they are for the most part quite positive and enthusiastic about this work but they have also raised both significant and minor issues that you can address. I look forward to reading your replies.

Frederic Theunissen

**Journal Requirements:**

1) Please ensure that the CRediT author contributions listed for every co-author are completed accurately and in full.

At this stage, the following Authors/Authors require contributions: Richard McWalter, and Christian Lorenzi. Please ensure that the full contributions of each author are acknowledged in the "Add/Edit/Remove Authors" section of our submission form.

The list of CRediT author contributions may be found here: https://journals.plos.org/ploscompbiol/s/authorship#loc-author-contributions

2) We ask that a manuscript source file is provided at Revision. Please upload your manuscript file as a .doc, .docx, .rtf or .tex. If you are providing a .tex file, please upload it under the item type u2018LaTeX Source Fileu2019 and leave your .pdf version as the item type u2018Manuscriptu2019.

3) Please upload all main figures as separate Figure files in .tif or .eps format. For more information about how to convert and format your figure files please see our guidelines:

https://journals.plos.org/ploscompbiol/s/figures

4) We notice that your supplementary Figures are included in the manuscript file. Please remove them and upload them with the file type 'Supporting Information'. Please ensure that each Supporting Information file has a legend listed in the manuscript after the references list.

5) Some material included in your submission may be copyrighted. According to PLOSu2019s copyright policy, authors who use figures or other material (e.g., graphics, clipart, maps) from another author or copyright holder must demonstrate or obtain permission to publish this material under the Creative Commons Attribution 4.0 International (CC BY 4.0) License used by PLOS journals. Please closely review the details of PLOSu2019s copyright requirements here: PLOS Licenses and Copyright. If you need to request permissions from a copyright holder, you may use PLOS's Copyright Content Permission form.

Please respond directly to this email and provide any known details concerning your material's license terms and permissions required for reuse, even if you have not yet obtained copyright permissions or are unsure of your material's copyright compatibility. Once you have responded and addressed all other outstanding technical requirements, you may resubmit your manuscript within Editorial Manager.

Potential Copyright Issues:

- Figures 1, 2, 3, 4, and 6. Please confirm whether you drew the images / clip-art within the figure panels by hand. If you did not draw the images, please provide (a) a link to the source of the images or icons and their license / terms of use; or (b) written permission from the copyright holder to publish the images or icons under our CC BY 4.0 license. Alternatively, you may replace the images with open source alternatives. See these open source resources you may use to replace images / clip-art:

- https://commons.wikimedia.org

- https://openclipart.org/.

6) Please amend your detailed Financial Disclosure statement. This is published with the article. It must therefore be completed in full sentences and contain the exact wording you wish to be published.

- State the initials, alongside each funding source, of each author to receive each grant. For example: "This work was supported by the National Institutes of Health (####### to AM; ###### to CJ) and the National Science Foundation (###### to AM)."

- State what role the funders took in the study. If the funders had no role in your study, please state: "The funders had no role in study design, data collection and analysis, decision to publish, or preparation of the manuscript.".

If you did not receive any funding for this study, please simply state: u201cThe authors received no specific funding for this work.u201d

**Reviewers' comments:**

Reviewer's Responses to Questions

**Comments to the Authors:**

**Please note here if the review is uploaded as an attachment.**

Reviewer #1: Pros:

The authors present a solid human psychoacoustics study focusing on bird vocalization perception with implications for auditory scene analysis. Using a battery of discrimination tasks, they demonstrate that humans can perceive differences in both the number of individuals (abundance) and the diversity of species (richness) within complex natural mixtures. This sensitivity declines once the chorus exceeds about 8 individuals, at which point the sounds are perceived as integrated auditory textures. Through behavioral experiments and computational modeling, the authors show that listeners use summary statistics rather than detailed temporal features to represent large mixtures. I appreciated the use of natural sounds in a psychophysics experiment, which isn't done as much as it should be.

Cons:

The manuscript text and clarity could be improved. I have noted areas for improvement + typos below. Although this work fills a necessary gap in the auditory psychoacoustics field, I worry the scope might be a little narrow for a general audience. For broader appeal, the authors could analyze the interpretability of the sound texture observer model, e.g. through ablations to identify which auditory features drive performance. They might also compare human texture models with bird-inspired or avian auditory models (if available) to test whether birds outperform humans, adding a valuable comparative dimension to the study.

Comments:

Introduction

Line 33: This sentence is hard to follow - structure is a bit off and is a run-on. Consider splitting and/or rephrasing.

Line 40: This sentence is hard to follow - structure is a bit off and is a run-on. Consider splitting and/or rephrasing.

Line 49-52: Which specific listening conditions are the authors referring to? The ecological-validity of the study could use much better framing here.

Line 54-56: This sentence is hard to follow - structure is a bit off and is a run-on. Consider splitting and/or rephrasing.

Line 59-61: I like this sentence a lot and it helps make the focus quite clear.

Line 62: Remove “The” to start the sentence.

Line 66-67: I think a word or two is missing after “sound recordings,”.

Line 108-110: It would be more appropriate to pose the questions as hypotheses with testable predictions, rather than listing them.

General: Introduction contains many sentences that are difficult to follow (marked above). Please consider splitting/rephrasing these sentences. Moreover, the framing around perception of bird species could be improved. The authors mention ecological validity of being able to detect different species, which could be expanded to address this concern. Why is it advantageous for humans to be able to segregate abundance and richness of birds specifically? I like the focus on natural sound processing generally, but the experiments end up focusing specifically on birds, whose findings are not necessarily generalizable to other natural sounds.

Results

Figure 1C

-Add time/frequency axes to one of the spectrograms please (if all the same time/frequency). If not, I recommend adding a time scale bar and frequency min/max for each example.

-What kind of pre-processing was done to the spectrograms? For example, the Dunnock spectrogram has increased noise around each syllable of the song, as if the rest of the recording has been denoised (same for Willow Warbler and Eurasian Blue Tit).

Line 139: Can the authors comment on how they (or Cornell) confirmed that vocalizations were emitted by individual birds? Was there visual or quantitative confirmation?

Figure 1E

-It seems like many species can be confused for each other. e.g. Common Chaffinch vs. Willow Warbler/Common Chiffchaff. Instead of Euclidean distance, can you perform a decoding analysis (perhaps SVM or Random Forest trained on features from the auditory model) and plot a confusion matrix? I think this would be more interpretable than the distance metric.

Supplementary Figure 1

-The within-bird recordings are truncated at different phases of their respective songs. I imagine this will affect how similar they are perceived?

Figure 2D

-It surprises me that within species discrimination (i.e. individual recognition) isn’t higher for n=m=1. Do the authors show any acoustic quantification of within-species differences that might help explain this? Also, are these statistics collapsed across all species? It would be interesting to see what the curve looks like separated by species.

Figure 3A (legend): “trail” should be “trial”.

Figure 3: what is the significance of .05s as the “short” trial?

FIgure 3D

-How do you go about selecting the 0.05s exemplar? Figure 1C show substantial within-song spectrotemporal variation. Therefore, some segments from the song (e.g. the ones with high variation) would be easily perceivable as species specific. Moreover, S1 shows that individual exemplars vary in time, where some examples contain an entire song, while others contain truncated versions of the song. Further elaboration is desirable for: 1.) how exemplars were chosen and 2.) whether the aforementioned potential confounders affect interpretation of results

Figure 4A (and throughout the manuscript)

-It’s difficult to appreciate the difference in silhouette between species in 4A. Perhaps color code (or have use a unique opacity for) each species?

-To this point, Figure 4A (bottom) is quite confusing and maybe a typo. Species A is listed for all birds, but the silhouettes appear to depict mixtures.

Line 313-314: the order that you present these in are not in the same order as presented in Figure 4A (species, mixture, individual). I would consider changing the order to be consistent between the two.

Figure 5A (legend): “an Euclidean” should be “a Euclidean”

General appeal is wanting. E.g. if you used a bird texture model instead of human, how well do birds do? This is a nice human psychoacoustics study focusing on bird vocalization perception, but a little narrow in terms of broader appeal. I think better for a specialist acoustics journal

Figure 6B: which sound statistics were used to compute the distance? Texture model?

Figure 6B: It pains me to suggest this, as I think the panel is quite aesthetically pleasing, but I think the colormap should be the same across the 3 sound categories. It’s very difficult for me to compare distances between different colored categories.

General (stylistic preference): The figure titles should be declarative, telling the reader the main result you show in the figure.

Figure 6D: I’m struggling to understand the difference between the single species experiment in 6D left and Figure 2D (same species). My previous comment about low same-species performance is put at ease in light of the 6D results, but I am left wondering specifically what is different between the two. Can the authors please comment on this?

Line 526-528: Regarding background-to-foreground ratio, is there any empirical evidence for what the actual ratio is in nature?

Line 571: The sentence needs rephrasing, I’m not sure what it’s trying to communicate.

Figure 7A: The observer model appears to achieve human-level performance in the 8+1* and 8+8* conditions, but achieves sub-human performance on the 8+1 and 8+8 conditions. My understanding is that the 8+1 and 8+8 conditions should be relatively easier to perform, since they contain species with acoustics most distinct from one another. Why then can the observer model get the asterisk conditions just fine (perceptually more difficult), but struggle on the non-asterisk conditions (perceptually more easy)?

Figure 7: The oracle model is under-explained. A more detailed description would help the reader better understand differences in performance.

Line 626-627: “human listeners appear to lose access to the acoustic details of the chorus as its size increases” — do the authors provided quantitative evidence for this? One could use the observer model to substantiate the claim.

Discussion

Line 702: “technic” should be “technique”, I think

Discussion is otherwise comprehensive and well-written.

Reviewer #2: Here the authors study human perception of natural bird vocalizations. They present results from multiple experiments examining discriminability of mixtures of multiple birds and bird species and the effects of sound duration, and they also assess discriminability in the presence of other natural background sounds. They use multiple acoustic features (e.g. spectrum, modulation, correlations) to evaluate differences across species and to predict/model human performance.

This is an interesting extension of ideas in computational audition about how sound summary statistics relate to perception of natural sound textures. The paper is well written, and the motivation, modeling, and figures are all clear as well. This is altogether an excellent paper, and I think it is likely to be of interest to many readers who study hearing. I have some suggestions but see these as mostly small improvements to an already strong paper.

Major Issues:

I wasn’t entirely clear about the arguments relating to masking and foreground/background segregation in experiments 6a/b. Since this is a discrimination task as opposed to a recognition task, it’s not obvious to me that the lack of a clear difference between foreground-only and foreground+background can be interpreted as an indication of segregation. This made it hard to parse some of the arguments in the results (line 528, 537, 548, 552). It would be helpful to have a clear statement of whether the background is frozen/unfrozen and how the results would be expected to look with/without segregation. My understanding (based on the geophony only/biophony only conditions) is that this is unfrozen noise, and the exemplars get different background waveforms. In this scenario, I could imagine that the presence of the background could improve discrimination as well as reduce it. Since the SNR is fairly high for the larger choruses, I’m also not entirely sure how much masking is playing a role here (line 556).

It may be useful to make the claims in the text more neutral on these points and state that the human data is somewhat ambiguous about segregation/integration/masking. It may also be worthwhile to add some additional analysis….

1) One analysis could be to add model results for Exp 6 to Fig 7 (following Fig 6E/F). This may help adjudicate whether there is segregation, integration, or some mix consistent with the “suboptimal segregation” described for Exp 5.

2) Another option might be to evaluate Exp 6 in a way that follows Fig 4D and evaluate whether there is any difference in discriminability depending on the similarity of the foreground species and specific backgrounds. Presenting these differences may help make the arguments about masking/segregation more clear. If this is something you choose to do, there is some recent work from Clonan et al. (J Neurosci, 2025) using summary statistics to predict speech intelligibility in natural backgrounds that may be relevant to discuss.

Minor Issues:

Line 141 – It would be helpful to explain in more depth how the normalized Euclidean distance is calculated here for Fig 1E. In reading here and, also, the Fig 4/5 results, my biggest question was about how the distance is calculated when there are multiple features. In my opinion, it would be valuable to discuss explicitly in the results whether features are/ought to be z-scored or transformed or weighted (e.g. dB-transformed or A-weighting for spectrum or with learned coefficients).

Fig 3C/D – suggest adding a label/indication that the chorus size is 32.

Line 289 – “Experiment 3b species (circles) and abundance (squares) discrimination task.” Should this say “exemplar (squares)”?

Line 356-359 – If I’m interpreting it correctly, it looks like the figure disagrees with the text here? In Fig 4E it looks like the strongest correlation is from Modulation Power (not spectrum) and the weakest is from Env CV (not Env Corr). It would also be useful to explicitly discuss the distance calculation/normalization here and the loudness and pitch features (since this is the first mention).

Fig 4D – it would be helpful to be able to connect the 1-10 numeric labels with species names, even if it’s just in the caption

Line 962-964 – This wasn’t totally clear to me. Is time-averaged F0 calculated for individual birds and then mean and variance is calculated across birds? Or is this mean and variance over time for the sound mixture? And how is the distance then calculated - d’?

Fig 6 – “E” and “F” panel labels might be switched

Fig 6B – is the color scale for the bird sound similarity shifted? My expectation was that this would match Fig 1E, but somehow it is much less blue and much lower contrast. Fig 6E also seems to suggest to me that the blue should go all the way to ~0.

Line 802 – it may be good to mention more specifically that/how source location might improve segregation/reduce masking. Binaural unmasking is a very active research area.

Title – I noticed that the title is similar to the title of the author’s previous Grinfeder et al. paper. To avoid confusion, it may be useful to modify the title and make more of a distinction. I might also suggest including “bird biodiversity” for specificity.

Reviewer #3: Review attached as PDF

**********

**Have the authors made all data and (if applicable) computational code underlying the findings in their manuscript fully available?**

The PLOS Data policy requires authors to make all data and code underlying the findings described in their manuscript fully available without restriction, with rare exception (please refer to the Data Availability Statement in the manuscript PDF file). The data and code should be provided as part of the manuscript or its supporting information, or deposited to a public repository. For example, in addition to summary statistics, the data points behind means, medians and variance measures should be available. If there are restrictions on publicly sharing data or code —e.g. participant privacy or use of data from a third party—those must be specified.

Reviewer #1: **No:** I got a 404 error when trying to access the GitHub repo

Reviewer #2: **No:** github repository is not yet public

Reviewer #3: **No:** While the authors intended to make the code available, it is currently inaccessible.

**********

PLOS authors have the option to publish the peer review history of their article (what does this mean?). If published, this will include your full peer review and any attached files.

If you choose “no”, your identity will remain anonymous but your review may still be made public.

**Do you want your identity to be public for this peer review?**  For information about this choice, including consent withdrawal, please see our Privacy Policy.

Reviewer #1: No

Reviewer #2: No

Reviewer #3: No

[NOTE: If reviewer comments were submitted as an attachment file, they will be attached to this email and accessible via the submission site. Please log into your account, locate the manuscript record, and check for the action link "View Attachments". If this link does not appear, there are no attachment files.]

**Figure resubmission:**

While revising your submission, we strongly recommend that you use PLOS’s NAAS tool (https://ngplosjournals.pagemajik.ai/artanalysis) to test your figure files. NAAS can convert your figure files to the TIFF file type and meet basic requirements (such as print size, resolution), or provide you with a report on issues that do not meet our requirements and that NAAS cannot fix.

After uploading your figures to PLOS’s NAAS tool - https://ngplosjournals.pagemajik.ai/artanalysis, NAAS will process the files provided and display the results in the "Uploaded Files" section of the page as the processing is complete. If the uploaded figures meet our requirements (or NAAS is able to fix the files to meet our requirements), the figure will be marked as "fixed" above. If NAAS is unable to fix the files, a red "failed" label will appear above. When NAAS has confirmed that the figure files meet our requirements, please download the file via the download option, and include these NAAS processed figure files when submitting your revised manuscript.

**Reproducibility:**

To enhance the reproducibility of your results, we recommend that authors of applicable studies deposit laboratory protocols in protocols.io, where a protocol can be assigned its own identifier (DOI) such that it can be cited independently in the future. Additionally, PLOS ONE offers an option to publish peer-reviewed clinical study protocols. Read more information on sharing protocols at https://plos.org/protocols?utm_medium=editorial-email&utm_source=authorletters&utm_campaign=protocols

Attachment Submitted filename: PCOMPBIOL-D-25-02004_Review.pdf

# Author comment: Author response to Decision Letter 1

**DOI:** 10.1371/journal.pcbi.1014775.r002

6 Apr 2026

Attachment Submitted filename: McWalter_Lorenzi_PLOSCompBio_Response_to_Reviewers.pdf

# Peer review: Decision Letter 1

**DOI:** 10.1371/journal.pcbi.1014775.r003

**Authors:** Frédéric E. Theunissen, Thomas Serre

12 May 2026

PCOMPBIOL-D-25-02004R1

Human perception of avian biodiversity in naturalistic auditory scenes

PLOS Computational Biology

Dear Dr. McWalter,

Thank you for submitting your manuscript to PLOS Computational Biology. After careful consideration, we feel that it has merit but does not fully meet PLOS Computational Biology's publication criteria as it currently stands. Therefore, we invite you to submit a revised version of the manuscript that addresses the points raised during the review process.

Please submit your revised manuscript by Jul 12 2026 11:59PM. If you will need more time than this to complete your revisions, please reply to this message or contact the journal office at ploscompbiol@plos.org. When you're ready to submit your revision, log on to https://www.editorialmanager.com/pcompbiol/ and select the 'Submissions Needing Revision' folder to locate your manuscript file.

Please include the following items when submitting your revised manuscript:

* A letter that responds to each point raised by the editor and reviewer(s). You should upload this letter as a separate file labeled 'Response to Reviewers'. This file does not need to include responses to formatting updates and technical items listed in the 'Journal Requirements' section below.

* A marked-up copy of your manuscript that highlights changes made to the original version. You should upload this as a separate file labeled 'Revised Manuscript with Track Changes'.

* An unmarked version of your revised paper without tracked changes. You should upload this as a separate file labeled 'Manuscript'.

If you would like to make changes to your financial disclosure, competing interests statement, or data availability statement, please make these updates within the submission form at the time of resubmission. Guidelines for resubmitting your figure files are available below the reviewer comments at the end of this letter.

As the corresponding author, your ORCID iD is verified in the submission system and will appear in the published article. PLOS supports the use of ORCID, and we encourage all coauthors to register for an ORCID iD and use it as well. Please encourage your coauthors to verify their ORCID iD within the submission system before final acceptance, as unverified ORCID iDs will not appear in the published article. Only the individual author can complete the verification step; PLOS staff cannot verify ORCID iDs on behalf of authors.

We look forward to receiving your revised manuscript.

Kind regards,

Frédéric E. Theunissen

Academic Editor

PLOS Computational Biology

Thomas Serre

Section Editor

PLOS Computational Biology

**Additional Editor Comments:**

Dear Richard,

As you will read the reviewers are satisfied with this revised version but reviewer 3 has questions on your statistical models that need to be addressed. I did not take the time (yet) to dive deep into these details but I will when I get your reply. Looking forward to seeing that final version!

Frederic.

**Reviewers' comments:**

Reviewer's Responses to Questions

**Comments to the Authors:**

**Please note here if the review is uploaded as an attachment.**

Reviewer #1: I thank the authors for their thorough and careful revision. They have addressed my concerns satisfactorily.

The addition of the ablation analysis (Supporting Information S1: Figure 4) is a welcome and informative contribution. The finding that Envelope Correlation has an outsized contribution to model performance meaningfully strengthens the interpretability of the texture model and broadens the general appeal of the manuscript. However, I note that the ablation results for Experiment 4b (Figure S1.4a) appear to show Modulation Power as the primary driver of MSE reduction, which would suggest the dominant cue differs across tasks. If so, this is an interesting and interpretable finding in its own right — Modulation Power driving species-level discrimination (Experiment 4b) and Envelope Correlation driving the finer-grained similarity judgments in Experiment 5 would be worth distinguishing explicitly in the discussion. The authors should clarify whether this discrepancy exists and, if so, discuss it rather than attributing the key role to Envelope Correlation across both experiments.

The classifier and confusion matrix (Supporting Information S1: Figure 3) are also a clear supplement to the Euclidean distance metric alone, and the high cross-validated accuracy (≥90%) provides good validation of the bird sound database.

I accept the authors' explanation regarding the avian auditory model comparison. The lack of species-specific auditory parameters for the birds studied makes this infeasible at present, and the discussion of prior pilot work (Lorenzi et al., 2023) appropriately acknowledges the direction for future research.

The writing revisions, figure improvements (particularly the unified colormap in Figure 6B and the declarative figure titles), and the expanded methods descriptions are all appreciated.

Reviewer #2: All of my concerns have been addressed. Thanks to the authors for their comprehensive response and edits

Reviewer #3: Review uploaded as an attachment.

**********

**Have the authors made all data and (if applicable) computational code underlying the findings in their manuscript fully available?**

The PLOS Data policy requires authors to make all data and code underlying the findings described in their manuscript fully available without restriction, with rare exception (please refer to the Data Availability Statement in the manuscript PDF file). The data and code should be provided as part of the manuscript or its supporting information, or deposited to a public repository. For example, in addition to summary statistics, the data points behind means, medians and variance measures should be available. If there are restrictions on publicly sharing data or code —e.g. participant privacy or use of data from a third party—those must be specified.

Reviewer #1: None

Reviewer #2: None

Reviewer #3: Yes

**********

PLOS authors have the option to publish the peer review history of their article (what does this mean?). If published, this will include your full peer review and any attached files.

If you choose “no”, your identity will remain anonymous but your review may still be made public.

**Do you want your identity to be public for this peer review?**  For information about this choice, including consent withdrawal, please see our Privacy Policy.

Reviewer #1: No

Reviewer #2: No

Reviewer #3: **Yes:** Menoua Keshishian

[NOTE: If reviewer comments were submitted as an attachment file, they will be attached to this email and accessible via the submission site. Please log into your account, locate the manuscript record, and check for the action link "View Attachments". If this link does not appear, there are no attachment files.]

**Figure resubmission:**

While revising your submission, we strongly recommend that you use PLOS’s NAAS tool (https://ngplosjournals.pagemajik.ai/artanalysis) to test your figure files. NAAS can convert your figure files to the TIFF file type and meet basic requirements (such as print size, resolution), or provide you with a report on issues that do not meet our requirements and that NAAS cannot fix.

After uploading your figures to PLOS’s NAAS tool - https://ngplosjournals.pagemajik.ai/artanalysis, NAAS will process the files provided and display the results in the "Uploaded Files" section of the page as the processing is complete. If the uploaded figures meet our requirements (or NAAS is able to fix the files to meet our requirements), the figure will be marked as "fixed" above. If NAAS is unable to fix the files, a red "failed" label will appear above. When NAAS has confirmed that the figure files meet our requirements, please download the file via the download option, and include these NAAS processed figure files when submitting your revised manuscript.

**Reproducibility:**

To enhance the reproducibility of your results, we recommend that authors of applicable studies deposit laboratory protocols in protocols.io, where a protocol can be assigned its own identifier (DOI) such that it can be cited independently in the future. Additionally, PLOS ONE offers an option to publish peer-reviewed clinical study protocols. Read more information on sharing protocols at https://plos.org/protocols?utm_medium=editorial-email&utm_source=authorletters&utm_campaign=protocols

Attachment Submitted filename: PCOMPBIOL-D-25-02004_Review2.docx

# Author comment: Author response to Decision Letter 2

**DOI:** 10.1371/journal.pcbi.1014775.r004

13 Aug 2026

Attachment Submitted filename: Response to Reviewers.docx

# Editor report: Decision Letter 2

**DOI:** 10.1371/journal.pcbi.1014775.r005

**Authors:** Frédéric E. Theunissen, Thomas Serre

31 Aug 2026

Dear McWalter,

We are pleased to inform you that your manuscript 'Human perception of avian biodiversity in naturalistic auditory scenes' has been provisionally accepted for publication in PLOS Computational Biology.

Before your manuscript can be formally accepted you will need to complete some formatting changes, which you will receive in a follow up email. A member of our team will be in touch with a set of requests.

Please note that your manuscript will not be scheduled for publication until you have made the required changes, so a swift response is appreciated.

IMPORTANT: The editorial review process is now complete. PLOS will only permit corrections to spelling, formatting or significant scientific errors from this point onwards. Requests for major changes, or any which affect the scientific understanding of your work, will cause delays to the publication date of your manuscript.

Should you, your institution's press office or the journal office choose to press release your paper, you will automatically be opted out of early publication. We ask that you notify us now if you or your institution is planning to press release the article. All press must be co-ordinated with PLOS.

Thank you again for supporting Open Access publishing; we are looking forward to publishing your work in PLOS Computational Biology.

Best regards,

Frédéric E. Theunissen

Academic Editor

PLOS Computational Biology

Thomas Serre

Section Editor

PLOS Computational Biology

***********************************************************

Dear Richard and Christian,

Thank you for your revised manuscript. We appreciate your thorough responses to all of the reviewer's comments. Your paper clearly describes, the interesting interplay of species abundance and species richness in humans assessment of bird choruses and by extension of biodiversity in natural sounds. Your analyses of the acoustic information bearing features that could explain these psychophysical results is also thorough: your modeling of auditory texture processing and your analyses on how and why it matches human behavioral results is very satisfying. As you mention, this work might also suggest novel signal processing approaches for assessing biodiversity. In this respect, is the performance of machine learning approaches to the estimation of the size of a chorus known? I don't think this is mentioned in your paper and I did not look at your references (e.g. 15, 21, 22). It might be something to also propose in future work in ecoacoustics?

Congratulations on a very nice contribution.

Frederic Theunissen.

# Editor report: Acceptance letter

**DOI:** 10.1371/journal.pcbi.1014775.r006

**Authors:** Frédéric E. Theunissen, Thomas Serre

PCOMPBIOL-D-25-02004R2

Human perception of avian biodiversity in naturalistic auditory scenes

Dear Dr McWalter,

I am pleased to inform you that your manuscript has been formally accepted for publication in PLOS Computational Biology. Your manuscript is now with our production department and you will be notified of the publication date in due course.

The corresponding author will soon be receiving a typeset proof for review, to ensure errors have not been introduced during production. Please review the PDF proof of your manuscript carefully, as this is the last chance to correct any errors. Please note that major changes, or those which affect the scientific understanding of the work, will likely cause delays to the publication date of your manuscript.

Soon after your final files are uploaded, unless you have opted out, the early version of your manuscript will be published online. The date of the early version will be your article's publication date. The final article will be published to the same URL, and all versions of the paper will be accessible to readers.

For Research, Software, and Methods articles, you will receive an invoice from PLOS for your publication fee after your manuscript has reached the completed accept phase. If you receive an email requesting payment before acceptance or for any other service, this may be a phishing scheme. Learn how to identify phishing emails and protect your accounts at https://explore.plos.org/phishing.

Thank you again for supporting PLOS Computational Biology and open-access publishing. We are looking forward to publishing your work!

With kind regards,

Sharmila Kamatchi

PLOS Computational Biology | Carlyle House, Carlyle Road, Cambridge CB4 3DN | United Kingdom ploscompbiol@plos.org | Phone +44 (0) 1223-442824 | ploscompbiol.org | @PLOSCompBiol
